# Supplementary material for: Borate–Guanosine Hydrogels and Their Hypothetical Participation in the Prebiological Selection of Ribonucleoside Anomers: A Computational (DFT) Study
Source: Int J Mol Sci. 2025 Dec 16;26(24):12103. doi: 10.3390/ijms262412103 (PMC12733540; doi:10.3390/ijms262412103)
Supplement: Supplementary file 1 [file ijms-26-12103-s001.zip › ijms-3989856-supplementary.pdf]

Supporting Information

Borate-Guanosine Hydrogels and Their Hypothetical Participation in the Prebiological Selection of Nucleoside Anomers:

A Computational (DFT) Study

Ana Franco , Adelino M. Galvão *, José A. L. da Silva *

Centro de Química Estrutural, Institute of Molecular Sciences, Departamento de Engenharia Química, Instituto Superior Técnico, Universidade de Lisboa, Av. Rovisco Pais, 1049-001 Lisboa, Portugal; anacfbanha@tecnico.ulisboa.pt

* E-mail: adelino@tecnico.ulisboa.pt, pcd1950@tecnico.ulisboa.pt

Table of Contents

Computational Methods …… 3

Optimized Geometries…… 3

S1. Diesters…… 3

S1.1. Anti Conformation…… 3

S1.2. Syn Conformation…… 11

S2. G2…… 20

S2.1. Anti Conformation…… 20

S2.1.1. Aβ_1-G2…… 20

S2.1.2. Aγ_1-G2…… 26

S2.1.3. Aα_2-G2…… 32

S2.2. Syn Conformation…… 38

S2.2.1. Sβ_1-G2…… 38

S2.2.2. Sγ_2-G2…… 44

S2.2.3. Sα_1-G2…… 50

S3. G3…… 56

S3.1. Anti Conformation…… 56

S3.2. Syn Conformation…… 64

S4. G4…… 72

S4.1. Anti Conformation…… 72

S4.2. Syn Conformation…… 84

S5. Sβ-G4 with K^+…… 96

Computational Methods

All initial structures (diesters, G2, G3 and G4) were constructed with Chemcraft version 1.8 [41]. All theoretical calculations were of the DFT type, carried out with GAMESS-US version R3 [42], using the implemented version of the B3LYP functional. A 6-31G** basis set was used for optimization of smaller structures (diesters), and cal-culation of single point energies of larger structures (G2, G3 and G4) after optimization with a 3-21G basis set. Mulliken and Löwdin atomic charges were performed with Chemcraft version 1.8 [41].

Optimized Geometries

Atoms of different elements are coloured according to CPK (Corey–Pauling–Koltun) colour scheme, where white is hydrogen, black is carbon, red is oxygen, blue is nitrogen, beige is boron and violet is potassium.

S1. Diesters

S1.1. Anti Conformation

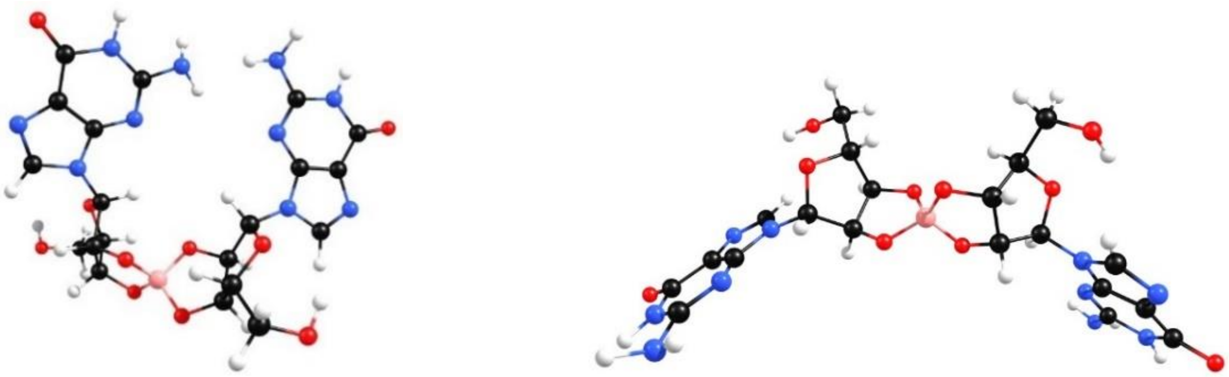

Figure S1. Optimized geometry of borate-guanosine diesters with two β-guanosines in anti conformation: **Aβ$_1$** (left) and **Aβ$_2$** (right).

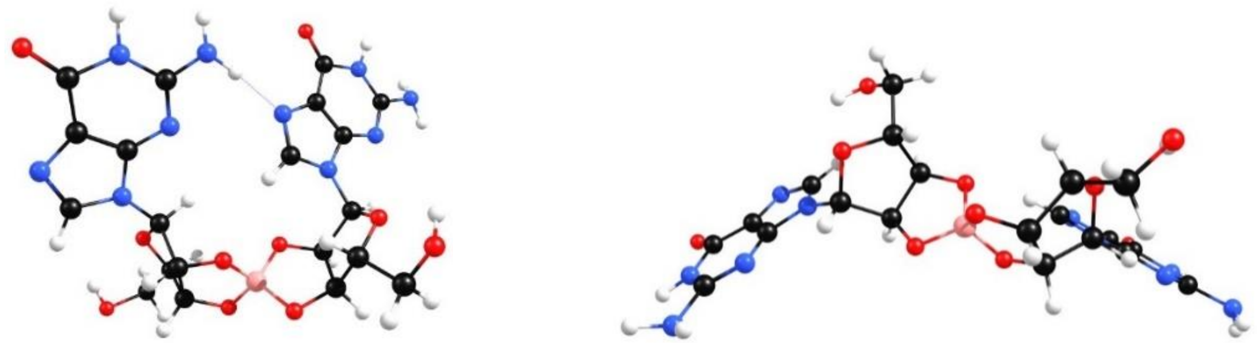

Figure S2. Optimized geometry of borate-guanosine diester with one α-guanosine and one β-guanosine in anti conformation: **Aγ1** (left) and **Aγ2** (right).

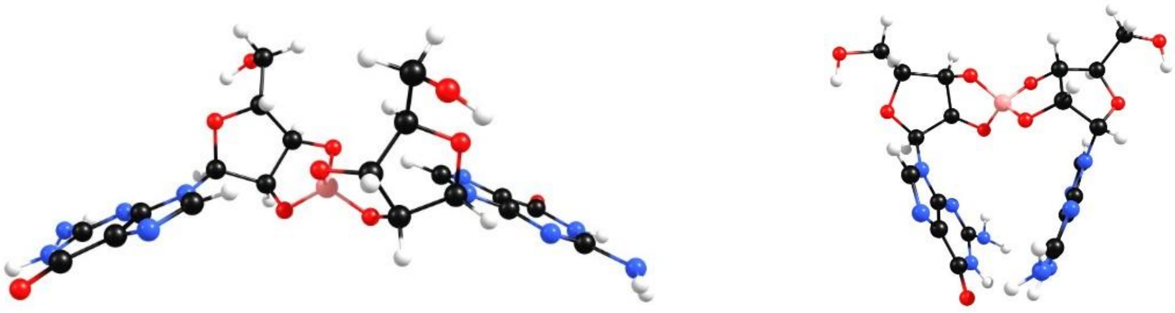

Figure S3. Optimized geometry of borate-guanosine diester with two α-guanosines in anti conformation: **Aα1** (left) and **Aα2** (right).

Table S1. Optimized cartesian coordinates for **Aβ1** and **Aβ2** diesters.

	**Aβ1**				**Aβ2**			
**Number**	**Atom**	**z**	**y**	**z**	**Atom**	**x**	**y**	**z**
1	C	3.354573	-6.241233	0.551516	C	3.725856	-5.908265	0.903710
2	C	2.619954	-6.884581	-0.665258	C	2.726168	-6.626153	-0.036226
3	C	6.490286	-3.381242	1.488181	C	7.755343	-4.532705	0.084677
4	C	1.637450	-7.909541	-0.023661	C	1.635592	-7.198680	0.903928

5	C	1.618056	-7.502927	1.467366	C	1.774163	-6.314710	2.166518
6	C	5.719083	-5.374330	0.860558	C	6.254234	-6.040461	0.732647
7	C	1.451870	-8.647817	2.457789	C	1.515753	-7.025417	3.489933
8	C	6.968722	-6.000924	0.830080	C	7.181943	-7.046461	1.021129
9	C	8.144470	-5.236441	1.147454	C	8.581247	-6.787482	0.810511
10	C	5.551658	-7.499548	0.327385	C	5.270816	-7.865111	1.460351
11	H	5.070400	-8.432571	0.068142	H	4.459244	-8.508724	1.772629
12	H	3.115689	-5.174372	0.598451	H	3.930142	-4.901002	0.531398
13	H	8.554919	-3.280156	1.650948	H	9.707934	-5.228432	0.065068
14	H	6.983700	-1.696471	2.521839	H	8.987422	-2.911516	-0.042348
15	H	5.399513	-1.756905	1.931561	H	7.373091	-2.658284	-0.457065
16	H	3.357748	-7.354606	-1.336876	H	3.249123	-7.397157	-0.625952
17	H	1.987446	-8.947186	-0.113080	H	1.798983	-8.253444	1.167742
18	H	0.827343	-6.753735	1.610292	H	1.100809	-5.453946	2.057408
19	H	1.340368	-8.245354	3.478417	H	1.484007	-6.292489	4.312827
20	H	0.538613	-9.198905	2.209820	H	0.541075	-7.522679	3.442820
21	H	3.328284	-8.996516	2.389110	H	3.341604	-7.566909	3.629450
22	N	7.765475	-3.890168	1.478740	N	8.752707	-5.445999	0.320405
23	N	6.367333	-2.035347	1.794076	N	8.147038	-3.310896	-0.441128
24	N	5.429221	-4.082000	1.180805	N	6.487521	-4.776001	0.284886
25	N	6.841061	-7.334151	0.492691	N	6.544004	-8.182167	1.478573
26	N	4.809716	-6.345706	0.522051	N	5.025278	-6.576318	1.014991
27	O	2.915364	-6.923527	1.727473	O	3.149435	-5.865921	2.206674
28	O	1.816161	-5.983927	-1.350440	O	2.048256	-5.716960	-0.843807
29	O	0.410779	-7.735152	-0.656723	O	0.426922	-7.026647	0.239209
30	O	2.539794	-9.561001	2.390528	O	2.495483	-8.025190	3.748371
31	O	9.327932	-5.541111	1.173489	O	9.569076	-7.490041	0.967129
32	B	0.370595	-6.410619	-1.268886	B	0.592496	-5.963860	-0.791670
33	C	-0.107545	-3.265971	-1.619241	C	-2.321751	-6.275999	-2.784268
34	C	-0.982242	-4.520406	-1.343815	C	-0.992711	-5.538007	-2.493634

35	C	2.470565	-0.526221	0.438937	C	-3.335881	-8.220115	-6.518653
36	C	-1.011007	-5.226191	-2.740113	C	-1.286986	-4.671343	-1.243971
37	C	-0.331343	-4.219646	-3.704905	C	-2.473782	-5.419067	-0.589648
38	C	0.487263	-1.249358	-0.249595	C	-3.297151	-6.494892	-5.114828
39	C	-1.061716	-3.946961	-5.012613	C	-3.494508	-4.531239	0.113446
40	C	-0.244125	-0.178036	0.266945	C	-3.983412	-5.613812	-5.956832
41	C	0.452429	0.862660	0.977308	C	-4.400769	-6.071697	-7.255503
42	C	-1.652063	-1.452659	-0.688559	C	-3.555347	-4.522119	-4.183812
43	H	-2.557621	-1.888835	-1.086036	H	-3.491092	-3.758569	-3.420243
44	H	0.936198	-3.512776	-1.397752	H	-2.129614	-7.337222	-2.961672
45	H	2.403880	1.220052	1.553442	H	-4.184752	-7.799590	-8.361776
46	H	4.374843	0.200813	0.425178	H	-3.727453	-10.006649	-7.423029
47	H	4.193690	-1.425825	0.067704	H	-2.619350	-10.037584	-6.154334
48	H	-1.981700	-4.212213	-1.000889	H	-0.683356	-4.955379	-3.377185
49	H	-2.036185	-5.434415	-3.078803	H	-1.575545	-3.637851	-1.481647
50	H	0.687728	-4.582020	-3.898805	H	-2.065790	-6.159753	0.108809
51	H	-0.430580	-3.319885	-5.664886	H	-4.187477	-5.147289	0.709662
52	H	-1.242294	-4.895156	-5.530626	H	-2.967093	-3.859862	0.799647
53	H	-2.135798	-2.648419	-4.124290	H	-4.531462	-4.350938	-1.477900
54	N	1.861878	0.567209	1.001146	N	-4.005739	-7.443612	-7.431141
55	N	3.848829	-0.645127	0.618770	N	-3.003210	-9.504198	-6.924949
56	N	1.829040	-1.460049	-0.205253	N	-2.968136	-7.797303	-5.338819
57	N	-1.587634	-0.325090	-0.021146	N	-4.136171	-4.382091	-5.352617
58	N	-0.425625	-2.072860	-0.855203	N	-3.026000	-5.785309	-3.974672
59	O	-0.280298	-2.954314	-3.002933	O	-3.179853	-6.090647	-1.661972
60	O	-0.406230	-5.419923	-0.462834	O	-0.000773	-6.419849	-2.072655
61	O	-0.283632	-6.405223	-2.581934	O	-0.127427	-4.706532	-0.474758
62	O	-2.322831	-3.332202	-4.786280	O	-4.213496	-3.722669	-0.811258
63	O	0.052483	1.879107	1.525193	O	-4.988235	-5.504978	-8.165387

Table S2. Optimized xyz cartesian coordinates for **Aγ1** and **Aγ2** diesters.

	**Aγ1**				**Aγ2**			
**Number**	**Atom**	**x**	**y**	**z**	**Atom**	**x**	**y**	**z**
1	C	3.719862	-6.241229	-0.411565	C	1.148766	-4.993238	1.035936
2	C	2.598303	-6.841288	-1.313569	C	0.809823	-6.427692	0.561489
3	C	7.175150	-3.706275	-0.492264	C	0.535716	-3.322319	4.989571
4	C	1.827467	-7.808679	-0.364017	C	0.853300	-6.354344	-0.986132
5	C	2.181691	-7.267638	1.035131	C	1.737469	-5.111800	-1.247822
6	C	4.982340	-4.078596	-0.375555	C	-0.075751	-3.784886	2.901676
7	C	2.230653	-8.294765	2.150675	C	1.350593	-4.280957	-2.465953
8	C	4.638128	-2.747741	-0.156774	C	-1.331258	-3.174086	2.984956
9	C	5.667715	-1.752871	-0.071309	C	-1.729141	-2.562081	4.224739
10	C	2.797468	-3.842175	-0.168740	C	-1.181232	-3.942217	1.009068
11	H	1.753891	-4.122636	-0.154682	H	-1.362637	-4.200901	-0.025676
12	H	4.713122	-6.560088	-0.737375	H	1.921521	-5.030174	1.808109
13	H	7.730529	-1.741149	-0.136259	H	-0.925382	-2.377007	6.115873
14	H	9.097797	-3.480061	-1.133253	H	1.436958	-2.554555	6.650715
15	H	8.573174	-5.060810	-0.896374	H	2.297823	-3.736222	5.811984
16	H	3.081421	-7.370086	-2.153680	H	-0.168162	-6.737991	0.962936
17	H	2.178897	-8.849268	-0.456626	H	-0.135265	-6.220433	-1.445138
18	H	1.488415	-6.456954	1.297837	H	2.778837	-5.448075	-1.331337
19	H	1.221087	-8.676517	2.334951	H	2.132791	-3.533601	-2.676795
20	H	2.860786	-9.140581	1.823771	H	1.272396	-4.940917	-3.336289
21	H	3.490061	-7.226911	3.124693	H	0.165164	-3.183290	-1.445403
22	N	6.948737	-2.370630	-0.268321	N	-0.674053	-2.706534	5.192105
23	N	8.503756	-4.097330	-0.593118	N	1.385333	-3.392000	6.084075
24	N	6.226812	-4.602357	-0.563569	N	0.890523	-3.869448	3.857659
25	N	3.271295	-2.623922	-0.024237	N	-2.007101	-3.281940	1.786256
26	N	3.798227	-4.776951	-0.392647	N	0.012513	-4.279225	1.626918

27	O	3.526682	-6.748071	0.903169	O	1.588549	-4.247995	-0.095313
28	O	1.638902	-5.944941	-1.756274	O	0.087357	-3.648742	-2.292207
29	O	0.486271	-7.699834	-0.695560	O	-2.757344	-1.988879	4.553319
30	O	2.709620	-7.744447	3.371442	C	3.243025	-10.924530	-1.901217
31	O	5.609132	-0.549059	0.143476	C	3.326907	-10.165287	-0.546423
32	B	0.251055	-6.470306	-1.467934	C	1.822306	-14.864494	-2.872156
33	C	-1.185838	-3.481459	-2.071411	C	4.377960	-9.029201	-0.818329
34	C	-1.511515	-4.907327	-1.544498	C	4.710681	-9.159145	-2.321254
35	C	0.397274	-0.400185	0.443659	C	1.581796	-12.670130	-2.590969
36	C	-1.527885	-5.777249	-2.840265	C	6.085804	-9.762338	-2.613538
37	C	-1.220146	-4.771688	-3.977537	C	0.236881	-12.624194	-2.961449
38	C	-1.254351	-1.424322	-0.643199	C	-0.418256	-13.841012	-3.355172
39	C	-2.021991	-4.934642	-5.260828	C	0.779218	-10.613095	-2.491636
40	C	-2.260351	-0.518583	-0.297803	H	0.785562	-9.545961	-2.309515
41	C	-1.908836	0.612592	0.517636	H	3.868700	-11.826113	-1.869533
42	C	-3.197195	-2.005149	-1.500776	H	0.134935	-15.814478	-3.606585
43	H	-3.906230	-2.590292	-2.069163	H	2.062188	-16.865282	-2.572182
44	H	-0.112318	-3.302565	-1.943405	H	3.451410	-15.927551	-2.467034
45	H	-0.216993	1.298370	1.466430	H	3.646150	-10.873552	0.234637
46	H	2.056995	0.596289	1.112780	H	5.294233	-9.153920	-0.217315
47	H	2.311776	-1.026779	0.549236	H	4.606757	-8.188731	-2.813345
48	H	-2.480862	-4.890256	-1.023252	H	6.871953	-9.042828	-2.354590
49	H	-2.508536	-6.241460	-3.017512	H	6.242602	-10.656071	-1.980717
50	H	-0.142741	-4.830267	-4.191884	H	5.351425	-10.429293	-4.238203
51	H	-1.636389	-4.241176	-6.026966	N	0.511291	-14.939750	-3.263087
52	H	-1.895302	-5.955797	-5.636534	N	2.552337	-16.048450	-2.917001
53	H	-3.450760	-3.919669	-4.507585	N	2.408327	-13.753002	-2.514970
54	N	-0.507177	0.569378	0.827000	N	-0.241556	-11.329111	-2.898990
55	N	1.668404	-0.314856	0.922345	N	1.924135	-11.377477	-2.281709
56	N	0.056176	-1.418817	-0.316430	O	3.711993	-10.023866	-2.908626

57	N	-3.473046	-0.904182	-0.846068	O	2.154710	-9.524051	-0.187223
58	N	-1.863195	-2.376763	-1.422917	O	3.741813	-7.836824	-0.492049
59	O	-1.551765	-3.468644	-3.451613	O	6.223970	-10.083732	-3.988211
60	O	-0.512116	-5.433999	-0.731423	O	-1.556552	-14.070050	-3.738340
61	O	-0.534712	-6.741008	-2.676044	B	2.291791	-8.059048	-0.287901
62	O	-3.410756	-4.726692	-5.043084	O	1.449617	-7.541671	-1.406751
63	O	-2.584237	1.535553	0.956490	O	1.818590	-7.328100	0.900118

Table S3. Optimized xyz cartesian coordinates for **Aα_1** and **Aα_2** diesters.

	**Aα_1**				**Aα_2**			
**Number**	**Atom**	**x**	**y**	**z**	**Atom**	**x**	**y**	**z**
1	C	4.346948	-0.198286	-0.856405	B	-4.893436	-6.957638	-0.651259
2	C	3.361472	-0.623814	0.273357	O	-5.972388	-6.070589	-0.129350
3	C	6.433292	3.322293	-2.100100	O	-5.383501	-7.377249	-1.972117
4	C	2.915944	-2.060318	-0.128723	C	-7.806628	-7.128027	-2.534655
5	C	4.057152	-2.529681	-1.057949	C	-6.373149	-6.502189	-2.386989
6	C	6.216006	1.463899	-0.894090	C	-6.300186	-10.750228	-4.239067
7	C	3.647400	-3.481956	-2.173637	C	-6.539084	-5.453553	-1.241829
8	C	7.459844	1.554446	-0.268771	C	-8.042382	-5.230578	-1.109282
9	C	8.324592	2.656190	-0.592114	C	-7.447507	-9.601710	-2.731291
10	C	6.546482	-0.216118	0.497783	C	-8.540608	-3.943476	-1.762553
11	H	6.321878	-1.115759	1.054937	C	-7.739331	-10.698961	-1.906668
12	H	3.927371	0.599949	-1.474164	C	-7.269443	-11.984904	-2.307646
13	H	8.268281	4.260718	-1.890012	C	-8.438810	-8.978909	-0.869923
14	H	6.235955	5.212169	-2.832998	H	-8.865468	-8.297430	-0.150341
15	H	5.054532	4.111804	-3.292000	H	-8.114626	-7.144583	-3.586786
16	H	2.528996	0.096485	0.300420	H	-6.069037	-12.735078	-3.806689
17	H	1.962010	-2.081347	-0.670343	H	-5.122901	-9.995625	-5.625918

18	H	4.846876	-2.980584	-0.442107	H	-6.121652	-6.034314	-3.350829
19	H	4.546582	-3.843197	-2.699894	H	-6.027779	-4.506501	-1.496235
20	H	3.140853	-4.348840	-1.735376	H	-8.351880	-5.268688	-0.059752
21	H	3.129855	-1.985410	-3.229958	H	-8.219951	-3.077700	-1.171592
22	N	7.677359	3.509400	-1.556431	H	-8.085984	-3.845934	-2.764836
23	N	6.031359	4.246179	-3.060547	H	-10.203704	-4.801474	-2.128846
24	N	5.654627	2.318793	-1.797145	N	-6.594773	-11.904485	-3.549507
25	N	7.648083	0.490166	0.591836	N	-5.465322	-10.900543	-5.325400
26	N	5.625207	0.330191	-0.393013	N	-6.774265	-9.573166	-3.905284
27	O	4.540931	-1.330134	-1.705187	N	-8.363267	-10.281795	-0.743622
28	O	3.939353	-0.762366	1.532247	N	-7.916590	-8.507384	-2.062212
29	O	2.843182	-2.780522	1.063671	O	-8.712350	-6.329805	-1.793605
30	O	2.744838	-2.863491	-3.080829	O	-9.957531	-3.909944	-1.836390
31	O	9.447332	2.953265	-0.209152	O	-7.319101	-13.084109	-1.726048
32	B	3.772964	-2.133615	2.035215	H	-4.698373	-11.547716	-5.129847
33	O	5.101684	-2.809708	2.109719	C	-2.226504	-8.743671	0.587723
34	O	3.228992	-2.239719	3.396936	C	-3.430463	-7.805268	0.945952
35	C	3.276256	-4.324757	4.754393	C	-4.571939	-12.257699	-0.175209
36	C	4.016183	-3.078861	4.181340	C	-2.951873	-6.404216	0.464525
37	C	0.377773	-5.157429	7.792135	C	-1.433040	-6.508121	0.354422
38	C	5.068911	-3.675589	3.202615	C	-3.128511	-10.676312	-0.746691
39	C	4.480446	-5.063843	2.868649	C	-0.663625	-5.810613	1.471133
40	C	0.985120	-4.582751	5.729129	C	-2.992211	-11.085112	-2.082451
41	C	5.500109	-6.158739	2.583198	C	-3.716308	-12.236714	-2.511987
42	C	-0.311183	-4.485617	5.222095	C	-1.884074	-9.280021	-1.902409
43	C	-1.422412	-4.768296	6.088152	H	-1.271298	-8.411369	-2.087448
44	C	0.988726	-3.994557	3.602107	H	-2.045823	-9.474947	1.382285
45	H	1.394208	-3.688886	2.646935	H	-5.125280	-13.505153	-1.699825
46	H	3.498438	-4.469793	5.814662	H	-6.343093	-13.132497	0.140429
47	H	-1.664153	-5.396666	8.040968	H	-5.697367	-12.279763	1.442475

48	H	1.596775	-5.422533	9.336856	H	-3.557986	-7.852814	2.038509
49	H	4.468684	-2.522974	5.017377	H	-3.239314	-5.616666	1.185536
50	H	6.062381	-3.791243	3.654069	H	-1.093561	-6.145772	-0.622595
51	H	3.788548	-4.956914	2.022739	H	-0.748523	-4.723596	1.359554
52	H	4.979682	-7.067768	2.238687	H	-1.112026	-6.082460	2.443070
53	H	6.165402	-5.824701	1.779672	H	0.736343	-7.097050	1.303240
54	H	5.664814	-6.460631	4.456276	N	-4.448131	-12.794724	-1.436743
55	N	-0.934973	-5.104308	7.402631	N	-5.493502	-12.870637	0.645229
56	N	0.621145	-5.560134	9.102298	N	-3.867156	-11.232659	0.242027
57	N	1.380119	-4.897448	6.996534	N	-2.205627	-10.191303	-2.787964
58	N	-0.284776	-4.124077	3.889104	N	-2.401584	-9.524151	-0.641209
59	N	1.820344	-4.258242	4.688635	O	-1.083507	-7.923476	0.452100
60	O	3.766990	-5.471728	4.059139	O	-4.617599	-8.060821	0.281757
61	O	6.300910	-6.435952	3.724094	O	-3.586138	-6.243619	-0.766503
62	O	-2.629436	-4.775725	5.892178	O	0.717822	-6.136430	1.434486
63	H	0.005102	-5.150459	9.794798	O	-3.823560	-12.754201	-3.639068

S1.2. Syn Conformation

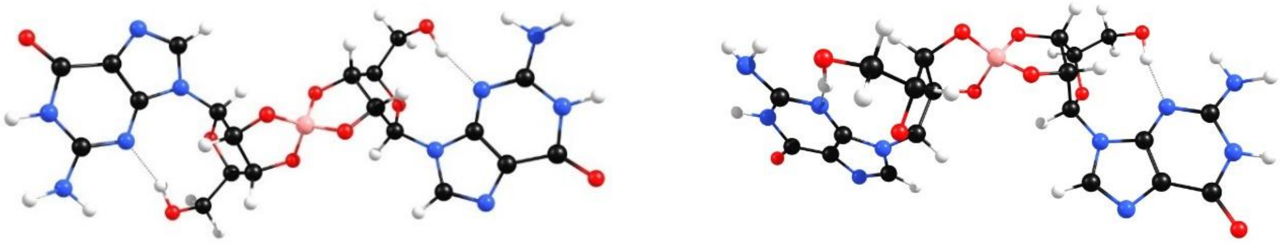

Figure S4. Optimized geometry of borate-guanosine diester with two β-guanosines in syn conformation: **Sβ$_1$** (left) and **Sβ$_2$** (right).

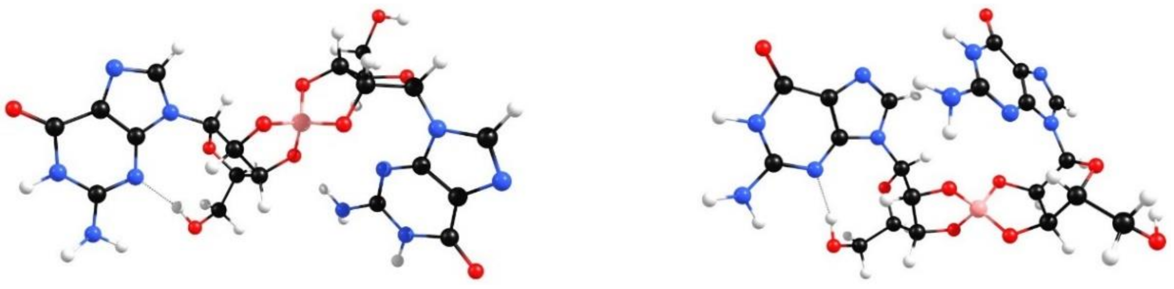

Figure S5. Optimized geometry of borate-guanosine diester with one α-guanosine and one β-guanosine in syn conformation: **Sγ1** (left) and **Sγ2** (right).

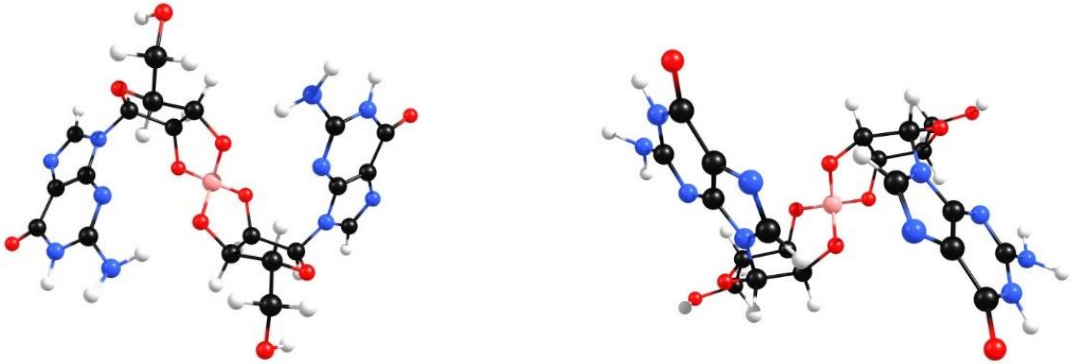

Figure S6. Optimized geometry of borate-guanosine diester with two α-guanosines in syn conformation: **Sα1** (left) and **Sα2** (right).

Table S4. Optimized xyz cartesian coordinates for **Sβ1** and **Sβ2** diesters.

	**Sβ1**				**Sβ2**			
**Number**	**Atom**	**x**	**y**	**z**	**Atom**	**x**	**y**	**z**
1	O	-2.572080	-0.235477	1.630004	O	-2.970582	-1.280446	2.965605
2	O	-3.967398	-1.965831	2.396165	O	-0.797851	-0.500823	2.550577
3	B	-2.949207	-1.655899	1.369749	B	-1.941612	-1.223816	1.916945
4	C	-1.527137	-1.384817	-1.377309	C	-1.388126	-0.538486	-1.501246

5	C	-2.487410	-2.444409	-0.757439	C	-2.271398	-1.250265	-0.437938
6	C	-1.223516	-3.555983	-5.154853	C	-2.021728	-3.620877	-4.551711
7	C	-1.543695	-3.180419	0.222226	C	-1.430320	-2.473398	0.017692
8	C	-0.127719	-2.976413	-0.349215	C	0.002558	-2.116836	-0.449299
9	C	-1.726139	-1.774869	-3.888050	C	-2.155609	-1.541491	-3.728915
10	C	0.488494	-4.205843	-1.016507	C	0.853677	-3.288831	-0.919914
11	C	-2.200834	-0.988612	-4.940610	C	-2.893636	-1.021090	-4.795287
12	C	-2.190174	-1.528526	-6.275629	C	-3.252933	-1.889237	-5.886411
13	C	-2.376444	0.193808	-3.185873	C	-2.605347	0.581843	-3.435491
14	H	-2.578279	0.985005	-2.476388	H	-2.614038	1.543958	-2.940430
15	H	-1.598698	-0.464339	-0.783778	H	-1.267197	0.511467	-1.204488
16	H	-1.544305	-3.270554	-7.179787	H	-2.909687	-3.856663	-6.407954
17	H	-1.145999	-5.412802	-6.003377	H	-2.161807	-5.631451	-4.895928
18	H	-0.455741	-5.259851	-4.460711	H	-1.098071	-5.151429	-3.658063
19	H	-2.889648	-3.091347	-1.548149	H	-3.240943	-1.526462	-0.884351
20	H	-1.777530	-4.253534	0.301282	H	-1.750038	-3.410963	-0.453535
21	H	0.534185	-2.625571	0.453276	H	0.513063	-1.603216	0.376474
22	H	1.454725	-3.912575	-1.462148	H	1.841726	-2.910718	-1.232347
23	H	0.696180	-4.959258	-0.245762	H	1.009490	-3.959463	-0.065611
24	H	-0.680278	-4.084122	-2.565463	H	-0.254065	-3.398707	-2.511377
25	N	-1.660366	-2.870237	-6.257148	N	-2.756410	-3.218640	-5.636924
26	N	-0.671735	-4.808405	-5.345226	N	-1.545192	-4.914500	-4.538843
27	N	-1.254839	-3.049745	-3.949101	N	-1.710183	-2.815488	-3.567361
28	N	-2.606049	0.247606	-4.476915	N	-3.162749	0.318289	-4.592932
29	N	-1.840986	-1.008399	-2.759892	N	-1.974193	-0.503579	-2.852947
30	O	-0.222836	-1.929939	-1.356069	O	-0.145210	-1.203842	-1.568865
31	O	-3.493817	-1.878750	0.008384	O	-2.427238	-0.473409	0.704941
32	O	-1.739983	-2.510155	1.436855	O	-1.555391	-2.539204	1.403374
33	O	-0.365500	-4.806030	-1.976486	O	0.250003	-4.036180	-1.965454
34	O	-2.529023	-1.047518	-7.346208	O	-3.869295	-1.676630	-6.919599

35	C	-3.045275	-1.058280	4.482406	C	-1.863198	1.608013	3.223788
36	C	-4.033880	-0.906756	3.287941	C	-1.302004	0.210133	3.627245
37	C	-5.919138	0.517504	7.373335	C	-0.469470	3.530439	6.897000
38	C	-3.450797	0.327509	2.564243	C	-2.581104	-0.536188	4.085932
39	C	-2.709726	1.123363	3.652481	C	-3.589103	0.572034	4.451877
40	C	-4.550656	-1.053236	6.541664	C	-0.550912	3.249085	4.673145
41	C	-3.417186	2.385878	4.142508	C	-3.869302	0.729594	5.945709
42	C	-4.827031	-2.008169	7.522406	C	0.376540	4.250440	4.375822
43	C	-5.754221	-1.680561	8.574319	C	0.963617	5.002269	5.454510
44	C	-3.408185	-2.891437	6.213020	C	-0.237816	3.434923	2.514818
45	H	-2.720898	-3.566482	5.721026	H	-0.360793	3.209992	1.463943
46	H	-2.243612	-1.746562	4.185187	H	-2.056488	1.604814	2.143569
47	H	-6.844671	-0.019342	9.144267	H	0.753752	5.084126	7.508972
48	H	-7.414240	1.880927	7.662724	H	-0.212451	3.293161	8.910725
49	H	-6.195096	2.319397	6.565611	H	-1.522323	2.465037	8.214972
50	H	-5.053613	-0.754144	3.665891	H	-0.556131	0.328426	4.425216
51	H	-4.231753	0.942697	2.089573	H	-2.394246	-1.184275	4.955369
52	H	-1.710361	1.387788	3.282587	H	-4.531260	0.379744	3.921913
53	H	-2.839527	2.801961	4.985901	H	-4.517699	1.610982	6.089236
54	H	-3.406127	3.131514	3.336963	H	-4.425698	-0.149814	6.294311
55	H	-4.780730	1.396608	5.109151	H	-2.124446	1.505376	6.301580
56	N	-6.256344	-0.340732	8.385774	N	0.446294	4.533023	6.717224
57	N	-6.440681	1.796085	7.401564	N	-0.912229	3.277109	8.180418
58	N	-5.081731	0.193093	6.421736	N	-0.978893	2.851128	5.901624
59	N	-4.097484	-3.159043	7.296986	N	0.559713	4.351305	3.010766
60	N	-3.635099	-1.626509	5.700597	N	-0.944257	2.726872	3.470309
61	O	-2.549214	0.228418	4.790758	O	-3.040618	1.831052	3.970389
62	O	-4.772220	2.167549	4.498268	O	-2.688746	0.821366	6.727216
63	O	-6.141903	-2.319914	9.540171	O	1.768867	5.920687	5.456927

Table S5. Optimized xyz cartesian coordinates for **Sγ1** and **Sγ2** diesters.

	**Sγ1**				**Sγ2**			
**Number**	**Atom**	**x**	**y**	**z**	**Atom**	**x**	**y**	**z**
1	O	-1.220139	-1.177980	1.795210	O	-1.596982	-2.712383	2.280914
2	O	-1.194536	-3.159076	3.060571	O	-1.539383	-0.679903	1.105239
3	B	-0.806254	-2.604370	1.749013	B	-1.067120	-2.087454	1.061325
4	C	-0.519897	-2.302546	-1.377460	C	-0.041142	-1.982762	-1.970221
5	C	-0.560643	-3.507767	-0.389018	C	-0.404781	-3.106758	-0.954409
6	C	-0.039212	-4.900499	-4.856406	C	0.567995	-5.058789	-5.015165
7	C	0.847841	-3.453092	0.243649	C	0.811964	-3.060171	-0.012821
8	C	1.722159	-2.699109	-0.778273	C	1.966545	-2.522681	-0.878429
9	C	-1.092741	-3.345455	-3.630529	C	-0.395933	-3.220929	-4.170763
10	C	2.731476	-3.567004	-1.528219	C	2.981908	-3.569791	-1.333371
11	C	-2.166850	-3.184614	-4.508534	C	-1.356767	-3.094603	-5.175662
12	C	-2.202090	-3.963251	-5.719223	C	-1.375463	-4.054703	-6.248742
13	C	-2.512708	-1.848951	-2.894743	C	-1.685551	-1.482777	-3.829091
14	H	-2.928415	-1.112799	-2.219844	H	-2.065750	-0.600696	-3.332210
15	H	-0.929980	-1.421743	-0.866582	H	-0.446047	-1.030391	-1.604138
16	H	-0.970050	-5.331030	-6.655283	H	-0.236780	-5.682699	-6.817980
17	H	0.821683	-6.617603	-5.552023	H	1.273027	-6.947180	-5.354191
18	H	1.682870	-5.776883	-4.354785	H	2.086606	-6.039077	-4.172360
19	H	-0.748346	-4.435739	-0.945904	H	-0.513327	-4.063656	-1.481227
20	H	1.258946	-4.453543	0.440446	H	1.061649	-4.051170	0.399232
21	H	2.250182	-1.882487	-0.266686	H	2.478340	-1.719351	-0.333275
22	H	3.234197	-2.943493	-2.287633	H	3.710206	-3.082056	-2.003595
23	H	3.498629	-3.906302	-0.819809	H	3.532150	-3.936332	-0.457678
24	H	1.352969	-4.422411	-2.602726	H	1.761190	-4.348340	-2.635678
25	N	-1.044314	-4.821107	-5.783658	N	-0.326256	-5.026129	-6.052487
26	N	1.035724	-5.720960	-5.136007	N	1.548354	-6.028528	-5.033351

27	N	-0.033314	-4.190419	-3.757221	N	0.561326	-4.178327	-4.045574
28	N	-3.049569	-2.237691	-4.026854	N	-2.163613	-1.996339	-4.940211
29	N	-1.320143	-2.482439	-2.592099	N	-0.617711	-2.182907	-3.302944
30	O	0.827375	-2.113501	-1.761812	O	1.364789	-1.955998	-2.078966
31	O	-1.468687	-3.331658	0.638573	O	-1.522648	-2.808483	-0.186008
32	O	0.656473	-2.728719	1.432687	O	0.413240	-2.153383	0.974398
33	O	2.147186	-4.721998	-2.105105	O	2.378136	-4.692774	-1.954476
34	O	-3.005661	-3.993262	-6.638368	O	-2.093360	-4.155822	-7.230506
35	C	-1.590738	-1.988214	5.247920	C	-3.450407	0.322075	2.401548
36	C	-1.977165	-2.246762	3.754511	C	-2.059856	-0.398652	2.362495
37	C	2.377197	-3.518024	4.291567	C	-4.311788	-1.385203	-1.521399
38	C	-1.714418	-0.881321	3.062187	C	-2.340200	-1.785227	3.006665
39	C	-0.677896	-0.217380	3.990621	C	-3.862386	-1.952604	2.842376
40	C	0.543865	-3.363042	5.535761	C	-4.203393	0.270683	-0.042719
41	C	-0.724585	1.302441	4.057183	C	-4.565139	-2.750166	3.930938
42	C	0.931009	-4.308927	6.487283	C	-4.573781	1.285888	-0.929678
43	C	2.218650	-4.944178	6.347957	C	-4.887069	0.934801	-2.290453
44	C	-1.032815	-3.708813	7.023697	C	-4.133828	2.250716	0.908198
45	H	-1.992157	-3.591836	7.512100	H	-3.976801	2.978721	1.694307
46	H	-2.496732	-1.976343	5.866832	H	-3.412315	1.171686	3.094529
47	H	3.691255	-4.995203	4.905435	H	-4.817432	-0.774853	-3.446109
48	H	2.494973	-2.757316	2.476719	H	-3.427272	-3.115983	-1.266218
49	H	-3.042292	-2.536860	3.728978	H	-1.354008	0.207756	2.956896
50	H	-2.614240	-0.251885	3.011298	H	-2.086125	-1.810749	4.076250
51	H	0.321344	-0.565463	3.706497	H	-4.066212	-2.372483	1.852049
52	H	0.128030	1.675978	4.648945	H	-5.621053	-2.906394	3.653876
53	H	-0.631763	1.703102	3.041724	H	-4.089117	-3.733757	4.011556
54	H	-2.112229	1.186085	5.356293	H	-4.643939	-1.181517	5.007735
55	N	2.869853	-4.464165	5.165893	N	-4.685265	-0.474383	-2.486936
56	N	3.068362	-3.311809	3.115425	N	-4.120767	-2.698113	-1.891154

57	N	1.254027	-2.885522	4.491643	N	-4.127216	-1.056401	-0.269407
58	N	-0.076266	-4.512592	7.415104	N	-4.526430	2.523515	-0.309491
59	N	-0.730570	-2.983553	5.881436	N	-3.911540	0.901841	1.142808
60	O	-1.004088	-0.693811	5.322846	O	-4.396899	-0.602772	2.917989
61	O	-1.954838	1.769139	4.597152	O	-4.466703	-2.115362	5.201026
62	O	2.769897	-5.796118	7.034121	O	-5.246999	1.624474	-3.235408
63	H	4.044820	-3.057557	3.184351	H	-3.905416	-2.841041	-2.870759

Table S6. Optimized xyz cartesian coordinates for **Sα_1** and **Sα_2** diesters.

	**Sα_1**				**Sα_2**			
**Number**	**Atom**	**x**	**y**	**z**	**Atom**	**x**	**y**	**z**
1	C	5.204952	-0.780708	-3.370490	B	-5.009109	-6.666549	-0.616181
2	C	4.357821	-1.526783	-2.284152	O	-6.120967	-5.809089	-0.139150
3	C	8.727036	-2.622270	-1.557050	O	-5.397565	-7.047496	-1.996429
4	C	3.926042	-2.836209	-2.992525	C	-7.886376	-7.297538	-2.230825
5	C	5.007735	-3.014177	-4.076464	C	-6.585411	-6.427156	-2.325715
6	C	7.586203	-0.934604	-2.444615	C	-9.478574	-8.329978	1.810191
7	C	4.569620	-3.736181	-5.342894	C	-6.832007	-5.341580	-1.245159
8	C	8.583295	0.027030	-2.266274	C	-8.350866	-5.321058	-1.039001
9	C	9.836628	-0.376133	-1.678169	C	-8.306294	-8.669719	-0.058721
10	C	6.911328	1.062980	-3.063208	C	-9.052592	-4.063823	-1.531415
11	H	6.236694	1.817426	-3.448408	C	-7.861557	-9.945379	0.311823
12	H	4.656980	0.099898	-3.727367	C	-8.247585	-10.491684	1.583523
13	H	10.558544	-2.072822	-0.756479	C	-7.029778	-9.577810	-1.604022
14	H	9.613520	-4.433621	-1.107076	H	-6.453431	-9.646650	-2.515956
15	H	7.872264	-4.336536	-1.043016	H	-8.163749	-7.724818	-3.200531
16	H	3.499987	-0.880767	-2.027986	H	-9.307435	-9.834600	3.225985
17	H	2.937922	-2.762225	-3.467649	H	-10.594804	-6.735468	2.200862

18	H	5.882241	-3.499963	-3.628790	H	-6.561427	-6.013969	-3.346071
19	H	5.437558	-3.879792	-6.007576	H	-6.470812	-4.351239	-1.581078
20	H	4.187362	-4.728039	-5.075804	H	-8.600260	-5.528818	0.005028
21	H	3.814967	-2.111211	-5.989061	H	-8.804274	-3.220399	-0.876717
22	N	9.787511	-1.767179	-1.336702	H	-8.691421	-3.817122	-2.545397
23	N	8.780949	-3.873864	-0.998218	H	-10.621579	-5.097059	-1.880382
24	N	7.645913	-2.258957	-2.194851	N	-9.086897	-9.558576	2.277467
25	N	8.138007	1.277205	-2.662103	N	-10.255017	-7.563120	2.676097
26	N	6.501109	-0.258049	-2.951336	N	-9.137480	-7.850030	0.647903
27	O	5.346616	-1.664028	-4.479432	N	-7.066713	-10.496565	-0.669027
28	O	5.031523	-1.942523	-1.146405	N	-7.767927	-8.446761	-1.305150
29	O	3.940958	-3.832308	-2.013110	O	-8.899139	-6.412477	-1.847909
30	O	3.529516	-3.037923	-6.015433	O	-10.467079	-4.212682	-1.514862
31	O	10.844921	0.270103	-1.421198	O	-7.976725	-11.557470	2.123026
32	B	4.674035	-3.339786	-0.805104	H	-11.003120	-8.069592	3.135341
33	O	5.872046	-4.174202	-0.484724	C	-2.500528	-8.754429	0.164381
34	O	3.846540	-3.412717	0.426319	C	-3.567953	-7.813359	0.822521
35	C	3.795396	-5.132788	2.262483	C	-1.016255	-7.556922	-3.873586
36	C	4.547017	-4.019221	1.458054	C	-2.990846	-6.404141	0.530180
37	C	1.092384	-5.511043	-1.133529	C	-1.493036	-6.627001	0.297196
38	C	5.695147	-4.788608	0.758087	C	-2.293164	-8.681549	-2.430107
39	C	5.138856	-6.223034	0.668661	C	-0.588000	-6.107267	1.405366
40	C	1.569425	-5.347614	1.029895	C	-2.985610	-9.395016	-3.416846
41	C	6.170507	-7.341535	0.678543	C	-2.685328	-9.166031	-4.803258
42	C	0.245613	-5.346956	1.474321	C	-3.783012	-10.050489	-1.564906
43	C	-0.810642	-5.468037	0.500365	H	-4.393940	-10.518661	-0.805297
44	C	1.443055	-5.069703	3.205361	H	-2.407052	-9.703603	0.703331
45	H	1.795970	-4.922933	4.218542	H	-1.461960	-7.892607	-5.869238
46	H	3.988304	-5.006143	3.335046	H	0.575753	-6.887785	-4.950969
47	H	-0.936070	-5.435536	-1.556705	H	0.428071	-6.283515	-3.391849

48	H	2.441593	-5.186324	-2.547455	H	-3.579758	-8.055773	1.896616
49	H	4.916879	-3.272861	2.183048	H	-3.155391	-5.716821	1.381121
50	H	6.628902	-4.790943	1.336742	H	-1.191314	-6.235427	-0.677925
51	H	4.488147	-6.296324	-0.209967	H	-0.571636	-5.011363	1.389239
52	H	5.667616	-8.310174	0.520906	H	-0.992133	-6.421087	2.383910
53	H	6.869215	-7.189773	-0.152080	H	0.667003	-7.492025	1.009694
54	H	6.260659	-7.225734	2.577357	N	-1.646747	-8.184066	-4.917784
55	N	-0.250706	-5.530443	-0.817979	N	-0.063584	-6.600210	-4.219130
56	N	1.445963	-5.389126	-2.452946	N	-1.283754	-7.783167	-2.618676
57	N	2.032201	-5.512469	-0.225974	N	-3.911341	-10.246531	-2.855160
58	N	0.188504	-5.170414	2.846958	N	-2.812017	-9.122962	-1.234237
59	N	2.341201	-5.158038	2.151328	O	-1.281442	-8.076173	0.271406
60	O	4.353033	-6.386118	1.876353	O	-4.835076	-7.840384	0.277610
61	O	6.925761	-7.354821	1.883240	O	-3.686159	-5.996830	-0.609792
62	O	-2.027660	-5.489937	0.636315	O	0.752168	-6.554466	1.240575
63	H	1.004422	-5.974184	-3.146639	O	-3.159863	-9.647690	-5.824668

S2. G2

S2.1. Anti Conformation

S2.1.1. Aβ1-G2

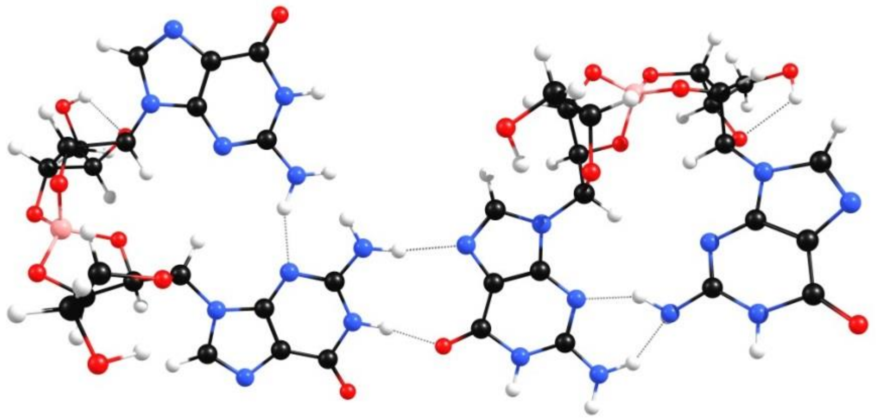

Figure S7. Optimized geometry of **Aβ1-G21**, formed by two borate-guanosine diesters with two β-guanosines in anti conformation.

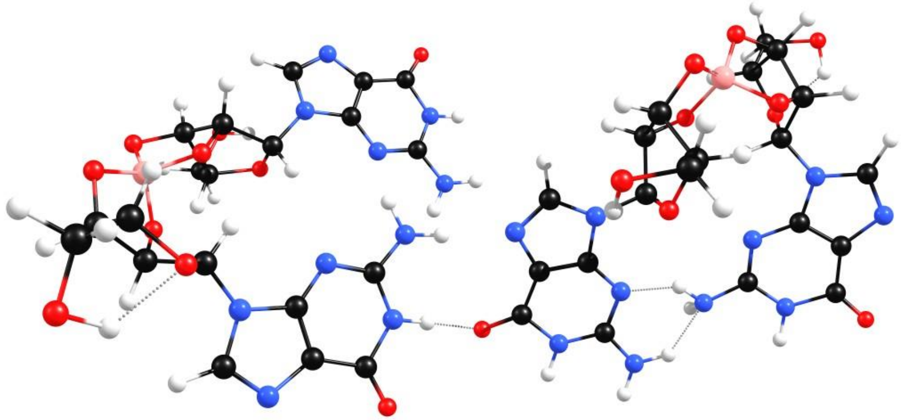

Figure S8. Optimized geometry of **Aβ1-G22**, formed by two borate-guanosine diesters with two β-guanosines in anti conformation.

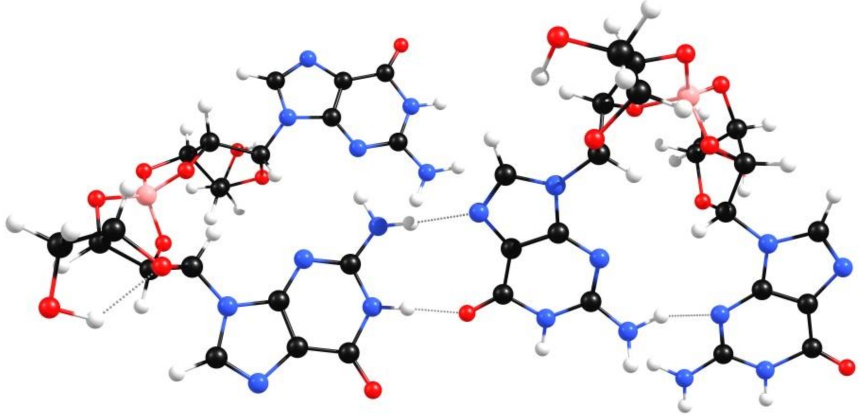

Figure S9. Optimized geometry of **Aβ1-G2$_3$**, formed by two borate-guanosine diesters with two β-guanosines in anti conformation.

Table S7. Optimized xyz cartesian coordinates for **Aβ1-G2** structures.

	Aβ1-G2$_1$				Aβ1-G2$_2$				Aβ1-G2$_3$			
**Number**	**Atom**	**x**	**y**	**z**	**Atom**	**x**	**y**	**z**	**Atom**	**x**	**y**	**z**
1	C	-1.070212	4.612011	4.421065	C	1.415714	5.985867	4.855588	C	1.955943	5.639382	3.901726
2	C	-2.318695	5.539446	4.527547	C	0.460772	6.434221	5.996430	C	1.493244	6.871214	4.730833
3	C	1.826087	1.840884	2.662552	C	5.268124	4.459958	3.535917	C	4.900642	3.035918	2.063665
4	C	-2.881858	5.279176	5.958791	C	-0.525600	7.437392	5.321648	C	0.106812	7.256169	4.125090
5	C	-1.638381	4.808224	6.742313	C	-0.521045	6.989131	3.842959	C	-0.403113	5.922572	3.535723
6	C	-0.252582	2.679686	2.989749	C	3.929037	5.988451	4.511970	C	3.931169	5.038130	2.435911
7	C	-1.981341	3.810769	7.842415	C	-0.696373	8.154175	2.875989	C	-1.241231	6.121377	2.277930
8	C	-0.894118	1.640974	2.311616	C	4.915247	6.976240	4.613491	C	4.616106	5.703225	1.413469
9	C	-0.125891	0.596347	1.701953	C	6.243464	6.709102	4.156310	C	5.573712	4.998681	0.619067
10	C	-2.469264	2.888748	3.102378	C	3.089165	7.878688	5.333077	C	3.230497	7.140923	2.238190
11	H	-3.421565	3.303399	3.378264	H	2.344838	8.559334	5.707503	H	2.644396	8.020300	2.437623
12	H	-0.189324	5.188595	4.133107	H	1.498234	4.898488	4.816509	H	2.266173	4.818768	4.550291

13	H	1.880062	0.015770	1.642891	H	7.212665	5.141139	3.196397	H	6.241851	3.037050	0.462748
14	H	3.837128	1.175067	2.488968	H	6.407243	2.901844	2.754554	H	5.783081	1.155008	1.907757
15	H	3.498672	2.552242	3.533989	H	4.676846	2.624947	2.900927	H	4.463224	1.319062	3.056637
16	H	-3.037400	5.325268	3.728410	H	1.034260	6.867413	6.826412	H	2.237367	7.675774	4.671040
17	H	-3.652174	4.504581	6.003853	H	-0.218003	8.484903	5.385898	H	0.157095	8.009751	3.334083
18	H	-1.069842	5.676100	7.085039	H	-1.241580	6.180537	3.704275	H	-0.898677	5.343981	4.318094
19	H	-1.074500	3.552734	8.410966	H	-0.743386	7.779947	1.841715	H	-1.645534	5.155435	1.938456
20	H	-2.714342	4.259903	8.518565	H	-1.624532	8.682018	3.111646	H	-2.068671	6.800238	2.501495
21	H	-2.016747	2.515007	6.413148	H	1.191575	8.456318	3.140628	H	0.458540	6.248450	1.375854
22	N	1.280196	0.791096	1.968947	N	6.306588	5.367355	3.600718	N	5.627393	3.608111	1.039397
23	N	3.167700	1.862275	2.866833	N	5.479458	3.263474	2.929778	N	5.028458	1.705154	2.294675
24	N	1.086697	2.850687	3.150319	N	4.063701	4.724812	4.032515	N	4.070311	3.743859	2.824165
25	N	-2.284413	1.792772	2.390531	N	4.367182	8.154860	5.136684	N	4.164608	7.025727	1.310130
26	N	-1.255276	3.498777	3.483941	N	2.763373	6.554114	4.984025	N	3.054963	5.955344	2.978854
27	O	-0.860177	4.011515	5.753263	O	0.869255	6.512159	3.592720	O	0.822469	5.222695	3.056948
28	O	-1.977191	6.935220	4.542719	O	-0.392687	5.368405	6.440301	O	1.190406	6.540703	6.095345
29	O	-3.360835	6.547888	6.443935	O	-1.809166	7.212024	5.934177	O	-0.712213	7.681982	5.229571
30	O	-2.590327	2.633270	7.228651	O	0.414441	9.086256	3.063568	O	-0.404219	6.747343	1.256215
31	O	-0.494201	-0.388106	1.046810	O	7.265573	7.406170	4.140662	O	6.291670	5.362667	-0.320167
32	B	-2.480421	7.561632	5.855834	B	-1.856811	5.773154	6.234227	B	-0.286832	6.867331	6.374262
33	C	0.186551	8.889570	4.963898	C	-1.589509	2.713680	5.800248	C	0.543197	4.313823	7.967327
34	C	-0.762508	9.104701	6.177979	C	-2.782388	3.702871	5.671082	C	-0.788076	5.106929	7.825130
35	C	3.292915	6.071940	3.930012	C	1.968548	1.253137	3.846323	C	2.973037	1.377153	5.929935
36	C	-2.014025	9.825489	5.527015	C	-3.133380	4.034275	7.174464	C	-0.513988	6.422018	8.660620
37	C	-1.607553	10.081153	4.051883	C	-2.310836	3.026398	8.018994	C	0.862118	6.189384	9.338180
38	C	2.526162	7.988863	4.818704	C	-0.242764	1.302897	4.222533	C	1.309857	2.019396	7.294446
39	C	-1.901259	11.497834	3.569919	C	-3.153845	2.240460	9.019684	C	0.864853	6.505738	10.830943
40	C	3.787041	8.543859	5.061543	C	-0.522814	0.158042	3.473779	C	1.033670	0.735056	7.768426
41	C	4.964067	7.809799	4.711606	C	0.545710	-0.537960	2.815242	C	1.792708	-0.380422	7.279421
42	C	2.352477	10.034754	5.684289	C	-2.408461	0.790295	4.323867	C	-0.296604	2.062008	8.839296

43	H	1.871601	10.928989	6.039706	H	-3.441542	0.871226	4.613563	H	-1.034143	2.481058	9.501552
44	H	-0.060858	7.926462	4.515981	H	-0.666888	3.293127	5.860318	H	1.211841	4.624897	7.163849
45	H	5.373691	5.936146	3.917616	H	2.611033	-0.271392	2.586218	H	3.312316	-0.677091	5.868792
46	H	3.853355	4.497932	2.721007	H	4.044618	1.087952	4.101064	H	4.850912	1.079851	5.052511
47	H	2.231601	4.369318	3.349809	H	3.302951	2.643810	4.458297	H	4.000963	2.573855	4.655827
48	H	-0.273717	9.703608	6.954406	H	-3.616948	3.242367	5.131108	H	-1.632380	4.522905	8.207652
49	H	-2.248268	10.774978	6.018152	H	-4.201507	3.910622	7.381554	H	-1.279773	6.594609	9.423385
50	H	-2.046004	9.293187	3.434873	H	-1.467804	3.556569	8.469909	H	1.624052	6.722654	8.763672
51	H	-1.658422	11.586031	2.499527	H	-2.504321	1.586194	9.621570	H	1.880099	6.382963	11.239542
52	H	-2.959681	11.730089	3.718766	H	-3.687936	2.925762	9.684130	H	0.533932	7.535326	10.995362
53	H	-0.252332	11.917558	4.464751	H	-3.588821	1.150072	7.498055	H	0.083413	4.757144	10.983551
54	N	4.583097	6.542519	4.124977	N	1.798358	0.122342	3.081732	N	2.773943	0.073521	6.326715
55	N	3.159642	4.833331	3.380717	N	3.250147	1.734950	3.990058	N	3.969536	1.614916	5.016695
56	N	2.226476	6.782775	4.268407	N	0.962553	1.890874	4.433313	N	2.247406	2.393587	6.387620
57	N	3.656671	9.827115	5.611257	N	-1.891740	-0.144133	3.543353	N	0.016308	0.781584	8.734533
58	N	1.609020	8.940201	5.219095	N	-1.448278	1.714824	4.764086	N	0.455374	2.871753	7.973736
59	O	-0.120780	9.996046	4.031363	O	-1.839067	1.985174	7.066649	O	1.089866	4.720495	9.284936
60	O	-1.265616	7.870001	6.704372	O	-2.404717	4.953626	5.084091	O	-1.028502	5.553136	6.483900
61	O	-3.100608	8.883256	5.621950	O	-2.702011	5.394483	7.384188	O	-0.463511	7.495175	7.698594
62	O	-1.115622	12.423364	4.382774	O	-4.145863	1.477226	8.267103	O	-0.093892	5.613206	11.477984
63	O	6.166420	8.081489	4.829667	O	0.524171	-1.555749	2.106318	O	1.713915	-1.586951	7.556658
64	C	8.785212	-1.176160	1.502148	C	9.062018	-1.399166	4.984841	C	11.269532	-4.757240	2.557866
65	C	9.586756	-0.028318	0.837748	C	9.762936	-0.310848	5.841856	C	12.506635	-3.831438	2.699497
66	C	5.383142	-3.701430	0.225250	C	6.939076	-3.039569	1.559722	C	8.685978	-8.159125	3.314894
67	C	10.147758	0.815834	2.027118	C	9.463128	-0.719311	7.319075	C	12.787915	-3.326043	1.250402
68	C	10.185332	-0.189181	3.205456	C	9.235475	-2.244748	7.224371	C	11.400351	-3.402122	0.576349
69	C	6.342577	-1.800672	1.003067	C	7.253442	-1.527982	3.213454	C	10.690827	-7.194230	2.920856
70	C	9.747508	0.433808	4.528896	C	8.194499	-2.743601	8.216473	C	11.479418	-3.748592	-0.904601
71	C	5.123715	-1.170152	1.266841	C	6.006513	-0.922515	3.048737	C	11.363925	-8.399017	2.696135
72	C	3.900726	-1.824293	0.963717	C	5.122336	-1.380921	2.032579	C	10.668524	-9.642804	2.805042

73	C	6.657922	0.173161	1.992247	C	6.922706	0.038644	4.758048	C	12.797808	-6.830483	2.312923
74	H	7.196794	0.932001	2.535183	H	7.114728	0.646855	5.623332	H	13.678638	-6.273415	2.049708
75	H	8.990890	-2.121330	0.997878	H	9.746481	-1.792231	4.231804	H	10.510164	-4.501136	3.297695
76	H	3.289536	-3.656247	0.241513	H	5.063234	-2.897736	0.637510	H	8.706188	-10.239046	3.153865
77	H	4.608904	-5.514498	-0.413911	H	6.716493	-4.542927	0.145576	H	6.761878	-8.916085	3.577932
78	H	6.387126	-5.387707	-0.332203	H	8.254842	-4.501970	1.033877	H	6.942604	-7.160082	3.643181
79	H	8.940983	0.541506	0.157885	H	9.402525	0.692441	5.577754	H	13.350095	-4.367842	3.152202
80	H	9.539418	1.689763	2.273961	H	8.577530	-0.243678	7.747657	H	13.501247	-3.935878	0.689892
81	H	11.172556	-0.651584	3.239926	H	10.194334	-2.763423	7.282789	H	10.841457	-2.489341	0.789886
82	H	9.967849	-0.240259	5.368232	H	8.091019	-3.836216	8.136613	H	10.471567	-3.733732	-1.346388
83	H	10.269989	1.380407	4.681912	H	8.510563	-2.484728	9.230139	H	12.106057	-3.012516	-1.414822
84	H	7.938113	-0.156874	4.177253	H	6.843253	-2.136827	6.965769	H	11.664700	-5.584395	-0.326556
85	N	4.142128	-3.135371	0.438657	N	5.691063	-2.497494	1.332516	N	9.265281	-9.389810	3.111039
86	N	5.443683	-4.974729	-0.229888	N	7.328149	-4.107524	0.822346	N	7.350888	-8.095236	3.572838
87	N	6.516388	-3.035029	0.456717	N	7.773315	-2.541338	2.471665	N	9.385027	-7.029072	3.274442
88	N	5.339697	0.062157	1.885589	N	5.819039	0.065161	4.021096	N	12.689096	-8.147655	2.323115
89	N	7.321146	-0.938295	1.448913	N	7.847650	-0.907646	4.292758	N	11.602613	-6.186907	2.692025
90	O	9.157865	-1.238369	2.910351	O	8.608916	-2.459858	5.882941	O	10.733740	-4.584510	1.201350
91	O	10.765525	-0.517923	0.181224	O	11.192609	-0.401055	5.744113	O	12.199010	-2.631011	3.426631
92	O	11.479449	1.203323	1.646599	O	10.655538	-0.416678	8.064829	O	13.227068	-1.961583	1.384029
93	O	8.311014	0.727879	4.457897	O	6.930937	-2.057082	7.959738	O	12.115947	-5.056334	-1.049127
94	O	2.722810	-1.450304	1.100511	O	3.993030	-0.987714	1.696896	O	11.038261	-10.813379	2.670149
95	B	12.006604	0.099713	0.828247	B	11.776292	-0.654090	7.152542	B	12.485450	-1.412936	2.523866
96	C	12.841707	-2.638542	-0.150327	C	13.505733	-2.124915	5.018860	C	10.065831	-0.797326	4.371858
97	C	13.578292	-1.629063	0.772462	C	13.574965	-2.025068	6.569739	C	10.774677	0.005154	3.242340
98	C	9.534008	-5.348558	-0.305608	C	11.319373	-4.290953	2.009893	C	7.182755	-4.019194	4.613960
99	C	14.035842	-0.537435	-0.255566	C	14.088011	-0.546870	6.802690	C	12.136059	0.429095	3.929338
100	C	14.013520	-1.223866	-1.642883	C	14.414474	-0.002058	5.387968	C	11.989038	0.005555	5.414718
101	C	11.655055	-4.813596	0.184061	C	13.010481	-4.025266	3.457531	C	7.786023	-1.858377	4.493719
102	C	15.381730	-1.340485	-2.312098	C	15.776348	0.674119	5.284480	C	12.368450	1.099478	6.402950

103	C	12.051213	-6.114431	0.496077	C	13.721304	-5.152098	3.037038	C	6.500203	-1.363402	4.710694
104	C	11.099579	-7.189717	0.418294	C	13.196118	-5.980185	1.989057	C	5.412213	-2.267284	4.896691
105	C	13.813656	-4.875766	0.679303	C	14.925166	-4.210615	4.564170	C	7.798178	0.363344	4.568733
106	H	14.814846	-4.515375	0.836677	H	15.706984	-3.954059	5.256961	H	8.193479	1.362922	4.567097
107	H	11.818176	-2.286617	-0.306283	H	12.511389	-1.800016	4.709947	H	10.382240	-1.839064	4.287008
108	H	9.065185	-7.339905	-0.071449	H	11.453077	-5.989147	0.835774	H	5.142802	-4.320119	4.895562
109	H	8.129154	-5.064111	-1.754177	H	10.194304	-3.647387	0.424709	H	7.121952	-5.901772	5.424379
110	H	7.899625	-4.135505	-0.357300	H	9.560449	-3.251159	1.964262	H	8.319038	-5.688378	4.195956
111	H	14.414308	-2.103066	1.296996	H	14.245815	-2.784366	6.986037	H	10.167680	0.860800	2.926275
112	H	15.034030	-0.146717	-0.027604	H	14.981842	-0.511234	7.432615	H	12.316020	1.505460	3.865061
113	H	13.273302	-0.713196	-2.263908	H	13.579340	0.623047	5.062082	H	12.524357	-0.936727	5.558100
114	H	15.258545	-1.743565	-3.329693	H	15.903546	1.103805	4.278760	H	12.248500	0.730311	7.432757
115	H	15.858111	-0.358287	-2.374115	H	15.852620	1.472191	6.027539	H	13.407554	1.400134	6.246405
116	H	15.582675	-2.929188	-1.251436	H	16.446221	-1.122175	5.100303	H	10.624079	1.852312	6.039458
117	N	9.821312	-6.661317	-0.020869	N	11.936916	-5.425011	1.530322	N	5.876132	-3.617144	4.829166
118	N	8.200519	-5.064348	-0.726743	N	10.100911	-3.937453	1.404059	N	7.404613	-5.388689	4.587054
119	N	10.419015	-4.388741	-0.206198	N	11.819986	-3.560076	2.978214	N	8.169144	-3.164107	4.436386
120	N	13.412452	-6.134399	0.813574	N	14.920401	-5.256489	3.748497	N	6.522020	0.032296	4.745750
121	N	12.773532	-4.020185	0.301494	N	13.774166	-3.426143	4.433412	N	8.609786	-0.756463	4.400290
122	O	13.615452	-2.646625	-1.406782	O	14.540430	-1.205651	4.513821	O	10.524088	-0.191647	5.630421
123	O	12.682357	-0.966169	1.666423	O	12.276224	-2.082855	7.169954	O	11.141479	-0.825299	2.136878
124	O	13.024628	0.490708	-0.166772	O	12.980882	0.157965	7.398602	O	13.160700	-0.330303	3.257943
125	O	16.226126	-2.196322	-1.486150	O	16.803788	-0.320247	5.584572	O	11.529878	2.265888	6.140860
126	O	11.210923	-8.398849	0.645789	O	13.627555	-7.012709	1.464336	O	4.203683	-2.050021	5.091824

S2.1.2. Aγ1-G2

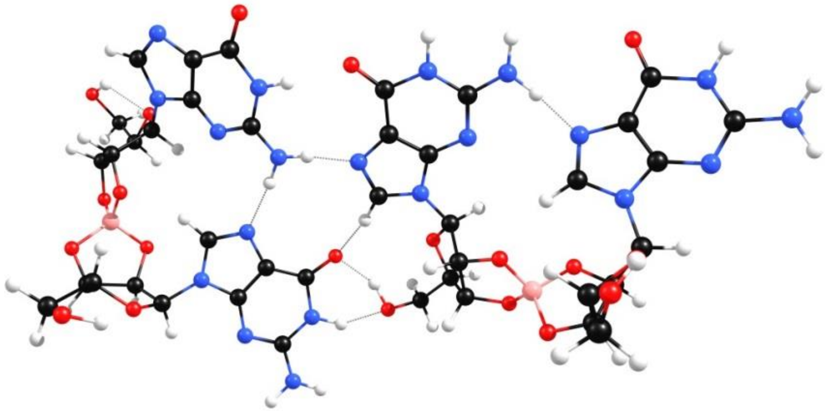

Figure S10. Optimized geometry of **Aγ1-G21**, formed by two borate-guanosine diesters with one α-guanosine and one β-guanosine in anti conformation.

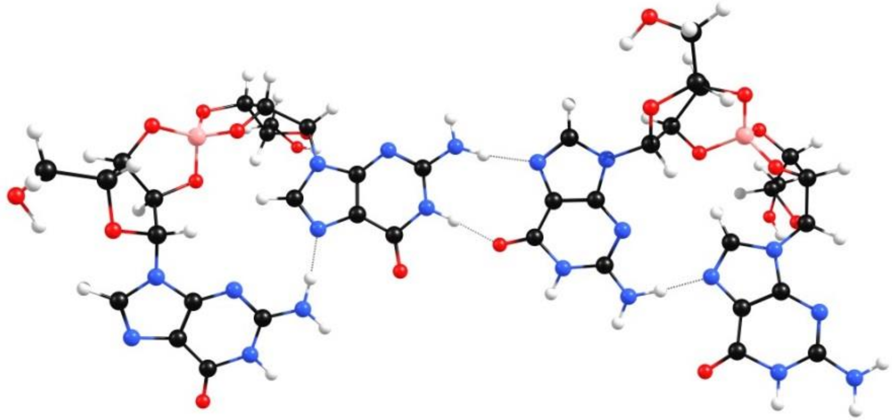

Figure S11. Optimized geometry of **Aγ1-G22**, formed by two borate-guanosine diesters with one α-guanosine and one β-guanosine in anti conformation.

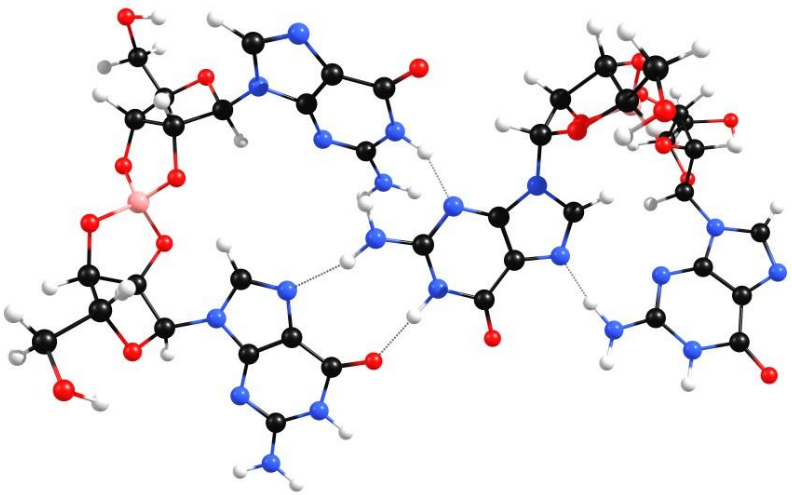

Figure S12. Optimized geometry of **Aγ1-G2$_3$**, formed by two borate-guanosine diesters with one α-guanosine and one β-guanosine in anti conformation.

Table S8. Optimized xyz cartesian coordinates for **Aγ1-G2** structures.

	**Aγ1-G2$_1$**				**Aγ1-G2$_2$**				**Aγ1-G2$_3$**			
**Number**	**Atom**	**x**	**y**	**z**	**Atom**	**x**	**y**	**z**	**Atom**	**x**	**y**	**z**
1	C	1.468525	-11.047129	-1.319970	C	2.200409	-11.319087	-1.383659	C	0.173694	-8.316847	-0.946090
2	C	0.166635	-10.962444	-2.173874	C	0.820558	-11.358901	-2.121150	C	-1.005038	-9.154277	-1.531649
3	C	5.731849	-10.949398	-1.513052	C	6.319799	-10.297665	-1.036953	C	3.117293	-5.296484	-0.794763
4	C	-0.990513	-11.263684	-1.150240	C	-0.191231	-11.910380	-1.038717	C	-1.485925	-10.061592	-0.326852
5	C	-0.366647	-10.899236	0.207059	C	0.574315	-11.781897	0.287490	C	-0.775271	-9.470832	0.902110
6	C	3.711011	-9.973143	-1.411543	C	4.130369	-9.792936	-1.057046	C	0.981943	-5.997158	-0.729965
7	C	-0.825564	-11.725591	1.399744	C	0.342874	-12.872582	1.323079	C	-0.272481	-10.461805	1.943465
8	C	4.206790	-8.688152	-1.226209	C	4.317547	-8.465951	-0.697278	C	0.455975	-4.756053	-0.386188
9	C	5.610773	-8.456925	-1.149694	C	5.621504	-7.951979	-0.447481	C	1.329342	-3.652813	-0.197595
10	C	2.028897	-8.505408	-1.213269	C	2.159148	-8.730596	-0.915632	C	-1.244912	-6.118399	-0.517735

11	H	1.014568	-8.133836	-1.169725	H	1.092557	-8.558863	-0.961512	H	-2.238110	-6.545238	-0.538578
12	H	2.103316	-11.883444	-1.616395	H	2.976407	-11.889029	-1.894378	H	1.090047	-8.391803	-1.530400
13	H	7.331006	-9.616014	-1.281834	H	7.560301	-8.700013	-0.488543	H	3.351730	-3.266326	-0.309524
14	H	7.561230	-11.967780	-1.611546	H	8.333213	-10.877446	-1.083229	H	5.134736	-4.748582	-0.896663
15	H	6.110702	-12.932470	-1.779290	H	7.147566	-12.107790	-1.461267	H	4.752408	-6.422271	-1.236573
16	H	0.214193	-11.708708	-2.979142	H	0.906447	-12.045742	-2.974598	H	-0.612193	-9.767292	-2.354797
17	H	-1.298414	-12.317415	-1.165766	H	-0.445832	-12.962127	-1.228180	H	-1.171094	-11.105314	-0.472247
18	H	-0.467448	-9.823245	0.377191	H	0.422148	-10.782731	0.707389	H	-1.394451	-8.686809	1.348346
19	H	-1.821329	-11.408446	1.718447	H	-0.630072	-12.741805	1.802470	H	-1.104775	-10.849641	2.535694
20	H	-0.869708	-12.782090	1.083773	H	0.358553	-13.844209	0.801908	H	0.205932	-11.298310	1.407067
21	H	0.983189	-11.520263	2.074150	H	2.197779	-12.607538	1.835070	H	1.191948	-9.220114	2.240860
22	N	6.319301	-9.714176	-1.324873	N	6.602738	-8.997924	-0.660548	N	2.692877	-4.032096	-0.436873
23	N	6.554950	-12.032612	-1.654287	N	7.369672	-11.157351	-1.195296	N	4.453134	-5.487729	-0.990538
24	N	4.425736	-11.124932	-1.559940	N	5.092840	-10.738650	-1.244546	N	2.287390	-6.311031	-0.949467
25	N	3.145342	-7.791210	-1.098084	N	3.084295	-7.825251	-0.611639	N	-0.933726	-4.849538	-0.254866
26	N	2.334869	-9.865580	-1.407533	N	2.765756	-9.972838	-1.198978	N	-0.083664	-6.868702	-0.815829
27	O	1.069615	-11.282237	0.062502	O	2.001601	-11.976816	-0.097590	O	0.486305	-8.894334	0.353801
28	O	-0.129130	-9.659725	-2.679658	O	0.303108	-10.092968	-2.507160	O	-2.143242	-8.398537	-1.921326
29	O	-2.059359	-10.394119	-1.512906	O	-1.337373	-11.068138	-1.098969	O	-2.899192	-9.927681	-0.269191
30	O	0.088835	-11.537661	2.520670	O	1.367793	-12.797622	2.358788	O	0.655177	-9.799828	2.853183
31	O	6.263583	-7.422097	-0.957830	O	5.995530	-6.825632	-0.090480	O	1.107148	-2.472874	0.135587
32	B	-1.464065	-9.186648	-2.167427	B	-1.068445	-9.854622	-1.919600	B	-3.390177	-8.903846	-1.247272
33	C	-1.389299	-5.734167	-2.096860	C	-1.392560	-6.369463	-2.201631	C	-5.990002	-6.565335	-1.676444
34	C	-2.109930	-6.965767	-1.476588	C	-2.005613	-7.648175	-1.556068	C	-5.484832	-7.785425	-0.858768
35	C	2.233179	-4.599363	0.072766	C	1.793276	-4.630841	0.085500	C	-4.826660	-2.480798	-1.551833
36	C	-3.036777	-7.464341	-2.636442	C	-2.747840	-8.346673	-2.753952	C	-5.706734	-8.998467	-1.831997
37	C	-3.064287	-6.322103	-3.673985	C	-2.488034	-7.431332	-3.973675	C	-6.433442	-8.407514	-3.062570
38	C	0.078065	-4.382571	-0.528320	C	-0.309463	-4.681283	-0.708248	C	-6.128387	-4.190782	-0.954945
39	C	-4.461587	-5.797621	-3.994127	C	-3.705092	-7.211109	-4.861987	C	-7.655094	-9.203374	-3.509856
40	C	-0.132380	-3.211226	0.200335	C	-0.698780	-3.459484	-0.157635	C	-6.830313	-3.495877	0.033753

41	C	0.920208	-2.630818	0.952186	C	0.249583	-2.702122	0.581088	C	-6.465141	-2.131935	0.313403
42	C	-2.005377	-3.623056	-0.817778	C	-2.426224	-4.215034	-1.234714	C	-7.575476	-5.532193	0.085146
43	H	-2.998458	-3.522407	-1.213874	H	-3.390136	-4.334689	-1.691986	H	-8.111754	-6.436136	0.315422
44	H	-0.440118	-6.063417	-2.530617	H	-0.347427	-6.587886	-2.441299	H	-5.145740	-6.136083	-2.223082
45	H	2.908358	-3.072407	1.344466	H	2.214953	-2.892259	1.183408	H	-4.788512	-0.947019	-0.151045
46	H	4.232174	-4.916727	0.527981	H	3.765933	-4.740475	0.730042	H	-3.580351	-0.935168	-2.145466
47	H	3.409338	-6.166597	-0.467058	H	3.091869	-6.175436	-0.115540	H	-3.387007	-2.460837	-2.992070
48	H	-2.677195	-6.647395	-0.595789	H	-2.685057	-7.363056	-0.744365	H	-6.066167	-7.886885	0.065312
49	H	-4.047698	-7.682102	-2.275468	H	-3.828284	-8.407847	-2.584000	H	-6.339323	-9.766100	-1.370074
50	H	-2.559506	-6.687882	-4.575023	H	-1.622391	-7.816728	-4.521915	H	-5.693890	-8.260469	-3.856291
51	H	-4.377310	-4.920734	-4.651823	H	-3.427732	-6.577878	-5.718239	H	-8.078593	-8.746820	-4.417722
52	H	-5.029841	-6.579770	-4.506448	H	-4.072023	-8.172709	-5.229329	H	-7.371634	-10.237261	-3.724890
53	H	-4.715277	-4.660374	-2.351326	H	-4.280780	-5.901940	-3.555736	H	-8.563783	-8.274330	-2.084707
54	N	2.111209	-3.430842	0.823581	N	1.503344	-3.393266	0.655383	N	-5.365000	-1.738589	-0.528638
55	N	3.412007	-5.245664	0.036752	N	2.997393	-5.188891	0.248745	N	-3.876875	-1.886419	-2.318912
56	N	1.210568	-5.105985	-0.620638	N	0.877456	-5.296740	-0.628536	N	-5.176145	-3.737541	-1.803982
57	N	-1.434373	-2.761161	0.007894	N	-2.031021	-3.188283	-0.493600	N	-7.747831	-4.355119	0.660961
58	N	-1.117763	-4.643863	-1.169522	N	-1.407585	-5.158520	-1.394159	N	-6.582583	-5.494508	-0.912392
59	O	-2.321582	-5.184286	-3.081651	O	-2.196040	-6.089938	-3.394045	O	-7.000562	-7.111285	-2.602267
60	O	-1.207697	-8.050245	-1.193388	O	-1.001442	-8.575248	-1.124047	O	-4.072955	-7.734121	-0.599938
61	O	-2.368626	-8.623610	-3.180256	O	-2.134370	-9.629013	-2.920109	O	-4.395623	-9.472539	-2.174950
62	O	-5.178607	-5.475738	-2.762742	O	-4.770649	-6.609130	-4.065175	O	-8.614725	-9.221484	-2.410624
63	O	0.937352	-1.593413	1.643557	O	0.144192	-1.587480	1.133018	O	-6.894579	-1.324363	1.152331
64	C	-9.290713	-0.159037	0.921510	C	-5.466061	3.880769	1.304369	C	-5.232656	2.022073	1.031965
65	C	-9.700600	1.344061	0.842783	C	-6.182301	5.091965	0.621897	C	-6.261111	2.860765	0.212150
66	C	-8.309326	-3.521449	-1.554901	C	-3.004569	0.414152	0.732421	C	-2.237684	-1.096058	0.465219
67	C	-10.048774	1.732767	2.325892	C	-6.957719	5.804924	1.791444	C	-7.037260	3.698256	1.289546
68	C	-9.239755	0.715620	3.150645	C	-6.204369	5.338735	3.046901	C	-6.038991	3.780361	2.458327
69	C	-7.818395	-1.614236	-0.470294	C	-3.340290	2.622727	1.001946	C	-2.975390	1.057446	0.441819
70	C	-9.867934	0.282450	4.466867	C	-7.029675	5.189150	4.317320	C	-6.645476	3.780274	3.854735

71	C	-6.446108	-1.690574	-0.721914	C	-1.992076	2.925232	1.106975	C	-1.713883	1.594379	0.238246
72	C	-5.948269	-2.799141	-1.447835	C	-1.011197	1.886094	1.073424	C	-0.568758	0.743888	0.146611
73	C	-6.740166	0.112295	0.468092	C	-3.049050	4.832509	1.294501	C	-3.085723	3.291606	0.368025
74	H	-6.606533	1.025830	1.027203	H	-3.299963	5.876229	1.396827	H	-3.520588	4.279209	0.374144
75	H	-10.017630	-0.808123	0.429669	H	-5.830927	2.918885	0.941381	H	-5.504514	0.963573	1.044802
76	H	-6.605755	-4.522064	-2.366599	H	-1.011096	-0.207306	0.920198	H	-0.181089	-1.322474	0.257433
77	H	-8.856543	-5.326200	-2.442206	H	-2.869156	-1.641413	0.208613	H	-1.800661	-3.178111	0.323173
78	H	-10.148476	-4.375999	-1.735804	H	-4.460300	-0.922985	0.425829	H	-3.407022	-2.701308	0.794502
79	H	-10.563577	1.445456	0.169487	H	-6.864359	4.709387	-0.150281	H	-6.916175	2.181101	-0.347074
80	H	-11.121459	1.646370	2.543591	H	-8.010628	5.497064	1.839733	H	-7.974685	3.222916	1.601000
81	H	-8.214452	1.076928	3.271573	H	-5.321459	5.967542	3.204007	H	-5.364223	4.626077	2.298995
82	H	-9.755284	1.070282	5.216014	H	-7.245097	6.170419	4.748045	H	-7.082000	4.757195	4.078465
83	H	-10.943607	0.110478	4.287477	H	-7.980588	4.698399	4.048447	H	-7.438879	3.015301	3.883897
84	H	-9.050072	-1.449392	4.141166	H	-5.849133	3.709867	4.744826	H	-5.074918	2.788643	4.411281
85	N	-6.979991	-3.688798	-1.856494	N	-1.644685	0.610284	0.875469	N	-0.956959	-0.637985	0.277734
86	N	-9.177736	-4.475980	-2.000446	N	-3.453461	-0.844911	0.513186	N	-2.451626	-2.423337	0.597276
87	N	-8.778015	-2.492779	-0.862531	N	-3.899748	1.406571	0.806391	N	-3.294700	-0.260802	0.513798
88	N	-5.792342	-0.606431	-0.121236	N	-1.832080	4.301987	1.289885	N	-1.803367	2.988762	0.197461
89	N	-8.011260	-0.472637	0.278905	N	-4.020807	3.830637	1.120642	N	-3.853359	2.123877	0.528992
90	O	-9.270129	-0.531296	2.332953	O	-5.814150	3.940326	2.729943	O	-5.289078	2.494972	2.413106
91	O	-8.641771	2.233016	0.484862	O	-5.311648	6.100354	0.106717	O	-5.672474	3.849940	-0.644783
92	O	-9.579524	3.069065	2.495925	O	-6.828038	7.206289	1.555799	O	-7.267736	4.978321	0.691065
93	O	-9.210811	-0.917525	4.972215	O	-6.285014	4.414959	5.304935	O	-5.607586	3.515044	4.846196
94	O	-4.759248	-3.100532	-1.751691	O	0.222136	1.971602	1.207266	O	0.626408	1.047321	-0.001253
95	B	-8.414997	3.254520	1.573973	B	-5.620064	7.443041	0.717112	B	-6.162639	5.218897	-0.277339
96	C	-4.970031	4.076287	1.576823	C	-2.556179	9.116650	0.417663	C	-3.574829	7.496744	-1.019287
97	C	-6.305138	4.145267	2.368403	C	-3.955274	9.170160	1.094364	C	-4.895950	7.291389	-0.226152
98	C	-2.569370	0.595659	1.319721	C	0.561629	6.713538	2.039781	C	0.084765	5.911473	0.483227
99	C	-7.121647	5.268827	1.635672	C	-4.917300	9.607094	-0.068634	C	-6.005040	7.364994	-1.339723
100	C	-6.200297	5.750339	0.488889	C	-4.002368	9.781911	-1.305534	C	-5.246678	7.702945	-2.647080

101	C	-3.030202	2.643449	2.126531	C	-0.449154	8.688273	1.671738	C	-1.343007	7.600550	0.081658
102	C	-6.164663	7.266618	0.320720	C	-4.288309	11.040979	-2.118834	C	-5.887616	8.810439	-3.476013
103	C	-1.842729	2.801781	2.840452	C	0.523512	9.487578	2.282823	C	-0.523212	8.625309	0.567824
104	C	-0.864722	1.749498	2.789743	C	1.677554	8.856189	2.842907	C	0.771106	8.289621	1.073161
105	C	-2.972543	4.636586	3.136014	C	-1.001751	10.846437	1.552725	C	-2.345144	9.583364	-0.114230
106	H	-3.299822	5.620886	3.422562	H	-1.578826	11.714618	1.287822	H	-3.108280	10.291646	-0.382997
107	H	-5.072321	3.275383	0.838686	H	-2.366867	8.079266	0.127851	H	-3.190065	6.507819	-1.284148
108	H	-0.666451	-0.118510	1.870658	H	2.380771	6.915693	3.053736	H	1.879212	6.557860	1.345057
109	H	-2.296216	-1.309562	0.483087	H	1.361207	4.818791	2.312297	H	1.282175	4.258356	0.833337
110	H	-3.873543	-0.487321	0.243638	H	-0.300236	4.913046	1.617797	H	-0.392366	3.940148	0.257554
111	H	-6.111098	4.384319	3.420550	H	-3.940807	9.891189	1.921255	H	-5.005902	8.086218	0.521949
112	H	-7.329037	6.116447	2.299533	H	-5.405582	10.564709	0.148709	H	-6.730837	8.160355	-1.130410
113	H	-6.480708	5.220022	-0.427075	H	-4.046007	8.863845	-1.899847	H	-5.098234	6.771767	-3.202406
114	H	-5.549378	7.528608	-0.554513	H	-3.648996	11.055719	-3.015213	H	-5.319546	8.950619	-4.408809
115	H	-7.179268	7.647280	0.173317	H	-5.337842	11.054137	-2.425023	H	-6.919506	8.543689	-3.719286
116	H	-4.904366	7.202911	1.775828	H	-3.223217	11.919718	-0.776264	H	-5.018599	9.999455	-2.231303
117	N	-1.336451	0.657584	1.964104	N	1.591394	7.418664	2.655315	N	0.978572	6.855413	0.977421
118	N	-2.911932	-0.484205	0.622680	N	0.595899	5.378872	1.959592	N	0.403265	4.609597	0.475631
119	N	-3.448713	1.614501	1.384441	N	-0.490763	7.356686	1.520908	N	-1.109340	6.280267	0.010565
120	N	-1.829902	4.062523	3.471939	N	0.153357	10.842986	2.197075	N	-1.175235	9.864840	0.435428
121	N	-3.757903	3.808814	2.308241	N	-1.422026	9.550047	1.206682	N	-2.503315	8.207172	-0.352452
122	O	-4.827501	5.397262	0.923580	O	-2.638158	10.005918	-0.755972	O	-3.945706	8.281466	-2.209270
123	O	-7.080146	2.939782	2.214371	O	-4.401396	7.872622	1.509925	O	-4.987157	5.978012	0.338025
124	O	-8.315465	4.654591	1.127125	O	-5.850476	8.537407	-0.252555	O	-6.599071	6.062702	-1.408036
125	O	-5.635091	7.852077	1.548964	O	-4.051459	12.202342	-1.265477	O	-5.917076	10.030254	-2.673340
126	O	0.253735	1.677940	3.322963	O	2.672526	9.320107	3.422885	O	1.680712	8.989628	1.545728

S2.1.3. Aα$_2$-G2

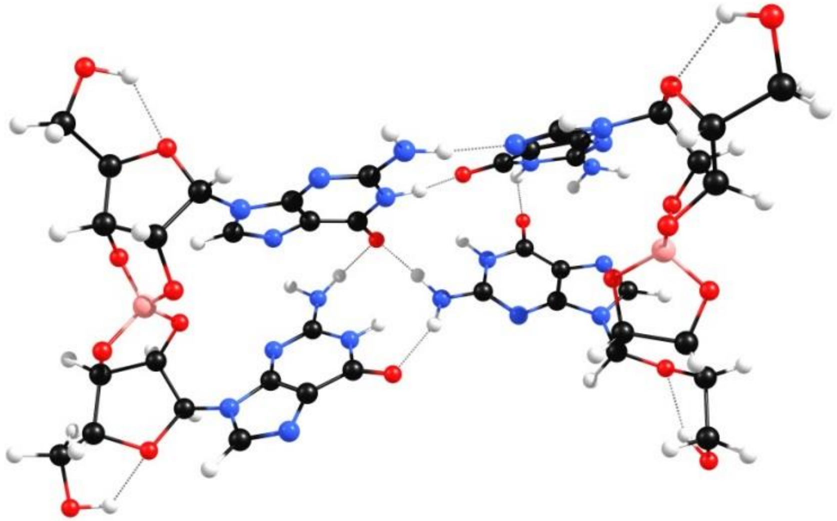

Figure S13. Optimized geometry of **Aα$_2$-G2$_1$**, formed by two borate-guanosine diesters with two α-guanosines in anti conformation.

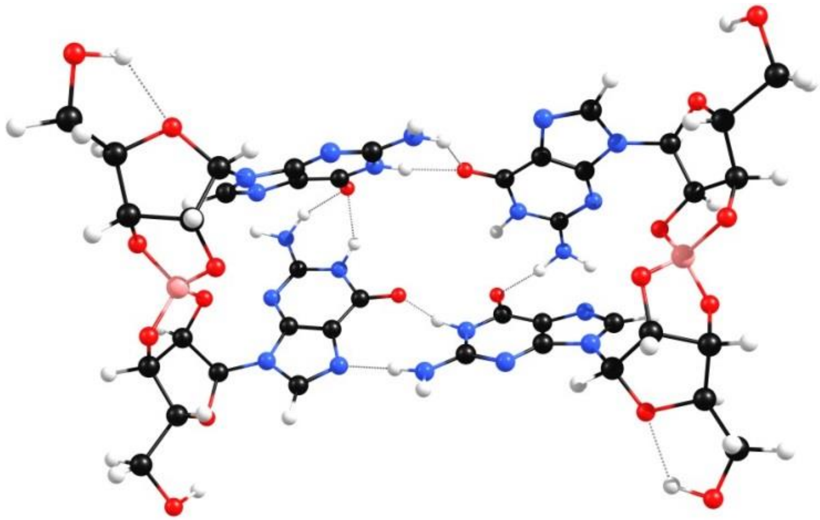

Figure S14. Optimized geometry of **Aα$_2$-G2$_2$**, formed by two borate-guanosine diesters with two α-guanosines in anti conformation.

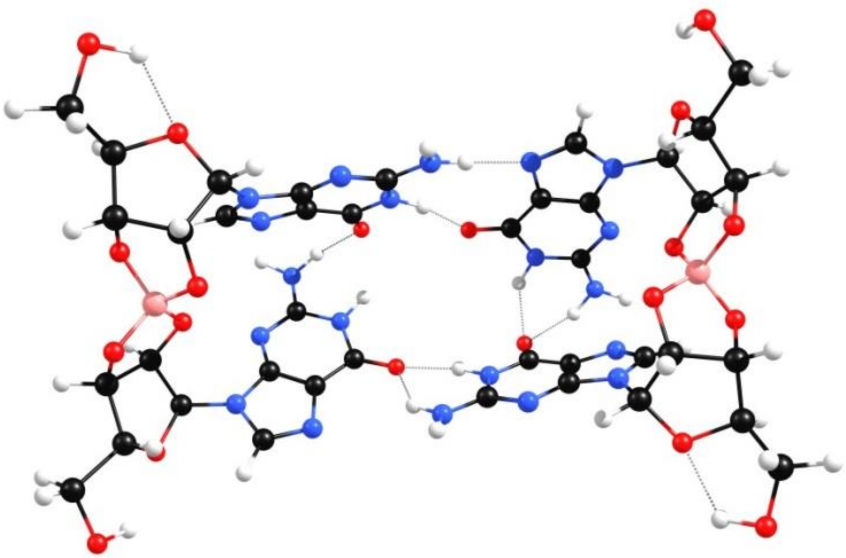

Figure S15. Optimized geometry of **Aα2-G23**, formed by two borate-guanosine diesters with two α-guanosines in anti conformation.

Table S9. Optimized xyz cartesian coordinates for **Aα2-G2** structures.

	Aα2-G21				Aα2-G22				Aα2-G23			
**Number**	**Atom**	**x**	**y**	**z**	**Atom**	**x**	**y**	**z**	**Atom**	**x**	**y**	**z**
1	B	-2.705803	-4.810019	-7.932359	B	-0.843112	-3.540482	-1.430900	B	-0.731251	-3.195250	-0.704090
2	O	-1.824010	-4.321950	-6.807605	O	-0.187023	-2.612320	-2.441374	O	-0.089875	-2.219366	-1.677618
3	O	-4.027412	-4.167794	-7.636349	O	-0.132035	-4.831322	-1.622727	O	0.098202	-4.423746	-0.839807
4	C	-4.288028	-3.281069	-5.288739	C	0.949582	-5.112986	-3.891894	C	1.326805	-4.619696	-3.044085
5	C	-3.800213	-3.066097	-6.766322	C	1.039911	-4.615567	-2.405932	C	1.289123	-4.118444	-1.559500
6	C	-7.368522	-6.099996	-6.504825	C	-0.994818	-8.672662	-5.362101	C	-0.313231	-8.226413	-4.752837
7	C	-2.245920	-2.960894	-6.608139	C	1.181270	-3.067919	-2.547528	C	1.314801	-2.563448	-1.703031
8	C	-1.998854	-2.475343	-5.194685	C	1.787387	-2.830430	-3.921527	C	1.986394	-2.282223	-3.040726
9	C	-5.557512	-5.466537	-5.361870	C	-0.894446	-6.477661	-4.894712	C	-0.364505	-6.045286	-4.219932

10	C	-1.848870	-0.956448	-5.081686	C	3.300606	-2.589599	-3.888304	C	3.458301	-1.875477	-2.912725
11	C	-5.358118	-6.718624	-4.761633	C	-2.193362	-6.200306	-5.339979	C	-1.634565	-5.818095	-4.766693
12	C	-6.264645	-7.769152	-5.044055	C	-3.005287	-7.275543	-5.782702	C	-2.351502	-6.918232	-5.303526
13	C	-3.694444	-5.480967	-4.134901	C	-1.415304	-4.328182	-4.558017	C	-1.019682	-3.933407	-3.877229
14	H	-2.795614	-5.095711	-3.696707	H	-1.283383	-3.340004	-4.152529	H	-0.972491	-2.952358	-3.436878
15	H	-5.218643	-2.742495	-5.091599	H	1.438271	-6.077727	-4.038727	H	1.865924	-5.561484	-3.158474
16	H	-7.877345	-8.087195	-6.306135	H	-2.867570	-9.372163	-5.891795	H	-2.097750	-8.999675	-5.457710
17	H	-8.141343	-5.218586	-8.130475	H	0.445064	-10.070560	-5.102542	H	1.171720	-9.563160	-4.419861
18	H	-4.250414	-2.138635	-7.141628	H	1.925560	-5.072987	-1.949702	H	2.183611	-4.500428	-1.053167
19	H	-1.822606	-2.272538	-7.357248	H	1.815209	-2.651150	-1.749651	H	1.862756	-2.096060	-0.870369
20	H	-1.175126	-3.007672	-4.717392	H	1.260901	-2.042680	-4.463974	H	1.415477	-1.567062	-3.636531
21	H	-0.842213	-0.649435	-5.379902	H	3.522404	-1.560911	-3.587714	H	3.544544	-0.821216	-2.633155
22	H	-2.573772	-0.483018	-5.766119	H	3.740708	-3.274347	-3.142858	H	3.915387	-2.487903	-2.116453
23	H	-2.804772	-1.147263	-3.415168	H	3.331478	-3.599545	-5.529421	H	3.714802	-2.920401	-4.510298
24	N	-7.302675	-7.337513	-5.885412	N	-2.324068	-8.508823	-5.739968	N	-1.613287	-8.116202	-5.235549
25	N	-8.298900	-5.994792	-7.493730	N	-0.490569	-9.914675	-5.454558	N	0.259299	-9.438708	-4.838733
26	N	-6.554590	-5.096597	-6.194956	N	-0.242877	-7.656347	-4.925190	N	0.346934	-7.189191	-4.226066
27	N	-4.186785	-6.705056	-3.997634	N	-2.486145	-4.834507	-5.139115	N	-2.010450	-4.474321	-4.559171
28	N	-4.504781	-4.687383	-4.944550	N	-0.402531	-5.291051	-4.391160	N	0.023653	-4.850990	-3.646795
29	O	-3.246971	-2.776755	-4.403928	O	1.657634	-4.109152	-4.691456	O	2.054743	-3.584566	-3.780834
30	O	-2.056180	-0.544400	-3.701085	O	3.854921	-2.802702	-5.216010	O	4.125165	-2.061946	-4.191798
31	O	-6.240641	-8.982210	-4.696091	O	-4.230584	-7.253357	-6.135225	O	-3.541657	-6.947419	-5.760067
32	H	-8.552277	-6.889909	-7.936158	H	-1.124981	-10.700498	-5.665017	H	-0.313010	-10.245265	-5.128235
33	C	-2.795712	-7.373702	-10.307723	C	-4.192403	-3.486275	0.026045	C	-4.182784	-3.459906	0.527586
34	C	-1.954841	-6.705332	-9.155888	C	-2.997262	-2.798296	-0.725473	C	-2.983045	-2.673755	-0.113150
35	C	-4.754437	-10.218296	-7.737618	C	-5.227058	-5.446080	-3.718650	C	-4.822286	-5.295042	-3.384552
36	C	-1.377844	-5.391693	-9.808357	C	-1.942480	-2.463846	0.407047	C	-2.040456	-2.325369	1.106634
37	C	-1.619322	-5.521638	-11.304721	C	-2.457727	-3.179507	1.663368	C	-2.524147	-3.249856	2.231324
38	C	-4.726591	-8.502686	-9.184922	C	-4.732180	-5.402434	-1.539622	C	-4.523253	-5.321294	-1.168879
39	C	-0.442316	-6.107441	-12.091612	C	-2.413136	-2.377367	2.957368	C	-2.489608	-2.688336	3.644571

40	C	-6.097947	-8.215584	-9.079816	C	-4.763966	-6.792863	-1.385681	C	-4.543440	-6.717265	-1.067877
41	C	-6.886118	-9.000531	-8.212292	C	-5.090472	-7.606810	-2.502978	C	-4.751869	-7.496971	-2.237034
42	C	-5.256627	-6.659475	-10.323494	C	-4.152477	-5.994486	0.533152	C	-4.128484	-5.983328	0.927113
43	H	-5.078442	-5.791681	-10.928721	H	-3.846390	-5.867805	1.553486	H	-3.920187	-5.890086	1.975189
44	H	-2.396500	-8.365064	-10.541855	H	-5.138004	-2.978127	-0.179985	H	-5.137964	-2.974702	0.311584
45	H	-6.569712	-10.431509	-6.741856	H	-5.250060	-7.330261	-4.557617	H	-4.716903	-7.147397	-4.286708
46	H	-4.732183	-11.560143	-6.179981	H	-5.105714	-5.567272	-5.711732	H	-4.530165	-5.337648	-5.362697
47	H	-3.203125	-11.421462	-7.090321	H	-4.892274	-4.009196	-5.046723	H	-4.382653	-3.810712	-4.623195
48	H	-1.170756	-7.404617	-8.849249	H	-3.375460	-1.879801	-1.193347	H	-3.381730	-1.763892	-0.581277
49	H	-0.305391	-5.272950	-9.585803	H	-1.893114	-1.378035	0.581323	H	-2.155116	-1.273777	1.408532
50	H	-1.963572	-4.579030	-11.730529	H	-1.952722	-4.143921	1.754524	H	-2.005311	-4.208731	2.152056
51	H	0.326900	-5.345388	-12.248512	H	-1.387235	-2.309445	3.327412	H	-1.465071	-2.689123	4.024052
52	H	0.003380	-6.932913	-11.509832	H	-2.782088	-1.361303	2.739042	H	-2.857611	-1.649437	3.611564
53	H	-1.823192	-6.919612	-13.162222	H	-4.038976	-3.298146	3.488738	H	-4.116213	-3.693683	3.995566
54	N	-6.120984	-9.982542	-7.543574	N	-5.292987	-6.828723	-3.657773	N	-4.861373	-6.681094	-3.379028
55	N	-4.207287	-11.191067	-6.995364	N	-5.366665	-4.906723	-4.961565	N	-4.864520	-4.709157	-4.613343
56	N	-4.036293	-9.498688	-8.603081	N	-5.018496	-4.685152	-2.645598	N	-4.724831	-4.570692	-2.270470
57	N	-6.409985	-7.053093	-9.808985	N	-4.392198	-7.144767	-0.084658	N	-4.288192	-7.112413	0.248226
58	N	-4.204512	-7.524641	-9.991361	N	-4.352063	-4.904158	-0.312578	N	-4.265684	-4.863346	0.106005
59	O	-2.737371	-6.497749	-11.483878	O	-3.928010	-3.382613	1.449711	O	-3.988071	-3.432708	1.969611
60	O	-2.742681	-6.281542	-8.042732	O	-2.309704	-3.629557	-1.651797	O	-2.167876	-3.426205	-1.005069
61	O	-2.186561	-4.339794	-9.259660	O	-0.698541	-3.007313	-0.035717	O	-0.713683	-2.638308	0.691438
62	O	-0.917222	-6.553501	-13.393553	O	-3.213480	-3.032939	3.985709	O	-3.294302	-3.518611	4.536019
63	O	-8.116884	-8.854629	-7.907220	O	-5.186617	-8.854652	-2.587867	O	-4.816643	-8.742546	-2.370306
64	B	-1.045958	-16.395365	-1.395143	B	-1.118936	-16.837378	-1.702080	B	-0.273231	-16.478079	-1.525797
65	O	-1.866662	-16.568962	-0.129742	O	-1.922584	-17.567086	-0.633423	O	0.990921	-17.163628	-1.953998
66	O	-0.625672	-14.972116	-1.352767	O	-0.669010	-15.597572	-1.017539	O	-1.035269	-16.276297	-2.786657
67	C	-2.137807	-13.446439	-0.000713	C	-2.145769	-14.892479	0.922919	C	-0.309809	-16.621510	-5.184766
68	C	-0.907158	-14.423280	-0.065786	C	-0.916892	-15.705716	0.382458	C	-0.603490	-17.217155	-3.761339
69	C	-3.705793	-10.277966	-2.463556	C	-3.792599	-11.034193	-0.104814	C	-3.733835	-13.960679	-4.696969

70	C	-1.338377	-15.629005	0.829011	C	-1.327632	-17.191230	0.628710	C	0.795925	-17.750579	-3.242826
71	C	-2.404371	-15.094075	1.774130	C	-2.319798	-17.159854	1.783614	C	1.821848	-17.238064	-4.261053
72	C	-3.492299	-12.427837	-1.832620	C	-3.631298	-13.271816	-0.274161	C	-1.584834	-14.433509	-5.118620
73	C	-1.875951	-14.780230	3.179482	C	-1.697334	-17.533657	3.134370	C	2.885380	-18.233543	-4.708014
74	C	-4.362832	-12.924953	-2.813852	C	-4.633598	-13.387809	-1.244355	C	-1.209783	-13.095991	-5.303220
75	C	-4.890516	-12.017486	-3.756718	C	-5.200028	-12.198259	-1.774099	C	-2.189649	-12.080979	-5.184349
76	C	-3.691006	-14.654834	-1.686310	C	-3.904398	-15.406304	-0.896364	C	0.604158	-14.243841	-5.511454
77	H	-3.477111	-15.641824	-1.319035	H	-3.661752	-16.453318	-0.945396	H	1.614966	-14.571878	-5.661887
78	H	-1.836184	-12.398499	0.040549	H	-1.855255	-13.942010	1.373450	H	-0.969977	-17.056828	-5.939708
79	H	-5.096617	-9.952262	-4.008102	H	-4.981138	-10.135330	-1.592566	H	-4.145071	-11.946866	-4.457634
80	H	-2.807856	-8.726896	-1.581438	H	-2.727526	-9.885172	1.142906	H	-5.039870	-15.149150	-3.815441
81	H	-0.038727	-13.893019	0.342665	H	-0.040449	-15.417435	0.974907	H	-1.315399	-18.044604	-3.884223
82	H	-0.478991	-16.041064	1.380972	H	-0.450929	-17.811894	0.870207	H	0.803573	-18.851066	-3.214777
83	H	-3.275637	-15.751616	1.800897	H	-3.210243	-17.753205	1.565959	H	2.251342	-16.304692	-3.889705
84	H	-1.798379	-15.694769	3.775274	H	-1.612922	-18.620217	3.233669	H	3.636896	-18.362994	-3.925277
85	H	-0.867459	-14.342739	3.077440	H	-0.683945	-17.098601	3.176856	H	2.387996	-19.200811	-4.889377
86	H	-3.069201	-13.272013	3.097301	H	-2.840626	-16.156271	3.843471	H	2.808536	-17.384079	-6.450487
87	N	-4.562003	-10.678928	-3.472223	N	-4.720643	-11.040506	-1.128280	N	-3.446736	-12.601290	-4.831013
88	N	-3.449268	-8.962089	-2.330240	N	-3.469593	-9.850999	0.453715	N	-5.005578	-14.267918	-4.329404
89	N	-3.128590	-11.149690	-1.622242	N	-3.202000	-12.153536	0.342158	N	-2.823697	-14.912125	-4.894555
90	N	-4.482937	-14.323175	-2.687546	N	-4.803152	-14.743708	-1.597200	N	0.164646	-12.996212	-5.549447
91	N	-3.048480	-13.529845	-1.126902	N	-3.151662	-14.552786	-0.066302	N	-0.426537	-15.162987	-5.252118
92	O	-2.826005	-13.755634	1.257393	O	-2.723941	-15.730742	1.979850	O	1.054659	-16.988854	-5.524375
93	O	-2.794184	-13.874742	3.851999	O	-2.544070	-17.042755	4.210282	O	3.563817	-17.742919	-5.902665
94	O	-5.565195	-12.276086	-4.820162	O	-6.008013	-12.064312	-2.749731	O	-2.020826	-10.837436	-5.333851
95	H	-3.736122	-8.210064	-2.992125	H	-3.887382	-8.929169	0.219079	H	-5.470388	-13.498043	-3.808842
96	C	-0.944415	-18.075706	-4.487836	C	-1.486563	-17.104905	-5.347904	C	-1.669049	-14.753234	1.098969
97	C	-1.528865	-18.065146	-3.030002	C	-1.697507	-17.603286	-3.875024	C	-0.338062	-15.423190	0.606610
98	C	-3.911753	-15.151488	-5.670118	C	-4.673399	-14.190836	-4.703329	C	-3.514816	-10.977346	0.112282
99	C	-0.333792	-18.530533	-2.111720	C	-0.308650	-18.240847	-3.458860	C	-0.610970	-16.952013	0.768562

100	C	0.722837	-19.105788	-3.043529	C	0.667932	-17.790988	-4.552377	C	-1.686848	-17.070413	1.839274
101	C	-1.821563	-15.917634	-5.455722	C	-2.610242	-14.832202	-5.297772	C	-3.174611	-13.186813	-0.141652
102	C	0.716547	-20.636522	-3.119197	C	1.684862	-18.822624	-5.022528	C	-1.135016	-17.445392	3.220603
103	C	-1.204296	-14.792376	-6.028772	C	-2.172429	-13.536458	-5.605636	C	-4.062871	-13.319907	-1.215101
104	C	-2.023884	-13.731072	-6.516978	C	-3.071295	-12.451945	-5.461700	C	-4.662299	-12.146043	-1.743130
105	C	0.403037	-16.101998	-5.415048	C	-0.464466	-14.817954	-5.912193	C	-3.212313	-15.295564	-0.896340
106	H	1.350789	-16.559953	-5.209094	H	0.503442	-15.225335	-6.134251	H	-2.880621	-16.316237	-0.975169
107	H	-1.578692	-18.677011	-5.147657	H	-2.228727	-17.539622	-6.022732	H	-1.496702	-13.810726	1.621169
108	H	-4.053698	-13.346569	-6.673236	H	-4.933385	-12.141020	-4.567634	H	-4.616536	-10.082864	-1.440843
109	H	-5.624982	-14.174800	-5.258477	H	-6.280227	-13.562034	-3.679694	H	-3.770214	-8.896733	0.516289
110	H	-5.502353	-15.764500	-4.663629	H	-5.944990	-15.231832	-3.612825	H	-2.661857	-9.833580	1.515644
111	H	-2.366927	-18.771909	-2.995407	H	-2.490983	-18.362192	-3.881269	H	0.470850	-15.079053	1.262260
112	H	-0.675359	-19.288457	-1.388053	H	-0.374459	-19.339350	-3.438363	H	0.305470	-17.488082	1.060952
113	H	1.710446	-18.709012	-2.807548	H	1.139006	-16.853275	-4.247493	H	-2.494643	-17.736103	1.527950
114	H	1.210661	-21.064743	-2.241776	H	2.478829	-18.935929	-4.280411	H	-0.938621	-18.520121	3.282092
115	H	-0.332291	-20.982881	-3.128748	H	1.162880	-19.786965	-5.139298	H	-0.182869	-16.906204	3.365030
116	H	1.171372	-20.364784	-4.970223	H	1.523231	-18.040616	-6.793170	H	-2.469671	-16.231167	3.894491
117	N	-3.402595	-14.048835	-6.331064	N	-4.317150	-12.864149	-4.956025	N	-4.334585	-10.992633	-1.000128
118	N	-5.257871	-15.128382	-5.421299	N	-5.915814	-14.390937	-4.192253	N	-3.332135	-9.807680	0.755928
119	N	-3.143049	-16.157974	-5.280568	N	-3.846556	-15.211430	-4.923769	N	-2.893061	-12.076569	0.566632
120	N	0.190120	-14.931247	-5.989675	N	-0.825626	-13.547381	-5.989343	N	-4.087854	-14.661900	-1.651931
121	N	-0.795854	-16.751321	-5.076591	N	-1.519791	-15.646989	-5.494731	N	-2.620391	-14.438791	0.051513
122	O	0.391816	-18.669213	-4.438537	O	-0.176326	-17.576365	-5.771355	O	-2.246430	-15.699002	2.061030
123	O	-1.898792	-16.778082	-2.556544	O	-1.940509	-16.581756	-2.914235	O	-0.020480	-15.226089	-0.769841
124	O	0.118183	-17.327368	-1.472390	O	0.023243	-17.682305	-2.186664	O	-1.062249	-17.341078	-0.548323
125	O	1.440005	-21.067302	-4.305493	O	2.302002	-18.388192	-6.272018	O	-2.112958	-17.103148	4.241655
126	O	-1.719989	-12.630681	-7.036913	O	-2.844999	-11.232657	-5.708847	O	-5.380005	-12.018140	-2.788006

S2.2. Syn Conformation

S2.2.1. Sβ1-G2

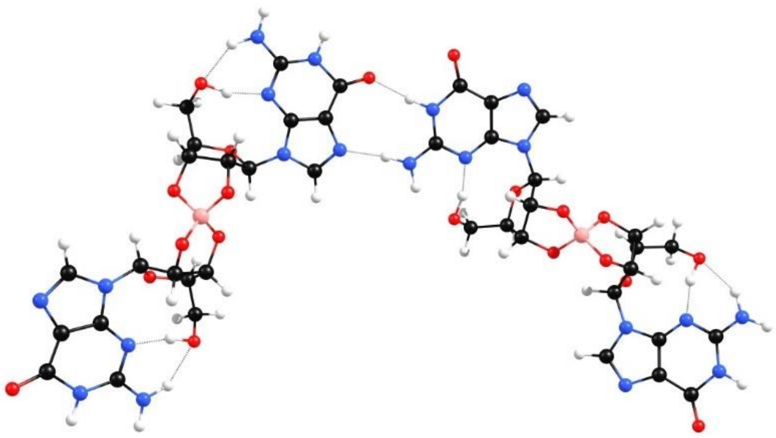

Figure S16. Optimized geometry of **Sβ1-G21**, formed by two borate-guanosine diesters with two β-guanosines in syn conformation.

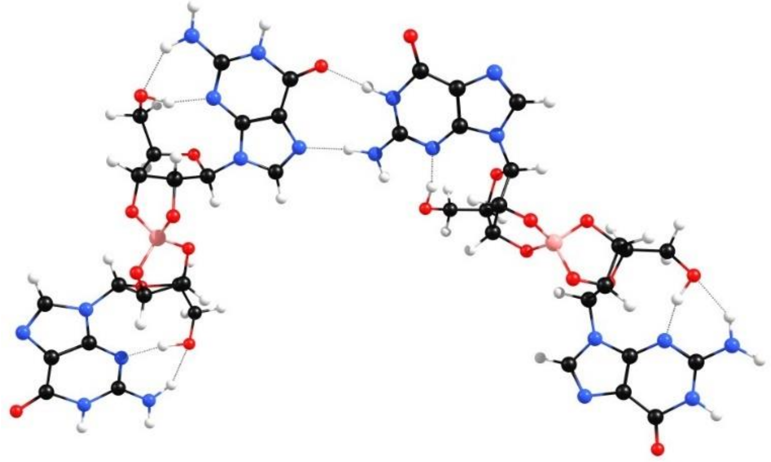

Figure S17. Optimized geometry of **Sβ1-G22**, formed by two borate-guanosine diesters with two β-guanosines in syn conformation.

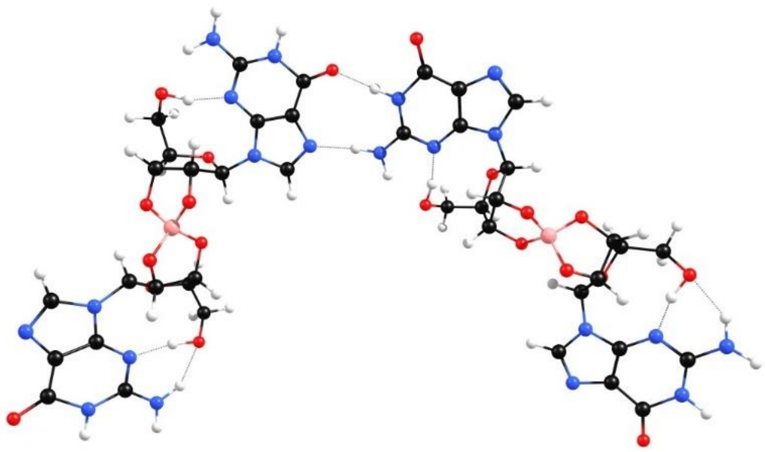

Figure S18 – Optimized geometry of **Sβ1-G23**, formed by two borate-guanosine diesters with two β-guanosines in syn conformation.

Table S10. Optimized xyz cartesian coordinates for **Sβ1-G2** structures.

	**Sβ1-G21**				**Sβ1-G22**				**Sβ1-G23**			
**Number**	**Atom**	**x**	**y**	**z**	**Atom**	**x**	**y**	**z**	**Atom**	**x**	**y**	**z**
1	O	-13.157111	8.113512	-4.862191	O	-13.428846	8.048350	-4.804030	O	-9.350222	-8.704210	-17.788740
2	O	-15.466898	7.782450	-4.449323	O	-15.704752	8.035535	-4.145755	O	-10.321473	-9.036761	-15.650406
3	B	-14.138099	7.566044	-3.845231	B	-14.354807	7.688989	-3.658729	B	-9.788796	-9.770702	-16.812081
4	C	-11.454118	6.591312	-2.991829	C	-11.738076	6.463184	-2.992955	C	-9.377100	-11.180248	-19.408870
5	C	-12.875032	6.224826	-2.449409	C	-13.129778	6.279633	-2.300758	C	-9.997219	-11.705866	-18.070785
6	C	-9.715205	4.181458	0.145634	C	-9.997620	3.937359	0.047924	C	-9.680600	-15.158004	-21.023417
7	C	-13.247058	7.484806	-1.601540	C	-13.273171	7.611400	-1.498717	C	-8.734281	-11.912698	-17.173703
8	C	-11.906056	8.123094	-1.189708	C	-11.834196	8.088901	-1.221407	C	-7.564987	-12.148486	-18.151204
9	C	-10.056580	4.727833	-2.032424	C	-10.471006	4.475975	-2.106580	C	-10.136568	-12.933189	-21.048143
10	C	-11.525763	7.904318	0.281062	C	-11.355174	7.849051	0.217600	C	-7.108661	-13.610925	-18.252774
11	C	-9.191802	3.760871	-2.537407	C	-9.755935	3.416530	-2.657306	C	-10.808626	-12.943394	-22.272629

12	C	-8.508661	2.891685	-1.641007	C	-9.091993	2.494391	-1.800331	C	-10.962204	-14.177020	-22.979829
13	C	-9.969605	4.858655	-4.239211	C	-10.561719	4.561359	-4.315003	C	-10.759202	-10.885670	-21.600192
14	H	-10.193453	5.217700	-5.227880	H	-10.831258	4.925943	-5.290085	H	-10.875030	-9.820439	-21.500692
15	H	-11.563151	7.050605	-3.978793	H	-11.891858	6.905022	-3.982210	H	-9.378033	-10.086271	-19.384724
16	H	-8.345272	2.618552	0.410333	H	-8.782345	2.241281	0.237404	H	-10.365258	-16.161857	-22.722226
17	H	-9.580410	3.890050	2.209181	H	-9.719800	3.671573	2.101319	H	-9.125417	-17.155806	-20.759983
18	H	-10.587904	5.244930	1.616254	H	-10.618230	5.119162	1.555019	H	-8.565227	-15.959010	-19.546174
19	H	-12.847702	5.299184	-1.870290	H	-13.143950	5.385057	-1.674814	H	-10.570491	-12.620615	-18.239442
20	H	-13.841064	7.222044	-0.718853	H	-13.824448	7.471416	-0.561868	H	-8.850754	-12.765685	-16.494189
21	H	-11.944098	9.191399	-1.423470	H	-11.760826	9.148784	-1.482701	H	-6.730796	-11.505181	-17.853713
22	H	-10.466010	8.180129	0.396552	H	-10.263339	7.989489	0.233935	H	-6.481214	-13.704030	-19.152602
23	H	-12.130626	8.570270	0.906170	H	-11.814759	8.595412	0.874631	H	-6.502148	-13.855726	-17.373751
24	H	-11.385463	5.939609	-0.007386	H	-11.473116	5.874335	-0.019660	H	-8.880253	-14.155662	-18.978269
25	N	-8.837241	3.202516	-0.263080	N	-9.266061	2.862956	-0.407424	N	-10.318928	-15.270290	-22.234600
26	N	-9.952291	4.444552	1.451723	N	-10.088908	4.251182	1.361365	N	-9.067093	-16.207570	-20.419343
27	N	-10.362478	4.933129	-0.737081	N	-10.638551	4.736606	-0.796342	N	-9.616499	-13.993464	-20.393742
28	N	-9.152666	3.856472	-3.932656	N	-9.826118	3.486623	-4.052474	N	-11.200320	-11.635404	-22.601099
29	N	-10.554215	5.423933	-3.101401	N	-10.987453	5.200525	-3.146908	N	-10.101141	-11.637751	-20.612340
30	O	-10.841246	7.487672	-2.020061	O	-10.933514	7.307981	-2.119218	O	-8.030098	-11.735122	-19.506227
31	O	-13.834866	6.155925	-3.508926	O	-14.192460	6.271718	-3.259001	O	-10.780329	-10.699123	-17.427023
32	O	-13.977249	8.309759	-2.535472	O	-13.962416	8.478750	-2.427300	O	-8.610728	-10.657226	-16.474692
33	O	-11.794127	6.537813	0.725608	O	-11.751171	6.534590	0.720866	O	-8.235407	-14.545075	-18.269159
34	O	-7.707890	1.966379	-1.855639	O	-8.414794	1.485058	-2.058364	O	-11.500513	-14.456492	-24.057103
35	C	-15.364626	10.252583	-4.504901	C	-15.281903	10.456872	-4.427083	C	-8.347128	-7.658378	-15.059938
36	C	-15.327213	8.916300	-5.314841	C	-15.521019	9.073806	-5.116418	C	-9.775364	-7.710645	-15.695644
37	C	-17.195732	12.164728	-7.906325	C	-17.289891	12.338293	-7.741587	C	-9.362652	-3.656237	-13.690598
38	C	-13.862901	8.883001	-5.857352	C	-14.148721	8.799181	-5.804342	C	-9.502278	-7.395739	-17.195668
39	C	-13.391733	10.350158	-5.886446	C	-13.507798	10.180642	-6.034857	C	-8.186353	-6.601991	-17.222960
40	C	-17.158406	11.536358	-5.722726	C	-17.043857	11.878359	-5.529895	C	-8.657263	-5.799859	-13.371373
41	C	-13.338020	10.955665	-7.295661	C	-13.555760	10.659325	-7.491296	C	-8.400805	-5.095336	-17.431625

42	C	-18.341172	12.189090	-5.371668	C	-18.081807	12.706934	-5.100423	C	-8.456469	-5.660090	-11.998696
43	C	-19.066615	12.915257	-6.369212	C	-18.838844	13.443410	-6.066310	C	-8.729518	-4.396286	-11.371128
44	C	-17.568293	11.280924	-3.564574	C	-17.196497	11.841841	-3.323559	C	-7.919067	-7.699099	-12.497093
45	H	-17.412555	10.940530	-2.555754	H	-16.953376	11.559676	-2.313981	H	-7.605722	-8.728715	-12.474538
46	H	-15.156086	10.028370	-3.454286	H	-14.966522	10.280987	-3.393915	H	-7.956796	-8.680126	-15.010072
47	H	-18.821957	13.380737	-8.390562	H	-18.805782	13.718399	-8.136413	H	-9.352245	-2.478035	-11.969426
48	H	-16.989131	12.599976	-9.938995	H	-17.300682	12.586847	-9.815833	H	-10.268464	-1.836042	-14.244062
49	H	-15.675500	11.680813	-9.140000	H	-16.012220	11.569862	-9.099909	H	-9.796511	-2.993133	-15.519875
50	H	-16.083736	8.912612	-6.101889	H	-16.359817	9.123237	-5.813032	H	-10.438408	-6.988630	-15.216795
51	H	-13.803030	8.437850	-6.856091	H	-14.258668	8.254859	-6.748493	H	-10.314499	-6.814199	-17.643449
52	H	-12.412585	10.415252	-5.402344	H	-12.474569	10.152156	-5.675425	H	-7.532057	-7.018182	-17.995512
53	H	-13.269586	12.049407	-7.190219	H	-13.319091	11.734784	-7.499082	H	-7.486105	-4.579925	-17.097345
54	H	-12.436644	10.595182	-7.802974	H	-12.787044	10.127522	-8.062269	H	-8.538917	-4.902890	-18.502533
55	H	-15.305175	10.735986	-7.515674	H	-15.546071	10.707455	-7.450609	H	-9.455961	-4.885876	-15.754237
56	N	-18.363595	12.843254	-7.658406	N	-18.324470	13.181773	-7.418429	N	-9.189007	-3.422636	-12.348492
57	N	-16.582849	12.181158	-9.115301	N	-16.842513	12.183661	-9.011734	N	-9.766590	-2.689479	-14.537438
58	N	-16.596501	11.469868	-6.947435	N	-16.659453	11.645022	-6.801828	N	-9.115656	-4.862665	-14.222860
59	N	-18.585239	12.010208	-4.000429	N	-18.164360	12.665392	-3.699091	N	-7.988510	-6.874000	-11.464297
60	N	-16.661174	10.954348	-4.587753	N	-16.478039	11.321785	-4.414294	N	-8.328781	-7.094721	-13.699660
61	O	-14.371809	11.144708	-5.089771	O	-14.271991	11.160304	-5.207963	O	-7.527505	-6.794012	-15.898070
62	O	-14.479830	10.547289	-8.111798	O	-14.839355	10.368417	-8.125848	O	-9.595949	-4.617707	-16.753348
63	O	-20.116619	13.564678	-6.321288	O	-19.788004	14.226208	-5.952649	O	-8.621514	-4.053446	-10.185515
64	O	-5.514625	-3.709015	-11.086785	O	-8.901713	-1.981248	-11.743088	O	-16.590898	3.732393	-14.638097
65	O	-7.586376	-3.677917	-12.237415	O	-9.836255	-3.315246	-10.020691	O	-16.296456	1.431497	-14.160095
66	B	-6.843161	-2.979657	-11.154861	B	-9.366195	-3.382414	-11.414504	B	-16.757098	2.347557	-15.230046
67	C	-5.747000	-1.837857	-8.567919	C	-9.089752	-3.245058	-14.393253	C	-18.270119	4.021320	-17.250312
68	C	-7.162955	-1.725095	-9.221183	C	-9.666120	-4.380147	-13.483406	C	-18.153107	2.470765	-17.086141
69	C	-7.159467	0.822447	-5.426078	C	-9.458299	-5.824688	-17.820480	C	-20.485857	3.384969	-20.889719
70	C	-6.931524	-0.694017	-10.364309	C	-8.375268	-5.024021	-12.874396	C	-16.727729	2.176221	-17.648858
71	C	-5.734720	0.161009	-9.916796	C	-7.235430	-4.683142	-13.858653	C	-16.401819	3.335616	-18.608518

72	C	-6.242102	-1.117517	-6.193453	C	-9.920721	-3.920397	-16.671859	C	-20.253233	4.220152	-18.788994
73	C	-6.144770	1.536416	-9.373726	C	-6.752803	-5.872489	-14.700148	C	-16.476153	2.957653	-20.093347
74	C	-6.028901	-1.662403	-4.927874	C	-10.658248	-3.318692	-17.694238	C	-21.474794	4.895261	-18.772599
75	C	-6.414325	-0.914028	-3.764731	C	-10.848238	-4.015967	-18.928584	C	-22.330328	4.827746	-19.917759
76	C	-5.286994	-3.112550	-6.356158	C	-10.577384	-1.900900	-16.059615	C	-20.506486	5.310789	-16.881121
77	H	-4.865015	-3.976946	-6.839594	H	-10.690278	-1.043214	-15.419675	H	-20.257659	5.661016	-15.894630
78	H	-5.229831	-2.691673	-9.017030	H	-9.072374	-2.315881	-13.815647	H	-17.970705	4.494982	-16.310083
79	H	-7.245158	0.969472	-3.344184	H	-10.230521	-5.829096	-19.761456	H	-22.256267	3.977000	-21.823468
80	H	-8.129730	2.657531	-5.010780	H	-8.878800	-7.653574	-18.651094	H	-20.452461	2.494647	-22.778832
81	H	-7.698043	2.261325	-6.699028	H	-8.256782	-7.233640	-17.022232	H	-18.997922	2.326120	-21.747354
82	H	-7.907331	-1.405667	-8.490852	H	-10.268410	-5.079836	-14.068612	H	-18.951685	1.959320	-17.627027
83	H	-7.813456	-0.068452	-10.537308	H	-8.474664	-6.110662	-12.765972	H	-16.678883	1.212781	-18.167630
84	H	-5.033718	0.266357	-10.751441	H	-6.408152	-4.249065	-13.288677	H	-15.410272	3.726245	-18.360490
85	H	-5.288780	1.937145	-8.806852	H	-6.119968	-5.476106	-15.509868	H	-16.481290	3.891450	-20.677450
86	H	-6.342980	2.203529	-10.221858	H	-6.143605	-6.530675	-14.070585	H	-15.580455	2.385289	-20.357775
87	H	-7.164553	0.769950	-7.837638	H	-8.533218	-5.978594	-15.594390	H	-18.427624	2.587734	-19.933909
88	N	-6.984021	0.370410	-4.141840	N	-10.161624	-5.316414	-18.885609	N	-21.701830	4.018063	-20.971424
89	N	-7.670488	2.038858	-5.695553	N	-8.807112	-7.014446	-17.873306	N	-19.951003	2.692599	-21.925125
90	N	-6.809726	0.069513	-6.480846	N	-9.363534	-5.150142	-16.683892	N	-19.761164	3.447737	-19.779675
91	N	-5.424790	-2.926633	-5.053004	N	-11.070309	-2.040810	-17.282716	N	-21.617548	5.575891	-17.552712
92	N	-5.782334	-2.033706	-7.110811	N	-9.865016	-3.034028	-15.632322	N	-19.632294	4.472884	-17.595064
93	O	-5.050107	-0.581593	-8.819747	O	-7.756323	-3.664064	-14.813828	O	-17.412679	4.405822	-18.364563
94	O	-7.542870	-2.956562	-9.859138	O	-10.401456	-3.840379	-12.382476	O	-18.132555	2.096470	-15.703006
95	O	-6.604723	-1.527101	-11.500921	O	-8.223986	-4.355213	-11.606353	O	-15.895404	2.218246	-16.471165
96	O	-7.363239	1.474908	-8.582233	O	-7.865916	-6.670922	-15.211184	O	-17.629938	2.111612	-20.389442
97	O	-6.321089	-1.200273	-2.562955	O	-11.445403	-3.716705	-19.968865	O	-23.437955	5.321409	-20.154617
98	C	-6.055558	-2.950919	-14.040932	C	-7.754398	-2.573472	-8.900208	C	-13.956238	2.230549	-14.296386
99	C	-6.582261	-4.125891	-13.151380	C	-9.182839	-2.192751	-9.407893	C	-15.281584	2.164925	-13.465431
100	C	-5.428284	-5.756484	-17.245372	C	-8.354770	0.267030	-5.658518	C	-11.745462	2.100841	-10.584033
101	C	-5.329533	-4.485673	-12.286920	C	-8.895432	-1.169550	-10.548914	C	-15.742511	3.656065	-13.469164

102	C	-4.109100	-4.018885	-13.108019	C	-7.500345	-0.592078	-10.252627	C	-14.457964	4.489543	-13.636047
103	C	-6.363442	-3.886537	-16.357418	C	-7.884104	-1.801144	-6.494549	C	-12.290195	1.315820	-12.642778
104	C	-3.325133	-5.162254	-13.767890	C	-7.548983	0.832860	-9.686719	C	-13.962685	5.162291	-12.349388
105	C	-6.991206	-3.474433	-17.535159	C	-7.687421	-2.395461	-5.248566	C	-11.353821	0.286351	-12.631903
106	C	-6.855369	-4.265284	-18.718940	C	-7.838384	-1.604345	-4.058666	C	-10.527259	0.096300	-11.489186
107	C	-7.414937	-1.968924	-16.037670	C	-7.354976	-3.942106	-6.728981	C	-12.360947	0.200708	-14.551940
108	H	-7.766363	-1.105141	-15.500860	H	-7.149330	-4.866013	-7.241768	H	-12.686144	-0.065314	-15.541863
109	H	-6.296824	-2.005202	-13.545826	H	-7.454929	-3.506030	-9.389898	H	-14.191274	2.035282	-15.346926
110	H	-5.837638	-6.001517	-19.278732	H	-8.258215	0.397530	-3.576752	H	-10.209387	1.021906	-9.655614
111	H	-4.456451	-7.521007	-17.805167	H	-8.986253	2.224767	-5.175010	H	-11.434936	3.029194	-8.738442
112	H	-4.190608	-6.904887	-16.142465	H	-8.688795	1.803842	-6.885471	H	-12.620437	3.763827	-9.856937
113	H	-6.938820	-4.952841	-13.770025	H	-9.782318	-1.776341	-8.596814	H	-15.104097	1.761024	-12.466586
114	H	-5.268586	-5.561802	-12.085603	H	-9.643138	-0.370446	-10.575840	H	-16.267502	3.928204	-12.546468
115	H	-3.459598	-3.428856	-12.453999	H	-6.897821	-0.631163	-11.165824	H	-14.625971	5.236039	-14.418353
116	H	-2.652100	-4.720709	-14.519481	H	-6.572802	1.040260	-9.219129	H	-12.940651	5.527040	-12.537173
117	H	-2.718920	-5.662113	-13.004102	H	-7.693026	1.535834	-10.516565	H	-14.605185	6.022841	-12.131894
118	H	-4.900732	-5.635056	-14.892485	H	-8.548224	0.265433	-8.053438	H	-13.595026	3.393538	-11.503176
119	N	-6.001648	-5.433252	-18.451192	N	-8.179303	-0.230547	-4.390580	N	-10.796762	1.107270	-10.482671
120	N	-4.609119	-6.827449	-17.087881	N	-8.638954	1.563076	-5.886501	N	-11.918071	3.040544	-9.624991
121	N	-5.634826	-5.008079	-16.171182	N	-8.230560	-0.522435	-6.736902	N	-12.529623	2.194655	-11.651161
122	N	-7.657145	-2.259071	-17.308768	N	-7.353288	-3.750962	-5.419248	N	-11.412639	-0.405039	-13.845891
123	N	-6.626820	-2.943716	-15.402890	N	-7.684284	-2.776149	-7.444056	N	-12.935477	1.262963	-13.848763
124	O	-4.617794	-3.143621	-14.202664	O	-6.863248	-1.469533	-9.228987	O	-13.384567	3.555160	-14.085769
125	O	-4.208031	-6.175095	-14.345921	O	-8.663411	1.016991	-8.770136	O	-14.027095	4.274628	-11.189969
126	O	-7.298109	-4.128801	-19.865042	O	-7.715064	-1.922293	-2.867590	O	-9.654612	-0.754918	-11.247165

S2.2.2. Sγ_2-G2

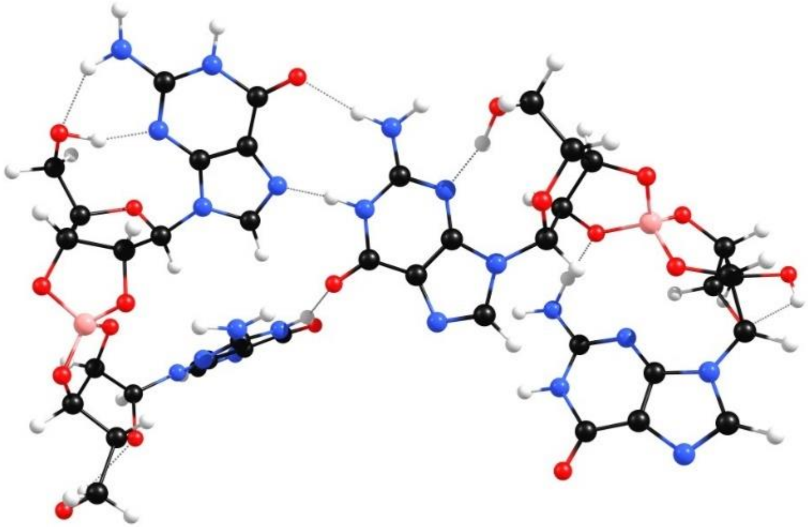

Figure S19. Optimized geometry of **Sγ_2-G2$_1$**, formed by two borate-guanosine diesters with one α-guanosine and one β-guanosine in syn conformation.

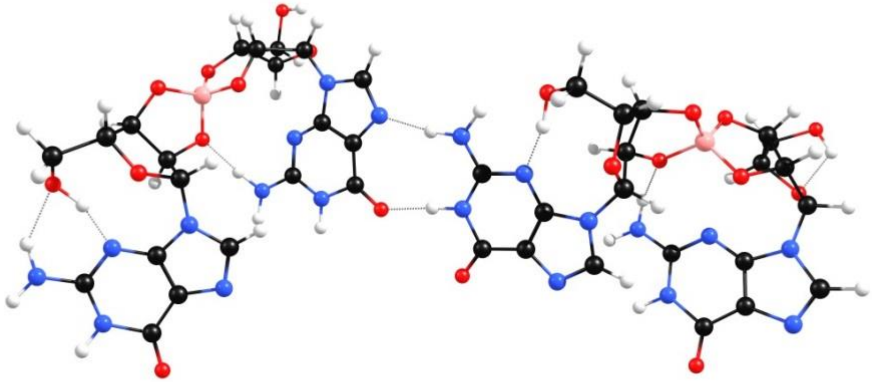

Figure S20. Optimized geometry of **Sγ_2-G2$_2$**, formed by two borate-guanosine diesters with one α-guanosine and one β-guanosine in syn conformation.

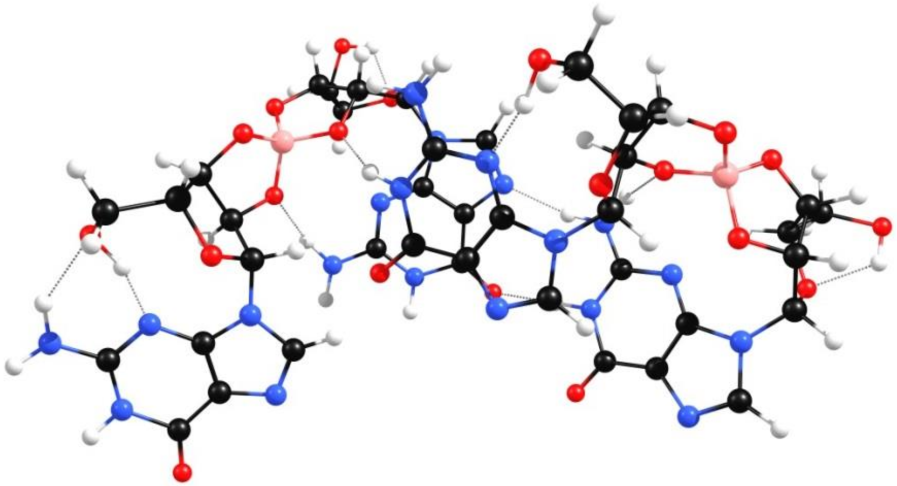

Figure S21. Optimized geometry of **Sγ$_2$-G2$_3$**, formed by two borate-guanosine diesters with one α-guanosine and one β-guanosine in syn conformation.

Table S11. Optimized xyz cartesian coordinates for **Sγ$_2$-G2** structures.

	**Sγ$_2$-G2$_1$**				**Sγ$_2$-G2$_2$**				**Sγ$_2$-G2$_3$**			
**Number**	**Atom**	**x**	**y**	**z**	**Atom**	**x**	**y**	**z**	**Atom**	**x**	**y**	**z**
1	O	-9.026962	0.028057	2.103592	O	-3.915590	0.396549	0.901439	O	-3.294857	1.021855	0.588014
2	O	-8.613357	2.140567	3.115094	O	-3.128676	1.940271	-0.723338	O	-2.907034	2.815128	-0.910543
3	B	-8.117084	1.194760	2.046979	B	-3.269792	0.470211	-0.425320	B	-2.813067	1.290470	-0.783988
4	C	-6.361015	3.588482	0.946303	C	-2.626475	-0.245840	-3.492336	C	-2.330091	0.290272	-3.945442
5	C	-6.733251	2.195601	0.352153	C	-3.187661	-1.064388	-2.289742	C	-2.730185	-0.336615	-2.578129
6	C	-3.802918	4.182378	-2.513343	C	-2.524812	-3.933060	-5.719683	C	-2.218238	-3.720400	-5.526006
7	C	-5.850024	1.217208	1.168086	C	-1.934001	-1.307710	-1.408450	C	-1.398354	-0.367645	-1.783900
8	C	-4.647713	2.048343	1.650944	C	-0.728482	-1.212061	-2.366610	C	-0.282342	-0.357714	-2.842194
9	C	-5.507073	4.755080	-1.123571	C	-3.202754	-1.787967	-5.398035	C	-2.973596	-1.577304	-5.508563
10	C	-3.358570	1.783478	0.863658	C	-0.102022	-2.567637	-2.721545	C	0.410299	-1.717709	-3.009840

11	C	-5.800274	5.953855	-1.788623	C	-3.908906	-1.685678	-6.596607	C	-3.768566	-1.697974	-6.648860
12	C	-5.027667	6.300282	-2.934725	C	-3.961042	-2.810275	-7.481961	C	-3.842970	-2.958526	-7.321502
13	C	-7.149270	5.840444	-0.096928	C	-4.045931	0.248046	-5.628621	C	-3.899717	0.378769	-6.018178
14	H	-7.906950	6.029580	0.643295	H	-4.251018	1.275746	-5.384346	H	-4.066587	1.441942	-5.926296
15	H	-7.090668	3.839441	1.723396	H	-2.776984	0.819846	-3.293678	H	-2.548048	1.358783	-3.948341
16	H	-3.459338	5.536497	-4.093696	H	-3.148242	-4.733537	-7.543631	H	-2.956298	-4.841818	-7.123089
17	H	-2.087793	3.539435	-3.546192	H	-1.752047	-5.871903	-5.784363	H	-1.340445	-5.608797	-5.350207
18	H	-2.690612	2.553068	-2.184157	H	-1.305429	-4.838533	-4.391951	H	-0.821727	-4.337034	-4.203970
19	H	-6.540737	2.161944	-0.721760	H	-3.660218	-1.987353	-2.633028	H	-3.166314	-1.329193	-2.712992
20	H	-5.520088	0.364570	0.563846	H	-1.963864	-2.287653	-0.918416	H	-1.325754	-1.252737	-1.142629
21	H	-4.503651	1.863447	2.719486	H	0.011863	-0.540006	-1.923666	H	0.442942	0.420435	-2.583988
22	H	-2.662339	2.610867	1.075169	H	0.512378	-2.426474	-3.623071	H	0.986122	-1.683949	-3.947745
23	H	-2.909940	0.850861	1.226305	H	0.546134	-2.886918	-1.898531	H	1.105735	-1.867242	-2.176208
24	H	-4.142157	2.455852	-0.842352	H	-1.795178	-3.188871	-3.557167	H	-1.294492	-2.553073	-3.644355
25	N	-4.037453	5.323879	-3.231118	N	-3.180936	-3.926804	-6.924171	N	-2.974741	-3.942512	-6.647866
26	N	-2.792013	3.349416	-2.825699	N	-1.799709	-4.993681	-5.288922	N	-1.397392	-4.664665	-4.998249
27	N	-4.567212	3.855337	-1.458555	N	-2.560775	-2.874161	-4.918016	N	-2.241462	-2.544735	-4.911970
28	N	-6.845612	6.618296	-1.122709	N	-4.440700	-0.392839	-6.718913	N	-4.349122	-0.453716	-6.949066
29	N	-6.366229	4.668423	-0.055564	N	-3.289706	-0.572857	-4.769874	N	-3.062859	-0.280295	-5.088152
30	O	-5.005348	3.485468	1.462434	O	-1.227090	-0.604304	-3.636946	O	-0.916284	-0.021054	-4.145498
31	O	-8.096142	1.842388	0.660111	O	-4.085868	-0.274578	-1.491616	O	-3.615743	0.528108	-1.830766
32	O	-6.689608	0.838633	2.273752	O	-1.944643	-0.207405	-0.486863	O	-1.418993	0.871083	-1.039426
33	O	-3.608812	1.611511	-0.560081	O	-1.115209	-3.607124	-2.897987	O	-0.543178	-2.822698	-2.979462
34	O	-5.099567	7.319201	-3.675629	O	-4.509227	-2.979305	-8.573618	O	-4.463254	-3.335956	-8.319969
35	C	-10.874658	2.188453	4.336416	C	-4.284004	3.948003	0.381493	C	-4.523407	4.432557	0.371086
36	C	-9.529345	1.454398	3.969396	C	-3.344103	2.688581	0.476051	C	-3.317606	3.413059	0.342437
37	C	-10.273605	4.295434	0.561716	C	-6.042997	1.830039	-3.028222	C	-6.014646	2.393180	-3.187079
38	C	-9.994078	0.209522	3.142371	C	-4.120357	1.714309	1.422887	C	-3.824473	2.203979	1.186813
39	C	-11.369522	0.618406	2.594121	C	-5.585100	2.162094	1.298704	C	-5.353983	2.298984	1.073323
40	C	-10.824103	4.168792	2.737696	C	-5.254007	3.645331	-1.960398	C	-5.569753	4.201337	-1.931601

41	C	-12.334266	-0.552793	2.439133	C	-6.389639	1.962769	2.576631	C	-6.077155	1.754823	2.298269
42	C	-11.068290	5.537408	2.900928	C	-5.439668	4.514041	-3.038307	C	-6.029029	5.121071	-2.871585
43	C	-10.974960	6.410782	1.769613	C	-6.040135	4.033642	-4.235474	C	-6.603305	4.655041	-4.090763
44	C	-11.302564	4.655157	4.857781	C	-4.489116	5.681128	-1.483975	C	-5.190076	6.295398	-1.256786
45	H	-11.471040	4.474926	5.906632	H	-4.039618	6.470044	-0.906293	H	-4.846589	7.094673	-0.623019
46	H	-11.040945	2.140200	5.416764	H	-3.802399	4.810287	0.851556	H	-4.223605	5.312076	0.947440
47	H	-10.331178	6.247204	-0.207555	H	-6.660483	2.213129	-5.006863	H	-6.795653	2.848060	-5.071469
48	H	-9.192130	2.839906	-0.268177	H	-5.551117	0.018576	-2.363146	H	-5.150410	0.669933	-2.739970
49	H	-9.050271	1.152117	4.913807	H	-2.393216	3.016375	0.921595	H	-2.460987	3.912752	0.810986
50	H	-10.121257	-0.684598	3.763471	H	-3.815506	1.806384	2.470500	H	-3.559791	2.293455	2.245661
51	H	-11.248909	1.235715	1.704905	H	-6.031810	1.727662	0.405128	H	-5.691912	1.893582	0.120200
52	H	-13.271382	-0.209145	1.974624	H	-7.436845	2.258383	2.412797	H	-7.165350	1.792361	2.139756
53	H	-11.875805	-1.316324	1.804762	H	-6.352991	0.908958	2.865139	H	-5.772107	0.718072	2.464281
54	H	-12.616729	-0.308295	4.325733	H	-5.563470	3.602089	3.175381	H	-5.676112	3.467384	3.094565
55	N	-10.505639	5.664728	0.610011	N	-6.279782	2.619558	-4.153318	N	-6.558408	3.219030	-4.153428
56	N	-9.710751	3.716716	-0.524306	N	-6.165225	0.486616	-3.079198	N	-5.869922	1.066631	-3.385665
57	N	-10.531679	3.513917	1.602515	N	-5.619330	2.358980	-1.887284	N	-5.610199	2.867522	-2.008071
58	N	-11.371269	5.819992	4.243167	N	-4.957776	5.787978	-2.716354	N	-5.783829	6.425895	-2.428630
59	N	-10.957689	3.600239	3.987546	N	-4.632177	4.383250	-0.973470	N	-5.016532	4.940882	-0.901454
60	O	-11.961035	1.426677	3.710058	O	-5.485483	3.651971	1.156825	O	-5.597503	3.775217	1.123945
61	O	-12.570545	-1.136547	3.759975	O	-5.770240	2.742669	3.647680	O	-5.675437	2.536782	3.468197
62	O	-11.195430	7.623429	1.654752	O	-6.341525	4.635132	-5.285169	O	-7.092575	5.290996	-5.041119
63	H	-9.421102	4.271990	-1.321142	H	-6.429934	0.011160	-3.933833	H	-5.989615	0.661244	-4.307699
64	O	-2.210370	13.676273	-6.613979	O	-8.092186	16.049741	-5.743195	O	-3.968070	12.942690	-4.387327
65	O	-2.850118	11.973011	-8.157010	O	-7.306157	15.592701	-7.939650	O	-3.357404	11.525842	-6.198707
66	B	-1.856218	12.328412	-7.061458	B	-7.305622	15.062980	-6.526433	B	-3.259570	11.684723	-4.687829
67	C	-1.751606	9.153963	-7.292604	C	-6.135616	12.256348	-7.450270	C	-2.245154	8.917626	-5.124393
68	C	-1.002276	10.218235	-6.428031	C	-6.828478	12.733596	-6.136206	C	-2.730660	9.577052	-3.787475
69	C	1.358839	6.167285	-6.651387	C	-5.846612	8.349668	-5.540714	C	-1.868429	5.380100	-2.667610
70	C	0.007416	10.902719	-7.428760	C	-5.721060	13.568506	-5.461953	C	-1.537796	10.500831	-3.455855

71	C	-0.112642	10.107009	-8.758338	C	-4.397337	12.951758	-5.942336	C	-0.299513	9.773580	-3.990537
72	C	-0.754501	6.982272	-6.451329	C	-6.485125	9.760630	-7.212330	C	-2.537709	6.496559	-4.513183
73	C	1.219520	9.538692	-9.263967	C	-3.762137	11.998079	-4.923144	C	0.448677	8.975655	-2.911209
74	C	-1.209004	5.906917	-5.691645	C	-7.124182	8.778438	-7.965679	C	-3.152372	5.385093	-5.095771
75	C	-0.327004	4.850093	-5.344531	C	-7.148129	7.424255	-7.481153	C	-3.160143	4.150813	-4.383082
76	C	-2.895200	7.257683	-5.941779	C	-7.307232	10.627796	-9.084620	C	-3.479476	7.046932	-6.443833
77	H	-3.864838	7.712356	-5.880135	H	-7.531125	11.381200	-9.819155	H	-3.800727	7.685169	-7.248682
78	H	-2.764181	9.511047	-7.491186	H	-6.351877	12.984737	-8.238608	H	-2.560310	9.544067	-5.963835
79	H	1.643248	4.309292	-5.724102	H	-6.383720	6.349749	-5.841247	H	-2.692278	3.520889	-2.392574
80	H	3.360801	5.664127	-6.964521	H	-5.132136	7.280770	-3.854881	H	-1.113796	4.445866	-1.000155
81	H	2.746834	7.160555	-7.727254	H	-4.795273	9.032171	-3.990114	H	-0.676659	6.171556	-1.235142
82	H	-0.501514	9.732817	-5.584087	H	-7.160100	11.887935	-5.532825	H	-2.950176	8.830328	-3.026239
83	H	1.037661	10.851494	-7.064596	H	-5.794738	13.548824	-4.368347	H	-1.452554	10.702515	-2.381167
84	H	-0.589500	10.757525	-9.495906	H	-3.707511	13.753967	-6.222261	H	0.367128	10.493163	-4.477313
85	H	1.008294	8.841953	-10.089260	H	-2.986622	11.418460	-5.450524	H	1.149212	8.296811	-3.426770
86	H	1.834766	10.361310	-9.643322	H	-3.274089	12.594189	-4.141300	H	1.034024	9.675651	-2.301375
87	H	1.246005	8.374761	-7.650432	H	-5.254872	10.659220	-5.036291	H	-1.058381	7.695186	-2.617034
88	N	0.984244	5.060849	-5.914649	N	-6.435044	7.313391	-6.218164	N	-2.516241	4.263109	-3.116353
89	N	2.603057	6.299677	-7.169048	N	-5.201588	8.171815	-4.374038	N	-1.197598	5.329244	-1.489704
90	N	0.505065	7.151727	-6.897617	N	-5.881345	9.606317	-6.019214	N	-1.865046	6.526511	-3.341933
91	N	-2.551942	6.104114	-5.379064	N	-7.645160	9.348122	-9.142690	N	-3.732915	5.754595	-6.318640
92	N	-1.818669	7.836050	-6.610922	N	-6.608009	10.943927	-7.906438	N	-2.752730	7.559058	-5.347642
93	O	-0.983039	8.928432	-8.498645	O	-4.714654	12.150228	-7.162465	O	-0.787744	8.807584	-5.014551
94	O	-1.884333	11.256825	-5.980830	O	-7.902176	13.655054	-6.416286	O	-3.838777	10.473858	-3.984411
95	O	-0.447588	12.253240	-7.560075	O	-5.914772	14.880680	-6.030028	O	-1.831081	11.684562	-4.235893
96	O	1.959916	8.894937	-8.178932	O	-4.750413	11.158766	-4.270650	O	-0.452640	8.275623	-2.010823
97	O	-0.523278	3.827823	-4.655964	O	-7.662249	6.414579	-7.977134	O	-3.657162	3.041629	-4.702348
98	C	-5.097844	13.098483	-8.711445	C	-8.895223	17.284349	-9.046500	C	-5.112432	12.543099	-7.795819
99	C	-3.529401	13.161237	-8.565637	C	-7.813582	16.927978	-7.957143	C	-3.908630	12.701633	-6.784028
100	C	-5.039039	10.025485	-5.579159	C	-9.804296	13.026165	-8.987816	C	-6.231819	8.939047	-5.523133

101	C	-3.300893	14.181016	-7.397985	C	-8.583150	17.078799	-6.604562	C	-4.523448	13.499879	-5.582863
102	C	-4.625483	14.170488	-6.625086	C	-10.053159	16.860442	-6.992702	C	-6.034928	13.254924	-5.706082
103	C	-5.699670	10.914759	-7.535209	C	-9.429089	14.991931	-10.008976	C	-5.894040	10.139466	-7.401596
104	C	-4.962054	15.509481	-5.979997	C	-11.030723	17.639816	-6.121234	C	-6.880693	14.426321	-5.218447
105	C	-6.600972	9.889915	-7.840084	C	-9.510554	14.491393	-11.313924	C	-6.275943	9.114102	-8.274965
106	C	-6.813060	8.823798	-6.897858	C	-9.827598	13.109175	-11.519286	C	-6.772488	7.876225	-7.734071
107	C	-6.656046	11.248457	-9.519420	C	-8.970431	16.574335	-11.506709	C	-5.652891	10.759365	-9.533527
108	H	-6.870104	11.746531	-10.450663	H	-8.703661	17.552482	-11.871733	H	-5.403390	11.403616	-10.361326
109	H	-5.396021	13.491092	-9.687927	H	-8.629548	18.227685	-9.533783	H	-4.883804	13.078229	-8.722420
110	H	-5.967550	8.247889	-5.039124	H	-10.021859	11.417738	-10.313227	H	-6.845760	6.962351	-5.843803
111	H	-3.334062	10.561583	-4.673194	H	-9.236822	12.776703	-7.099998	H	-5.399792	9.558493	-3.854569
112	H	-3.129142	13.551311	-9.515026	H	-7.000295	17.665483	-8.043418	H	-3.134540	13.293915	-7.298365
113	H	-3.118499	15.199524	-7.757910	H	-8.483836	18.079253	-6.167948	H	-4.343686	14.578267	-5.663015
114	H	-4.656992	13.313828	-5.952420	H	-10.262622	15.795102	-7.079854	H	-6.292460	12.283015	-5.284827
115	H	-5.886653	15.419427	-5.389898	H	-12.066481	17.382244	-6.390835	H	-7.949112	14.164330	-5.264922
116	H	-4.142439	15.809525	-5.321202	H	-10.857842	17.385220	-5.071778	H	-6.611654	14.661902	-4.184788
117	H	-5.570490	15.987762	-7.742400	H	-10.576741	19.106500	-7.279447	H	-6.475467	15.155228	-6.949473
118	N	-5.929890	8.979499	-5.764068	N	-9.913191	12.428180	-10.237213	N	-6.657877	7.873471	-6.292062
119	N	-4.161996	9.964080	-4.550663	N	-9.778757	12.283750	-7.855616	N	-6.053585	8.825981	-4.193833
120	N	-4.960117	11.051955	-6.418842	N	-9.661741	14.339399	-8.860057	N	-5.938701	10.132765	-6.066412
121	N	-7.188129	10.119244	-9.095662	N	-9.222989	15.507545	-12.241002	N	-6.117296	9.529098	-9.610925
122	N	-5.718290	11.784645	-8.610096	N	-9.069033	16.318450	-10.123605	N	-5.476697	11.195885	-8.199957
123	O	-5.649335	14.019175	-7.708548	O	-10.159382	17.515418	-8.337812	O	-6.253090	13.248683	-7.189109
124	O	-5.092145	16.515808	-7.035460	O	-10.771642	19.070405	-6.294963	O	-6.578036	15.593174	-6.050724
125	O	-7.585144	7.856497	-6.947242	O	-9.997566	12.457841	-12.559545	O	-7.235717	6.888017	-8.322051
126	H	-4.218477	9.190751	-3.890740	H	-9.744036	11.270967	-7.913677	H	-6.116180	7.940329	-3.678505

S2.2.3. Sα1-G2

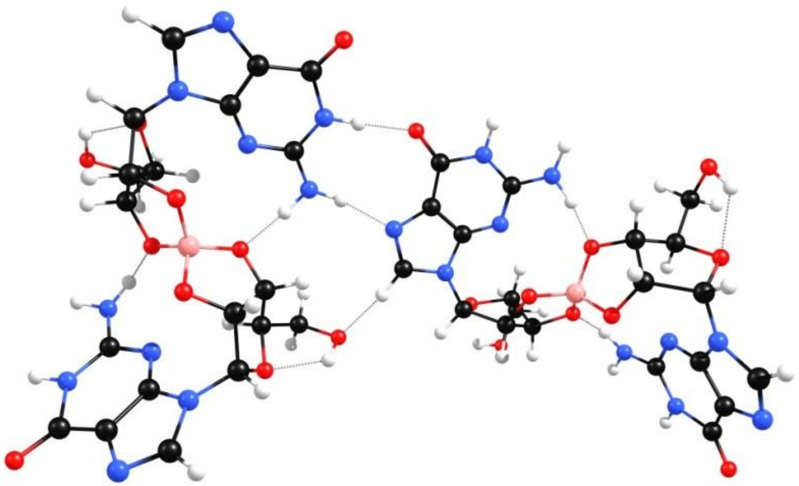

Figure S22. Optimized geometry of **Sα1-G2$_1$**, formed by two borate-guanosine diesters with two α-guanosines in syn conformation.

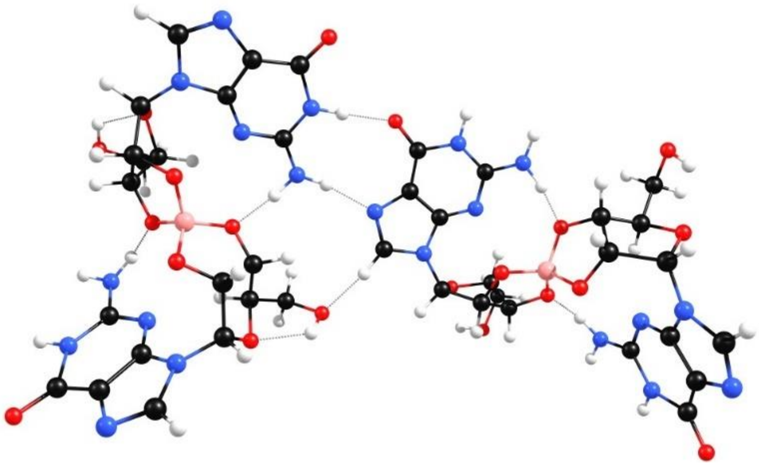

Figure S23. Optimized geometry of **Sα1-G2$_2$**, formed by two borate-guanosine diesters with two α-guanosines in syn conformation.

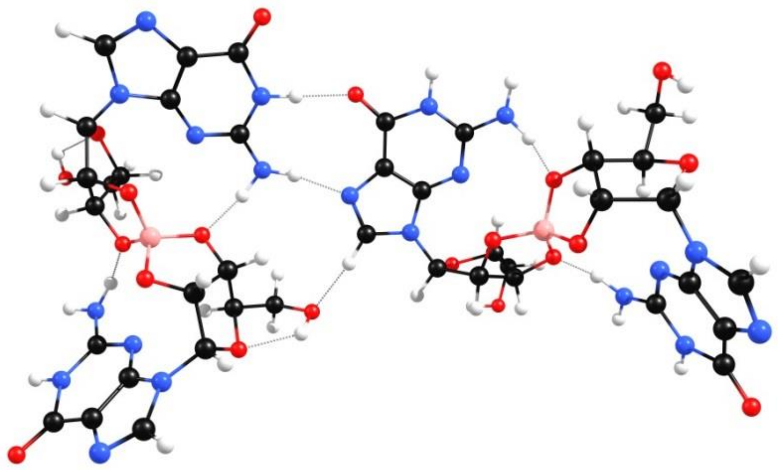

Figure S24. Optimized geometry of **Sα1-G23**, formed by two borate-guanosine diesters with two α-guanosines in syn conformation.

Table S12. Optimized xyz cartesian coordinates for **Sα1-G2** structures.

	**Sα1-G21**				**Sα1-G22**				**Sα1-G23**			
**Number**	**Atom**	**x**	**y**	**z**	**Atom**	**x**	**y**	**z**	**Atom**	**x**	**y**	**z**
1	C	5.283687	-0.413032	-2.734188	C	4.193375	-5.272536	2.268170	C	3.539338	-5.132171	1.842851
2	C	4.463004	-1.416752	-1.837199	C	4.874656	-4.018510	1.602426	C	4.292022	-3.903447	1.206725
3	C	8.860616	-2.439840	-1.253093	C	1.559580	-5.143744	-1.200142	C	0.931927	-4.775726	-1.631421
4	C	4.155569	-2.596981	-2.796220	C	5.986582	-4.631022	0.698135	C	5.375661	-4.556909	0.296303
5	C	5.328320	-2.563901	-3.786456	C	5.465540	-6.045019	0.391899	C	4.773839	-5.930270	-0.044855
6	C	7.635080	-0.634409	-1.793510	C	1.992581	-5.354023	0.994516	C	1.342445	-5.065637	0.558807
7	C	4.967980	-3.119814	-5.159785	C	6.553099	-7.089455	0.160771	C	5.795217	-7.032948	-0.306669
8	C	8.575638	0.298335	-1.345730	C	0.652298	-5.421230	1.395264	C	-0.000955	-5.064218	0.954106
9	C	9.841877	-0.166169	-0.847490	C	-0.380234	-5.400614	0.404032	C	-1.026257	-4.959266	-0.039343
10	C	6.852260	1.451851	-1.966517	C	1.818025	-5.422923	3.214094	C	1.155262	-5.173377	2.775751
11	H	6.148403	2.235859	-2.195061	H	2.150747	-5.433938	4.238736	H	1.482620	-5.226582	3.800892

12	H	4.663634	0.462760	-2.951341	H	4.412938	-5.292107	3.339279	H	3.751432	-5.184451	2.914408
13	H	10.742192	-2.018916	-0.432820	H	-0.486575	-5.107149	-1.648629	H	-1.106863	-4.606313	-2.083772
14	H	9.591239	-4.210708	-0.410536	H	1.338237	-4.581166	-3.170008	H	0.753421	-4.156077	-3.588915
15	H	7.967216	-4.178735	-1.116171	H	2.917707	-4.299531	-2.375607	H	2.343397	-3.983215	-2.782590
16	H	3.546493	-0.904376	-1.506079	H	5.299422	-3.395676	2.402787	H	4.742240	-3.318479	2.021387
17	H	3.220642	-2.486335	-3.354859	H	6.963648	-4.687805	1.188418	H	6.344711	-4.680614	0.790872
18	H	6.230831	-2.975770	-3.333883	H	4.709724	-5.995807	-0.391640	H	4.019881	-5.816582	-0.823446
19	H	5.853677	-3.114032	-5.812154	H	6.106266	-8.039166	-0.162679	H	5.290348	-7.941415	-0.661563
20	H	4.606560	-4.147440	-5.056775	H	7.252558	-6.736166	-0.600426	H	6.519211	-6.701027	-1.054574
21	H	4.175307	-1.389118	-5.421786	H	6.570137	-7.131374	2.099921	H	5.802904	-7.134573	1.630993
22	N	9.877253	-1.608738	-0.823071	N	0.205409	-5.229544	-0.912980	N	-0.426189	-4.788917	-1.350074
23	N	8.915949	-3.771244	-1.056884	N	2.000641	-4.823525	-2.441529	N	1.396710	-4.449512	-2.862341
24	N	7.760286	-1.962829	-1.855179	N	2.475555	-5.319369	-0.257544	N	1.831354	-5.030164	-0.690946
25	N	8.062077	1.603080	-1.467834	N	0.567531	-5.468263	2.796905	N	-0.093989	-5.137800	2.354139
26	N	6.519332	0.093234	-2.178421	N	2.742956	-5.341141	2.151860	N	2.088015	-5.120552	1.718366
27	O	5.502425	-1.097174	-4.024083	O	4.831617	-6.461506	1.685445	O	4.117975	-6.341064	1.239429
28	O	5.145708	-2.020321	-0.739018	O	4.064115	-3.249496	0.713268	O	3.529128	-3.078221	0.325836
29	O	4.152902	-3.780351	-1.971648	O	6.031281	-3.809410	-0.480249	O	5.476156	-3.713177	-0.862433
30	O	3.882770	-2.305752	-5.712430	O	7.292871	-7.272855	1.416479	O	6.516558	-7.303963	0.944087
31	O	10.822660	0.483925	-0.450388	O	-1.614866	-5.474117	0.502124	O	-2.263467	-4.963182	0.053550
32	B	4.914742	-3.494408	-0.681057	B	4.815507	-2.913172	-0.539411	B	4.309780	-2.752401	-0.910927
33	O	6.185067	-4.309766	-0.606466	O	4.006981	-3.169793	-1.807108	O	3.504177	-2.937907	-2.193034
34	O	4.157901	-3.897033	0.548490	O	5.140229	-1.458353	-0.627078	O	4.709078	-1.314428	-0.960545
35	C	4.385282	-5.931814	2.077313	C	5.326358	0.105021	-2.657461	C	4.994711	0.287352	-2.946459
36	C	4.996543	-4.623711	1.446173	C	4.468742	-0.827368	-1.716337	C	4.080319	-0.624725	-2.039731
37	C	1.837147	-5.936020	-1.457281	C	8.802237	-2.185611	-1.304013	C	8.332802	-2.207515	-1.610469
38	C	6.170469	-5.146257	0.562708	C	4.049360	-1.998099	-2.646301	C	3.616650	-1.750388	-3.003058
39	C	5.758327	-6.589283	0.227605	C	5.179620	-2.051273	-3.683081	C	4.758346	-1.834035	-4.026322
40	C	2.228953	-6.149311	0.744886	C	7.697624	-0.296710	-1.823594	C	7.329861	-0.252705	-2.091015
41	C	6.919652	-7.555970	0.016669	C	4.737540	-2.604382	-5.032155	C	4.308281	-2.332988	-5.393729

42	C	0.888111	-6.326598	1.108575	C	8.733685	0.564154	-1.448579	C	8.400277	0.545105	-1.675003
43	C	-0.117362	-6.368913	0.090486	C	9.984072	0.007687	-1.008488	C	9.614371	-0.084340	-1.231428
44	C	2.004213	-6.272012	2.957636	C	7.078871	1.843296	-2.004885	C	6.819480	1.919544	-2.219386
45	H	2.310879	-6.276992	3.990463	H	6.429758	2.677903	-2.215342	H	6.215512	2.790831	-2.414956
46	H	4.579088	-5.952273	3.153495	H	4.770823	1.027142	-2.853699	H	4.487201	1.239726	-3.128270
47	H	-0.193719	-6.049481	-1.959774	H	10.752812	-1.902815	-0.593235	H	10.281124	-2.041481	-0.858264
48	H	3.156717	-4.973452	-2.585568	H	7.776251	-3.837931	-1.071452	H	7.225289	-3.814318	-1.442170
49	H	5.356878	-3.987482	2.267317	H	3.599550	-0.247347	-1.369479	H	3.236609	-0.011311	-1.687609
50	H	7.137132	-5.139362	1.076457	H	3.101062	-1.834076	-3.168085	H	2.684456	-1.528033	-3.532489
51	H	5.025154	-6.583692	-0.578629	H	6.074575	-2.504911	-3.255114	H	5.624312	-2.339792	-3.596780
52	H	6.546290	-8.529659	-0.328116	H	5.597244	-2.651513	-5.716987	H	5.174477	-2.410535	-6.067499
53	H	7.613698	-7.148755	-0.722442	H	4.328335	-3.610847	-4.902586	H	3.844833	-3.318999	-5.292593
54	H	6.887506	-7.620664	1.954650	H	4.024540	-0.837997	-5.291334	H	3.693037	-0.526246	-5.617724
55	N	0.486639	-6.130712	-1.207775	N	9.904230	-1.431932	-0.949594	N	9.463914	-1.519352	-1.216151
56	N	2.283628	-5.563137	-2.681984	N	8.758777	-3.512139	-1.075916	N	8.222064	-3.536617	-1.424137
57	N	2.739432	-6.055518	-0.493103	N	7.713108	-1.631936	-1.860122	N	7.281148	-1.585465	-2.166147
58	N	0.771885	-6.405332	2.506628	N	8.322179	1.904024	-1.574498	N	8.056865	1.906526	-1.767909
59	N	2.946612	-6.100113	1.921943	N	6.624737	0.512944	-2.166082	N	6.304078	0.618320	-2.427043
60	O	5.118143	-7.065137	1.496791	O	5.428767	-0.599618	-3.951101	O	5.080874	-0.390535	-4.255793
61	O	7.636431	-7.703771	1.290054	O	3.675413	-1.742081	-5.557003	O	3.301938	-1.403454	-5.913612
62	O	-1.344531	-6.540141	0.154373	O	11.034798	0.583344	-0.681887	O	10.686023	0.429667	-0.871512
63	H	1.623599	-5.362817	-3.425217	H	9.429338	-3.990349	-0.452678	H	8.857719	-4.065499	-0.805374
64	C	11.601964	-8.180914	2.716865	C	11.273203	-7.986263	2.765401	C	14.581177	-6.446018	8.099611
65	C	12.139480	-8.168229	4.199861	C	11.838586	-7.938777	4.237176	C	13.698133	-6.212362	6.813576
66	C	14.466599	-4.912604	2.120610	C	14.336782	-4.966471	1.939431	C	14.104358	-10.678130	7.175191
67	C	13.337966	-9.161095	4.167604	C	12.952052	-9.024366	4.235886	C	14.742682	-6.106101	5.666609
68	C	13.782834	-9.170366	2.699322	C	13.385737	-9.109484	2.767740	C	15.947731	-6.900105	6.187885
69	C	12.486170	-5.946794	1.867307	C	12.278896	-5.860785	1.789540	C	14.152214	-8.910612	8.561156
70	C	14.365615	-10.506886	2.254892	C	13.891167	-10.489367	2.367618	C	17.284979	-6.391960	5.663376
71	C	11.899388	-4.908149	1.143213	C	11.745013	-4.812633	1.039097	C	13.770703	-9.620809	9.705742

72	C	12.659888	-3.751485	0.821821	C	12.577774	-3.730153	0.645138	C	13.574367	-11.036577	9.622628
73	C	10.336551	-6.390272	1.427322	C	10.090149	-6.164560	1.433758	C	13.880704	-7.547413	10.301948
74	H	9.393302	-6.929765	1.428649	H	9.111284	-6.633121	1.485791	H	13.857087	-6.615547	10.842166
75	H	10.601246	-8.616862	2.674696	H	10.248201	-8.364212	2.761648	H	14.525348	-5.567666	8.748922
76	H	14.527929	-2.989512	1.274751	H	14.510019	-3.089927	1.009871	H	13.522353	-12.459491	8.111045
77	H	16.202767	-3.987642	2.749625	H	16.159032	-4.154082	2.479532	H	13.757138	-12.091844	5.713866
78	H	15.670927	-5.475481	3.591059	H	15.542311	-5.565571	3.394239	H	13.884190	-10.375941	5.228672
79	H	11.331437	-8.530047	4.851731	H	11.019376	-8.191634	4.924734	H	13.142298	-5.273234	6.949639
80	H	13.063935	-10.179731	4.459071	H	12.603305	-10.011070	4.556634	H	15.058620	-5.079853	5.455035
81	H	14.403276	-8.299357	2.487551	H	14.052395	-8.283374	2.520474	H	15.777805	-7.969300	6.063856
82	H	14.669668	-10.450323	1.199353	H	14.204959	-10.482397	1.313458	H	18.101335	-7.020458	6.049996
83	H	15.239949	-10.748964	2.866378	H	14.745854	-10.762963	2.993389	H	17.292136	-6.433447	4.570233
84	H	12.529134	-11.087792	2.150755	H	12.023349	-10.966760	2.269983	H	17.103538	-5.021057	7.006687
85	N	13.975538	-3.836091	1.393020	N	13.901959	-3.887953	1.180515	N	13.749236	-11.477339	8.249863
86	N	15.667247	-4.848190	2.746445	N	15.557506	-4.968311	2.528142	N	14.095221	-11.155511	5.905497
87	N	13.762171	-6.026195	2.276736	N	13.558162	-6.016817	2.163659	N	14.419773	-9.400718	7.339980
88	N	10.560807	-5.210788	0.869650	N	10.379746	-5.028153	0.819032	N	13.610428	-8.742080	10.790052
89	N	11.491903	-6.883369	2.065059	N	11.227828	-6.713743	2.057733	N	14.210809	-7.582585	8.930369
90	O	12.485264	-9.082303	1.962444	O	12.089897	-8.968961	2.036685	O	15.973070	-6.548152	7.643569
91	O	12.688772	-6.941791	4.685089	O	12.503569	-6.738815	4.640470	O	12.835455	-7.279560	6.424626
92	O	14.339386	-8.600042	5.041099	O	14.000627	-8.516878	5.086268	O	14.133166	-6.732990	4.520274
93	O	13.360525	-11.545683	2.475998	O	12.826397	-11.463890	2.608889	O	17.441957	-4.994019	6.062775
94	O	12.349611	-2.728644	0.176971	O	12.323506	-2.714420	-0.034616	O	13.272605	-11.875586	10.483975
95	B	13.975377	-7.172506	5.407190	B	13.780325	-7.036998	5.353891	B	12.918349	-7.540435	4.952689
96	O	15.069613	-6.193500	5.014228	O	14.947879	-6.192380	4.862955	O	13.074117	-9.016844	4.630754
97	O	13.844651	-6.970169	6.885248	O	13.716196	-6.719807	6.814921	O	11.671809	-7.153450	4.228227
98	C	15.426914	-5.921640	8.603690	C	15.460457	-5.746053	8.418303	C	10.414222	-8.269108	2.292858
99	C	14.533060	-5.796232	7.310819	C	14.542760	-5.604461	7.144291	C	10.954715	-8.304050	3.774080
100	C	15.625256	-10.148079	7.542781	C	15.142018	-10.028355	7.682737	C	13.652399	-5.389666	1.626584
101	C	15.561879	-5.503456	6.179954	C	15.559210	-5.503126	5.971763	C	12.013358	-9.443565	3.751131

102	C	16.875376	-6.098862	6.706540	C	16.805183	-6.227872	6.497849	C	12.463299	-9.501449	2.286521
103	C	15.392800	-8.439214	8.982913	C	15.144542	-8.203766	8.992899	C	11.553753	-6.167966	1.412151
104	C	18.115912	-5.363837	6.213352	C	18.109470	-5.691625	5.923107	C	12.906304	-10.890432	1.844844
105	C	15.133587	-9.236278	10.104179	C	14.817250	-8.879798	10.174171	C	11.089618	-5.065254	0.694003
106	C	15.166212	-10.661925	9.977040	C	14.678913	-10.304876	10.154053	C	11.984135	-4.014331	0.353237
107	C	14.918586	-7.191265	10.766311	C	14.854641	-6.780173	10.681274	C	9.357741	-6.337953	1.012826
108	H	14.750578	-6.292539	11.336349	H	14.804601	-5.828064	11.182805	H	8.353798	-6.753227	1.036156
109	H	15.221096	-5.086935	9.279789	H	15.381980	-4.844067	9.032234	H	9.370007	-8.588656	2.259279
110	H	15.336821	-12.027313	8.420858	H	14.655555	-11.791232	8.703655	H	13.941908	-3.493113	0.769490
111	H	15.355262	-9.825588	5.604140	H	14.871331	-9.815403	5.730533	H	14.802893	-6.094105	3.079799
112	H	13.840042	-4.955253	7.458516	H	13.951219	-4.683737	7.252581	H	10.112458	-8.539945	4.439336
113	H	15.710521	-4.435286	5.992878	H	15.831239	-4.474556	5.715322	H	11.612419	-10.421231	4.035597
114	H	16.888008	-7.179263	6.563813	H	16.675943	-7.307271	6.423374	H	13.174393	-8.701906	2.078799
115	H	19.020089	-5.849103	6.609984	H	18.958800	-6.257715	6.333826	H	13.236672	-10.861819	0.796052
116	H	18.149444	-5.386280	5.120156	H	18.104564	-5.796504	4.834363	H	13.736556	-11.228871	2.471813
117	H	17.669020	-4.066481	7.566984	H	17.890174	-4.251641	7.188556	H	11.018688	-11.268705	1.705321
118	N	15.404608	-11.026537	8.591379	N	14.843681	-10.795779	8.796998	N	13.288790	-4.262432	0.901443
119	N	15.690515	-10.580596	6.259148	N	15.126330	-10.557509	6.434015	N	14.862256	-5.474063	2.232783
120	N	15.732457	-8.841998	7.748111	N	15.406615	-8.733238	7.787453	N	12.817065	-6.404777	1.800173
121	N	14.842078	-8.428347	11.215919	N	14.644374	-7.963079	11.224605	N	9.718906	-5.197133	0.446262
122	N	15.243082	-7.130576	9.393884	N	15.156098	-6.860262	9.304872	N	10.453461	-6.972043	1.631215
123	O	16.820942	-5.771452	8.166692	O	16.845554	-5.809060	7.935376	O	11.187535	-9.271487	1.544138
124	O	18.025377	-3.965601	6.634622	O	18.207635	-4.266242	6.237381	O	11.790703	-11.817869	2.035942
125	O	15.006203	-11.564469	10.811760	O	14.431078	-11.118495	11.055954	O	11.795139	-2.961919	-0.291202
126	H	15.514048	-11.553672	6.035941	H	14.825713	-11.514404	6.286711	H	15.501207	-4.687574	2.220781

S3. G3

S3.1. Anti Conformation

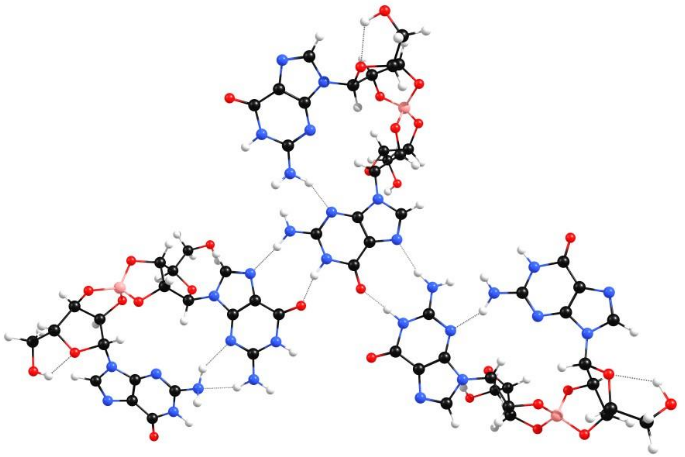

Figure S25. Optimized geometry of **Aβ-G3**, formed by three borate-guanosine diesters with two β-guanosines in anti conformation.

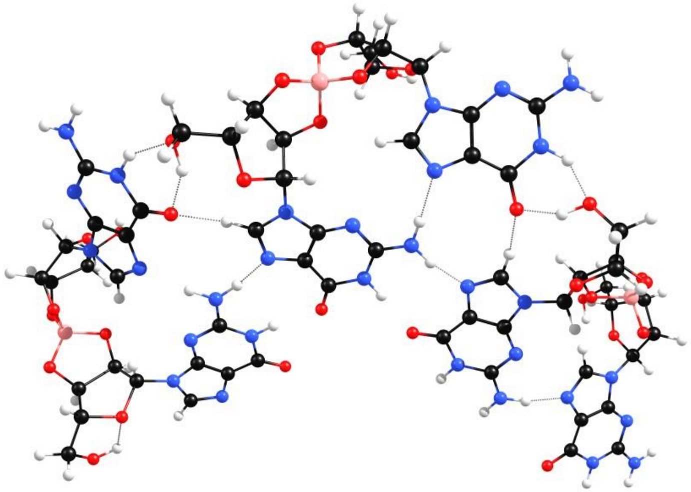

Figure S26. Optimized geometry of **Aγ-G3,** formed by three borate-guanosine diesters with one α-guanosine and one β-guanosine in anti conformation.

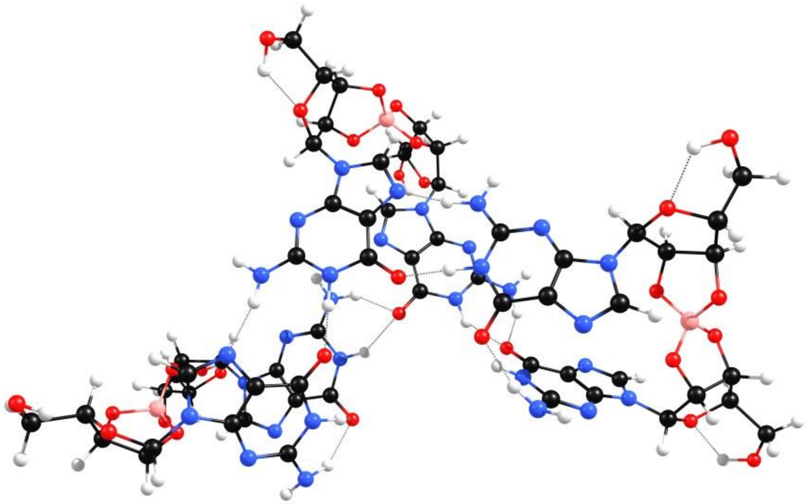

Figure S27. Optimized geometry of **Aα-G3**, formed by three borate-guanosine diesters with two α-guanosines in anti conformation.

Table S13. Optimized xyz cartesian coordinates for G3 structures in anti conformation.

	**Aβ-G3**				**Aγ-G3**				**Aα-G3**			
**Number**	**Atom**	**x**	**y**	**z**	**Atom**	**x**	**y**	**z**	**Atom**	**x**	**y**	**z**
1	C	-1.153567	4.883551	4.770876	C	1.064773	-11.624497	-1.583541	B	-1.296182	-3.587130	-1.134236
2	C	-2.422340	5.767417	4.951588	C	-0.240401	-11.523135	-2.430335	O	-0.596660	-3.141804	-2.400228
3	C	1.691926	2.222930	2.744665	C	5.323260	-11.652623	-1.873926	O	-0.837944	-4.993688	-0.940246
4	C	-2.871053	5.509371	6.424326	C	-1.397578	-11.785771	-1.396384	C	-0.274163	-6.118937	-3.138212
5	C	-1.557656	5.099207	7.122384	C	-0.759129	-11.414654	-0.047062	C	0.179150	-5.294908	-1.886755
6	C	-0.376661	2.962538	3.299074	C	3.334114	-10.617009	-1.739306	C	-3.188722	-9.255036	-3.501031
7	C	-1.776644	4.104082	8.254443	C	-1.224261	-12.221593	1.157332	C	0.613185	-3.928347	-2.495669
8	C	-1.023425	1.874805	2.713570	C	3.869737	-9.343644	-1.588054	C	1.034009	-4.233934	-3.928199
9	C	-0.265616	0.876019	2.044309	C	5.281447	-9.150807	-1.554252	C	-2.486218	-7.130253	-3.683201
10	C	-2.579342	3.052836	3.641996	C	1.699553	-9.098804	-1.523754	C	2.552554	-4.325912	-4.109327

11	H	-3.520369	3.417971	4.009708	H	0.697662	-8.697870	-1.461723	C	-3.666432	-6.660252	-4.271794
12	H	-0.323096	5.479524	4.388025	H	1.673840	-12.483675	-1.869046	C	-4.716900	-7.578814	-4.521409
13	H	1.708914	0.421763	1.642260	H	6.964402	-10.360457	-1.712943	C	-2.412680	-4.910992	-3.975451
14	H	3.677511	1.580470	2.406295	H	7.120363	-12.726964	-1.974841	H	-2.017022	-3.919128	-3.848239
15	H	3.421496	3.035399	3.379994	H	5.639853	-13.651807	-2.102834	H	-0.087105	-7.187468	-3.018830
16	H	-3.187587	5.514726	4.207577	H	-0.212545	-12.282078	-3.225192	H	-5.098902	-9.624417	-4.212805
17	H	-3.607994	4.709957	6.538628	H	-1.726496	-12.833372	-1.394041	H	-2.267744	-10.856540	-2.629907
18	H	-1.004234	5.994140	7.416435	H	-0.841229	-10.335405	0.110838	H	1.021484	-5.828680	-1.429341
19	H	-0.819794	3.889102	8.754436	H	-2.209363	-11.879191	1.483228	H	1.439774	-3.477662	-1.923667
20	H	-2.472896	4.534219	8.979432	H	-1.296720	-13.279127	0.849122	H	0.578468	-3.543573	-4.641320
21	H	-1.855021	2.763400	6.866221	H	0.594656	-12.041603	1.810868	H	2.996235	-3.327287	-4.171653
22	N	1.123894	1.141592	2.117975	N	5.949606	-10.432025	-1.725073	H	2.969643	-4.840035	-3.226182
23	N	3.040992	2.302074	2.787954	N	6.112495	-12.761966	-2.016092	H	2.154943	-5.755491	-5.337660
24	N	0.957835	3.200350	3.311210	N	4.012247	-11.791916	-1.884849	N	-4.384984	-8.886979	-4.114097
25	N	-2.400736	1.946864	2.933999	N	2.837782	-8.414611	-1.447399	N	-3.101911	-10.523201	-3.092502
26	N	-1.373236	3.732011	3.877912	N	1.962232	-10.470487	-1.703512	N	-2.184771	-8.386909	-3.311831
27	O	-0.812538	4.325601	6.087377	O	0.670094	-11.822483	-0.194180	N	-3.594103	-5.261189	-4.447324
28	O	-2.122910	7.171135	4.924943	O	-0.512182	-10.222822	-2.954022	N	-1.678401	-6.021970	-3.503399
29	O	-3.355089	6.769098	6.924095	O	-2.450703	-10.900812	-1.767448	O	0.572472	-5.626823	-4.231735
30	O	-2.385835	2.894628	7.706717	O	-0.294732	-12.048974	2.267529	O	2.853372	-5.034664	-5.342797
31	O	-0.661386	-0.149440	1.454235	O	5.969194	-8.133210	-1.401853	O	-5.884637	-7.343642	-4.972953
32	B	-2.551272	7.801320	6.264530	B	-1.830770	-9.711284	-2.434555	H	-3.904832	-11.153096	-3.208378
33	C	-0.004917	9.212920	5.162205	C	-1.650375	-6.264279	-2.392909	C	-4.388798	-3.126973	0.654492
34	C	-0.863465	9.403067	6.446016	C	-2.406985	-7.467062	-1.758496	C	-3.308397	-2.543273	-0.326149
35	C	3.105749	6.523605	3.832977	C	2.024878	-5.185431	-0.272770	C	-6.265120	-5.247977	-2.635387
36	C	-2.183982	10.079252	5.893379	C	-3.356299	-7.946989	-2.907424	C	-2.098759	-2.124198	0.601399
37	C	-1.898099	10.356910	4.393849	C	-3.354604	-6.814067	-3.955311	C	-2.424759	-2.719888	1.982033
38	C	2.346817	8.400855	4.808400	C	-0.132272	-4.934127	-0.852518	C	-5.312171	-5.099292	-0.620242
39	C	-2.258301	11.771648	3.953351	C	-4.736203	-6.246542	-4.273719	C	-2.192922	-1.803209	3.177565
40	C	3.600881	9.007611	4.931665	C	-0.309209	-3.752280	-0.134378	C	-5.374893	-6.472409	-0.365199

41	C	4.768675	8.327782	4.459786	C	0.763318	-3.183135	0.598220	C	-6.024491	-7.327935	-1.295354
42	C	2.171801	10.434044	5.699700	C	-2.202100	-4.130285	-1.126182	C	-4.286333	-5.611176	1.295890
43	H	1.691145	11.306131	6.106650	H	-3.194213	-4.007734	-1.516777	H	-3.734735	-5.446857	2.201250
44	H	-0.254254	8.240933	4.734846	H	-0.713599	-6.625886	-2.827878	H	-5.340422	-2.599129	0.552740
45	H	5.176826	6.473488	3.617493	H	2.747129	-3.656736	0.972542	H	-6.748369	-7.162215	-3.230495
46	H	3.567868	5.007245	2.512122	H	4.023171	-5.537412	0.159620	H	-6.447414	-5.550227	-4.609528
47	H	2.063936	4.787570	3.362941	H	3.164514	-6.775049	-0.816884	H	-6.054285	-3.943670	-4.121208
48	H	-0.337564	10.021293	7.181982	H	-2.958230	-7.123079	-0.877291	H	-3.746385	-1.671690	-0.830282
49	H	-2.414172	11.016984	6.408015	H	-4.371399	-8.129739	-2.538563	H	-2.019118	-1.028624	0.673368
50	H	-2.366171	9.566281	3.802388	H	-2.870871	-7.205945	-4.857276	H	-1.909361	-3.677391	2.089733
51	H	-2.084307	11.882014	2.871739	H	-4.624543	-5.377423	-4.937600	H	-1.124253	-1.718356	3.391184
52	H	-3.310276	11.975843	4.170873	H	-5.331026	-7.013270	-4.779549	H	-2.585841	-0.805146	2.919953
53	H	-0.563246	12.231437	4.737266	H	-4.949559	-5.088536	-2.641175	H	-3.720638	-2.667052	4.004192
54	N	4.386678	7.047624	3.903055	N	1.936714	-4.007389	0.465979	N	-6.485846	-6.597187	-2.412729
55	N	2.973642	5.276373	3.290306	N	3.190453	-5.855888	-0.317113	N	-6.541669	-4.807383	-3.893618
56	N	2.048369	7.183810	4.278390	N	0.984436	-5.681527	-0.948934	N	-5.773241	-4.434091	-1.701400
57	N	3.470836	10.281973	5.500864	N	-1.601827	-3.274480	-0.316082	N	-4.710520	-6.777683	0.825584
58	N	1.433079	9.310947	5.301403	N	-1.342096	-5.175187	-1.477831	N	-4.642119	-4.555042	0.453642
59	O	-0.415015	10.307607	4.255908	O	-2.570543	-5.696472	-3.381036	O	-3.906001	-2.927447	2.009587
60	O	-1.283403	8.154792	7.011922	O	-1.538350	-8.577798	-1.469385	O	-2.773766	-3.474801	-1.252417
61	O	-3.226425	9.098984	6.064502	O	-2.729429	-9.133402	-3.444090	O	-0.933806	-2.709712	0.029092
62	O	-1.447234	12.705976	4.731341	O	-5.441375	-5.891715	-3.044548	O	-2.843489	-2.348423	4.363674
63	O	5.963372	8.652668	4.459650	O	0.804817	-2.137537	1.274067	O	-6.204807	-8.565073	-1.260045
64	C	8.346092	-1.238810	1.336583	C	-9.306021	-0.395657	0.629507	B	-2.001594	-17.328745	-0.739319
65	C	9.278300	-0.082901	0.886573	C	-9.651714	1.125078	0.607132	O	-2.333967	-17.592645	0.686078
66	C	4.746907	-3.305013	-0.185799	C	-8.487986	-3.775290	-1.857950	O	-1.259916	-15.991933	-0.693270
67	C	9.909639	0.467864	2.210234	C	-9.963665	1.476168	2.109370	C	-1.965217	-14.443427	1.228755
68	C	9.753001	-0.692644	3.223728	C	-9.206690	0.387940	2.891005	C	-1.054193	-15.590929	0.659334
69	C	5.870164	-1.572872	0.750859	C	-7.903545	-1.892029	-0.776715	C	-3.972533	-10.727239	0.403749
70	C	9.294017	-0.213636	4.598647	C	-9.856048	-0.065111	4.191432	C	-1.431058	-16.841443	1.524555

71	C	4.714513	-0.846063	1.039232	C	-6.537538	-2.033116	-1.031006	C	-2.109496	-16.290304	2.780664
72	C	3.447544	-1.352619	0.651671	C	-6.095586	-3.164401	-1.758090	C	-3.789911	-12.967870	0.378311
73	C	6.352775	0.278678	1.896420	C	-6.742642	-0.221938	0.168573	C	-1.315186	-16.491585	4.072689
74	H	6.947154	0.936991	2.507031	H	-6.557113	0.678584	0.736755	C	-5.059763	-13.173582	-0.143473
75	H	8.487304	-2.108551	0.693640	H	-10.060480	-0.994048	0.115508	C	-5.867647	-12.054555	-0.487988
76	H	2.662570	-3.045347	-0.215097	H	-6.834492	-4.861054	-2.663291	C	-4.224826	-15.160270	0.175728
77	H	3.820673	-4.991611	-0.956533	H	-9.124056	-5.559208	-2.729684	H	-4.050888	-16.218499	0.212368
78	H	5.594794	-5.040512	-0.825197	H	-10.365926	-4.543429	-2.025189	H	-1.480687	-13.466515	1.216850
79	H	8.703768	0.667252	0.330024	H	-10.521097	1.283881	-0.046689	H	-5.690725	-9.961949	-0.505867
80	H	9.423830	1.375695	2.578871	H	-11.038231	1.437578	2.333281	H	-2.572390	-9.459002	1.081520
81	H	10.677913	-1.269170	3.241924	H	-8.163591	0.691089	3.019140	H	-0.016541	-15.284458	0.839540
82	H	9.349147	-1.030954	5.331288	H	-9.691049	0.678841	4.974951	H	-0.533536	-17.425140	1.780942
83	H	9.924450	0.612706	4.934609	H	-10.941446	-0.159410	4.010695	H	-3.130440	-16.670690	2.861176
84	H	7.453028	-0.475775	4.060012	H	-9.141359	-1.821991	3.777912	H	-1.416286	-17.519637	4.433492
85	N	3.562054	-2.633810	0.037637	N	-7.169108	-4.006969	-2.161601	H	-0.251554	-16.298813	3.849591
86	N	4.697756	-4.543400	-0.725100	N	-9.403612	-4.688403	-2.299726	H	-2.006691	-14.761180	4.586905
87	N	5.935340	-2.771967	0.116001	N	-8.905317	-2.724045	-1.168013	N	-5.222091	-10.833294	-0.170954
88	N	5.032623	0.310437	1.757215	N	-5.832826	-0.983586	-0.426741	N	-3.518151	-9.497469	0.721062
89	N	6.917411	-0.847549	1.278017	N	-8.041757	-0.745539	-0.023211	N	-3.200403	-11.793643	0.676854
90	O	8.641423	-1.559754	2.727719	O	-9.305799	-0.822196	2.024971	N	-5.311735	-14.545231	-0.259557
91	O	10.402325	-0.554234	0.125238	O	-8.564600	1.983191	0.255436	N	-3.243729	-14.236028	0.572182
92	O	11.298164	0.704302	1.918188	O	-9.423836	2.777533	2.319938	O	-2.123888	-14.802642	2.641136
93	O	7.923912	0.297863	4.483904	O	-9.276786	-1.326946	4.635906	O	-1.822504	-15.599807	5.104224
94	O	2.312469	-0.861093	0.787119	O	-4.924450	-3.523410	-2.066643	O	-7.012755	-12.039689	-0.998194
95	B	11.670700	-0.313306	0.919148	B	-8.262739	2.934507	1.381108	H	-4.069733	-8.624852	0.717501
96	C	12.324219	-2.771575	-0.609583	C	-4.897203	3.817931	1.394568	C	-3.145455	-17.025229	-4.078325
97	C	13.016415	-2.212801	0.680457	C	-6.165788	3.699629	2.289834	C	-3.097168	-17.927486	-2.797262
98	C	8.797278	-5.214839	-1.133666	C	-2.589275	0.133520	1.029765	C	-6.359629	-14.605325	-5.661028
99	C	13.812150	-1.004934	0.108885	C	-7.046735	4.914852	1.835710	C	-1.639457	-18.517824	-2.752820
100	C	14.232477	-1.443184	-1.284264	C	-6.119137	5.813023	0.989795	C	-0.844732	-17.616238	-3.714480

101	C	10.995447	-4.908879	-0.805809	C	-2.991723	2.206213	1.807703	C	-4.522223	-14.964854	-4.420398
102	C	15.571246	-2.251495	-1.258525	C	-6.023940	7.259485	1.470165	C	0.205393	-18.319824	-4.565195
103	C	11.336325	-6.255023	-0.965235	C	-1.771090	2.320270	2.469907	C	-4.464107	-13.661274	-3.935333
104	C	10.310583	-7.231374	-1.206122	C	-0.820233	1.262703	2.412999	C	-5.446698	-12.721671	-4.337916
105	C	13.204733	-5.183880	-0.751622	C	-2.790112	4.221139	2.766828	C	-2.841823	-14.714534	-2.953294
106	H	14.239110	-4.884368	-0.815096	H	-2.989345	5.247329	3.007061	H	-2.064816	-15.006883	-2.264199
107	H	11.330557	-2.328516	-0.699566	H	-5.052985	3.213220	0.495369	H	-3.891676	-17.368177	-4.797520
108	H	8.227571	-7.178560	-1.408068	H	-0.683370	-0.628793	1.529024	H	-7.244654	-12.747347	-5.442722
109	H	7.188326	-4.734648	-2.292932	H	-2.379593	-1.787959	0.196474	H	-8.096404	-14.288362	-6.711299
110	H	7.263295	-3.895330	-0.816214	H	-3.931272	-0.923022	-0.023521	H	-7.389065	-15.928079	-6.801725
111	H	13.625678	-2.980754	1.165849	H	-5.873460	3.763518	3.342930	H	-3.860694	-18.710611	-2.883321
112	H	14.680139	-0.738872	0.729492	H	-7.446021	5.463714	2.696591	H	-1.604578	-19.563262	-3.084987
113	H	14.255355	-0.604310	-1.979778	H	-6.482928	5.763392	-0.043383	H	-0.425496	-16.769189	-3.162627
114	H	16.379063	-1.636995	-1.675539	H	-5.304093	7.803314	0.841700	H	1.084782	-18.565949	-3.963776
115	H	15.830761	-2.487660	-0.217329	H	-7.007265	7.731875	1.387749	H	-0.243196	-19.254068	-4.944051
116	H	14.471819	-3.449943	-2.312065	H	-4.692317	6.917250	2.933174	H	-0.225814	-17.014630	-5.932791
117	N	9.027987	-6.563071	-1.284112	N	-1.328597	0.167891	1.637491	N	-6.402680	-13.295125	-5.195155
118	N	7.457682	-4.792654	-1.302647	N	-2.968829	-0.946522	0.352193	N	-7.335397	-14.958345	-6.520569
119	N	9.749478	-4.351756	-0.869915	N	-3.454040	1.168535	1.102308	N	-5.412775	-15.478449	-5.298569
120	N	12.724130	-6.409281	-0.924882	N	-1.671866	3.579780	3.061500	N	-3.399454	-13.518746	-3.032839
121	N	12.182163	-4.224683	-0.656868	N	-3.642416	3.417269	2.002282	N	-3.484083	-15.643201	-3.803202
122	O	13.169901	-2.374459	-1.761638	O	-4.757397	5.253111	1.102052	O	-1.832835	-17.154607	-4.723039
123	O	12.078417	-1.621187	1.582517	O	-6.941548	2.520863	1.998135	O	-3.233076	-17.210177	-1.569070
124	O	12.822275	0.041501	0.060956	O	-8.082120	4.344458	1.003134	O	-1.200073	-18.395075	-1.392014
125	O	15.435482	-3.493948	-2.013562	O	-5.638977	7.299861	2.879815	O	0.631824	-17.448074	-5.653032
126	O	10.368033	-8.457337	-1.354271	O	0.320835	1.219588	2.922547	O	-5.520133	-11.506614	-3.998607
127	C	-7.296677	-3.842324	-0.482256	C	0.595377	11.113792	8.020253	O	-14.979697	-17.024930	-4.622637
128	C	-8.092436	-4.653672	-1.549532	C	-0.740400	10.309640	7.947922	B	-14.213631	-16.374556	-5.749290
129	C	-4.117299	-1.136545	0.923698	H	-1.611760	10.967384	7.939842	O	-12.813275	-16.819529	-5.535649
130	C	-8.484564	-5.977147	-0.825428	N	-0.885463	9.482199	6.750347	O	-14.443010	-14.897524	-5.670968

131	C	-8.535142	-5.556487	0.658290	O	-0.827631	9.502552	9.162422	O	-14.719839	-16.747225	-7.109466
132	C	-4.946362	-2.873427	-0.271948	C	1.166822	10.799165	9.451901	C	-14.270849	-18.232700	-4.279727
133	C	-8.145177	-6.687896	1.602381	H	0.377198	12.182836	7.883323	C	-12.776934	-17.864618	-4.559278
134	C	-3.689096	-3.320612	-0.684207	O	1.613378	10.662650	7.126635	C	-12.203384	-17.462525	-3.147569
135	C	-2.507293	-2.602257	-0.305167	N	-4.139109	8.812631	4.526212	H	-11.201410	-17.861988	-2.979252
136	C	-5.102739	-4.760426	-1.452266	C	-4.209981	9.792930	5.483299	N	-12.086683	-16.041647	-2.924127
137	H	-5.565339	-5.608783	-1.923650	N	-5.396854	10.460301	5.608964	O	-13.094965	-18.089841	-2.155956
138	H	-7.697730	-2.831911	-0.387366	N	-3.199123	10.118443	6.276746	H	-12.202792	-18.725127	-4.922540
139	H	-2.037406	-0.954220	0.910452	C	0.519849	9.445497	9.797094	N	-8.976886	-13.795614	-1.763865
140	H	-3.521007	0.513866	2.133820	H	0.890660	11.562445	10.191837	C	-8.802245	-15.171448	-1.767516
141	H	-5.196650	-0.011394	2.199280	O	2.580956	10.703929	9.290694	N	-7.622751	-15.676481	-1.377302
142	H	-7.486518	-4.806383	-2.449976	C	0.198100	9.225223	11.268506	N	-9.772198	-16.023771	-2.157824
143	H	-7.762655	-6.789247	-0.947233	H	1.116899	8.636158	9.368122	C	-14.358954	-18.553169	-2.795171
144	H	-9.506035	-5.107014	0.878267	C	-1.897926	8.365389	5.119744	H	-14.587775	-19.090190	-4.895241
145	H	-8.262598	-6.360648	2.646963	C	-2.076096	9.381355	6.060011	C	-14.380179	-20.057407	-2.496916
146	H	-8.794911	-7.548468	1.419023	H	1.112624	9.005328	11.825554	H	-15.199682	-18.033080	-2.331114
147	H	-6.333377	-6.193997	1.160016	H	-0.242204	10.159450	11.660027	C	-11.219740	-14.055195	-2.436771
148	N	-2.850498	-1.487936	0.540245	O	-0.711829	8.097551	11.426610	C	-10.930054	-15.420604	-2.472208
149	N	-4.275372	-0.097832	1.783536	C	-2.986379	8.012087	4.283847	H	-15.379616	-20.471610	-2.665991
150	N	-5.217862	-1.780439	0.489175	N	-0.607498	7.830629	5.233297	O	-14.023798	-20.281784	-1.104986
151	N	-3.808753	-4.504273	-1.423862	O	-3.071482	7.111130	3.407178	C	-10.170275	-13.146786	-2.147850
152	N	-5.864607	-3.780326	-0.781402	H	0.989572	8.370402	6.575180	N	-12.557938	-13.832123	-2.820530
153	O	-7.438746	-4.559679	0.801459	C	-0.015809	8.508526	6.208494	O	-10.187969	-11.877430	-2.258740
154	O	-9.360564	-4.060458	-1.879565	H	-4.949773	8.522870	3.928459	H	-14.025875	-15.249032	-3.481353
155	O	-9.792411	-6.333512	-1.315588	H	-6.225497	10.166932	5.110307	C	-13.044515	-15.022966	-3.108510
156	O	-6.769805	-7.085789	1.310653	H	-5.465899	11.139992	6.354232	H	-8.179722	-13.164879	-1.494686
157	O	-1.321118	-2.830246	-0.583856	H	-1.325034	8.197539	10.643223	H	-7.549930	-16.686010	-1.443257
158	B	-10.481759	-5.063159	-1.561027	B	2.834490	10.221860	7.894556	H	-13.310782	-19.587315	-0.967148
159	C	-11.420644	-2.159139	-1.051257	O	2.915736	8.714119	7.783191	H	-6.794277	-15.153408	-0.997578
160	C	-12.113296	-3.542778	-0.883625	O	4.124350	10.721646	7.390505	C	-15.308120	-14.480879	-6.715814

161	C	-8.562216	0.286604	1.068400	C	4.266160	8.311120	7.482876	C	-14.612128	-13.607073	-7.821953
162	C	-12.390162	-3.983539	-2.379501	C	4.360579	7.572535	6.118566	H	-14.988708	-12.580086	-7.802412
163	C	-12.020768	-2.745961	-3.238827	H	3.461885	7.818038	5.545122	N	-13.164046	-13.565646	-7.694076
164	C	-10.641171	-0.420795	0.582964	N	4.466026	6.138058	6.127043	O	-14.916526	-14.206608	-9.122005
165	C	-13.093079	-2.354121	-4.251204	O	5.580677	8.110152	5.464958	C	-15.734863	-15.796207	-7.463184
166	C	-11.301069	0.573221	1.312763	C	5.064997	9.643417	7.252083	H	-16.174456	-13.927132	-6.333695
167	C	-10.539403	1.562280	2.011953	H	4.701673	7.692963	8.276648	N	-10.690580	-11.896529	-5.062150
168	C	-12.853454	-0.606672	0.382091	N	1.967494	3.468370	4.510512	C	-12.054292	-11.686416	-4.879769
169	H	-13.793947	-1.001733	0.040209	C	1.521153	4.781710	4.626283	N	-12.396487	-11.023012	-3.746208
170	H	-10.349673	-2.334471	-1.162488	N	0.299683	5.116986	4.223608	N	-12.971225	-12.116155	-5.745438
171	H	-8.526016	1.949455	2.327153	N	2.301942	5.751426	5.153169	C	-15.715648	-15.464202	-8.946062
172	H	-6.655464	1.073457	1.106188	C	5.599171	9.552676	5.803060	H	-16.741995	-16.113414	-7.144879
173	H	-6.714045	-0.615855	0.666670	H	5.912071	9.726451	7.943612	C	-17.094128	-15.107741	-9.515704
174	H	-13.027761	-3.455048	-0.286456	C	7.068327	9.948301	5.663843	H	-15.221221	-16.247403	-9.521946
175	H	-13.438331	-4.253251	-2.543723	H	4.931248	10.128829	5.154864	C	-11.109259	-13.159220	-6.986807
176	H	-11.031310	-2.912869	-3.671749	C	4.021800	4.027981	5.561064	C	-12.459898	-12.887341	-6.725750
177	H	-12.737615	-1.507398	-4.858996	C	3.491382	5.316866	5.573621	H	-17.676608	-16.014848	-9.704347
178	H	-13.316908	-3.199294	-4.908296	H	7.355908	9.941317	4.600638	H	-17.632322	-14.500146	-8.766779
179	H	-13.920691	-1.516942	-2.734345	H	7.223128	10.951452	6.071817	O	-16.932346	-14.397643	-10.775054
180	N	-9.129161	1.319043	1.803517	O	7.865422	9.010596	6.448132	C	-10.126449	-12.623276	-6.121268
181	N	-7.205305	0.224755	1.030531	C	3.239785	2.965879	4.983846	N	-10.980466	-14.005152	-8.097512
182	N	-9.304330	-0.611982	0.434004	N	5.320592	4.038546	6.110051	O	-8.870476	-12.759169	-6.208433
183	N	-12.690640	0.434384	1.180654	O	3.522863	1.765840	4.851425	H	-12.533796	-14.856414	-9.307935
184	N	-11.633853	-1.177302	-0.011878	H	6.465540	5.685181	6.862406	C	-12.220055	-14.239101	-8.488614
185	O	-11.995197	-1.595658	-2.293712	C	5.558646	5.298115	6.430655	H	-10.084712	-11.661227	-4.264150
186	O	-11.230517	-4.538930	-0.353124	H	1.373840	2.761372	4.046319	H	-11.700164	-11.129031	-2.981842
187	O	-11.504855	-5.093989	-2.629919	H	-0.390681	4.446658	3.810420	H	-13.360902	-11.192989	-3.472103
188	O	-14.307774	-2.020936	-3.511807	H	0.015421	6.086537	4.429819	H	-16.119847	-13.839056	-10.584347
189	O	-10.889594	2.521649	2.712679	H	7.387033	8.148123	6.257967	H	-13.676404	-20.554587	-3.187624

S3.2. Syn Conformation

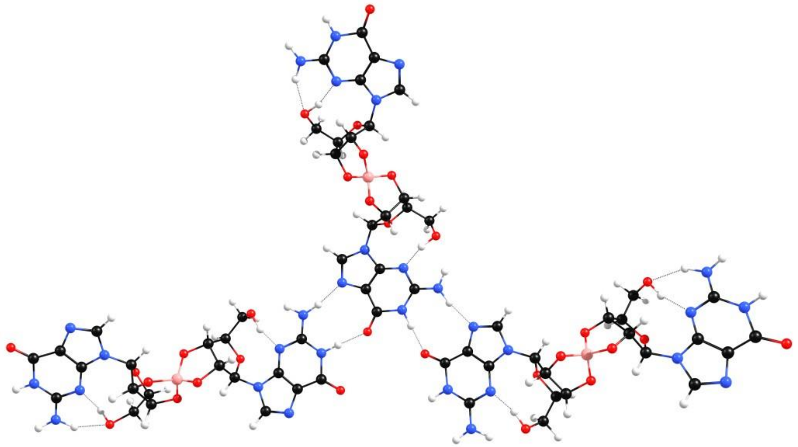

Figure S28. Optimized geometry of **Sβ-G3**, formed by three borate-guanosine diesters with two β-guanosines in syn conformation.

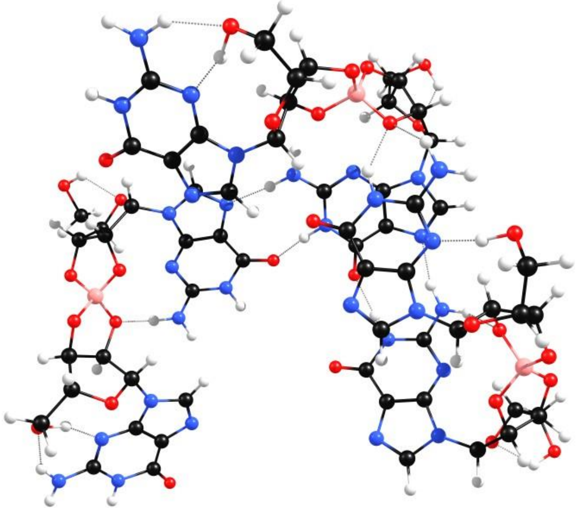

Figure S29. Optimized geometry of **Sγ-G3**, formed by three borate-guanosine diesters with one α-guanosine and one β-guanosine in syn conformation.

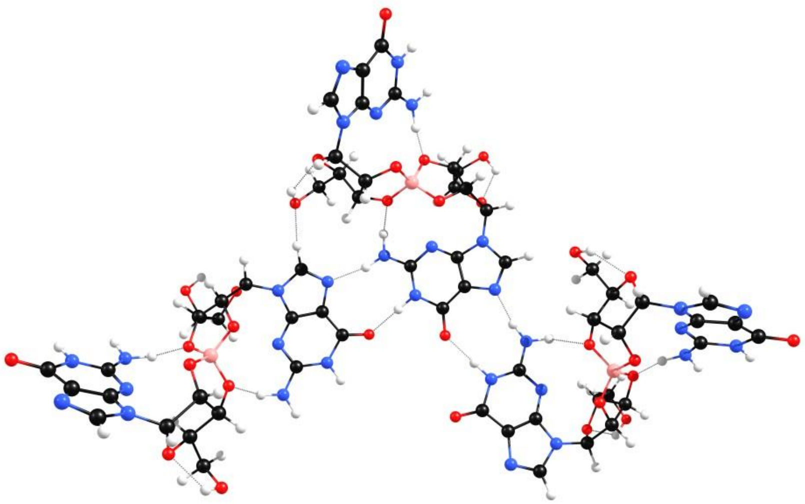

Figure S30. Optimized geometry of **Sα-G3**, formed by three borate-guanosine diesters with two α-guanosines in syn conformation.

Table S14. Optimized xyz cartesian coordinates for G3 structures in syn conformation.

	**Sβ-G3**				**Sγ-G3**				**Sα-G3**			
**Number**	**Atom**	**x**	**y**	**z**	**Atom**	**x**	**y**	**z**	**Atom**	**x**	**y**	**z**
1	O	-12.966282	8.082735	-4.723636	O	-3.64882	1.15187	1.08009	C	4.053278	-4.778694	1.872310
2	O	-15.284104	7.690903	-4.420908	O	-2.95454	2.89669	-0.37310	C	4.778793	-3.593025	1.129667
3	B	-13.969664	7.415723	-3.803159	B	-2.94470	1.37362	-0.20436	C	0.995871	-4.278758	-1.179635
4	C	-11.326251	6.330946	-2.940337	C	-2.16684	0.07331	-3.30766	C	5.684598	-4.310431	0.084515
5	C	-12.772390	5.912592	-2.513853	C	-2.70465	-0.32921	-1.90192	C	4.962925	-5.643524	-0.168839
6	C	-9.737468	3.534362	-0.035678	C	-1.76910	-4.15442	-4.18779	C	1.702446	-4.589119	0.929647
7	C	-13.183177	7.066734	-1.545332	C	-1.44707	-0.33781	-0.97653	C	5.876658	-6.803322	-0.557693
8	C	-11.862837	7.632945	-0.986488	C	-0.22531	-0.30743	-1.91933	C	0.430650	-4.533302	1.514029
9	C	-9.973203	4.357153	-2.141083	C	-2.58913	-2.06922	-4.57089	C	-0.720811	-4.385423	0.676952
10	C	-11.558310	7.215648	0.458561	C	0.62574	-1.58524	-1.85821	C	1.832630	-4.697727	3.150234

11	C	-9.086003	3.454626	-2.718646	C	-3.37978	-2.44006	-5.66143	H	2.302191	-4.767309	4.117452
12	C	-8.442675	2.481783	-1.903104	C	-3.46389	-3.81381	-6.03178	H	4.423959	-4.854131	2.898437
13	C	-9.787282	4.749051	-4.311550	C	-3.63738	-0.29611	-5.41338	H	-1.078049	-4.022945	-1.336305
14	H	-9.968573	5.222806	-5.259744	H	-3.92135	0.74363	-5.47811	H	0.572430	-3.638791	-3.092367
15	H	-11.386656	6.898567	-3.873866	H	-2.42868	1.11109	-3.51919	H	2.270640	-3.561342	-2.521347
16	H	-8.388761	1.933928	0.095265	H	-2.52380	-5.59801	-5.49826	H	5.371238	-3.040564	1.872708
17	H	-9.717435	2.969939	1.972932	H	-0.86462	-5.95711	-3.63769	H	6.703875	-4.489964	0.442454
18	H	-10.696878	4.393053	1.510935	H	-0.36281	-4.47370	-2.77344	H	4.127818	-5.483122	-0.849992
19	H	-12.774648	4.926193	-2.045674	H	-3.18769	-1.31013	-1.95125	H	5.278507	-7.672876	-0.862936
20	H	-13.828043	6.712410	-0.733360	H	-1.41826	-1.23590	-0.35036	H	6.525160	-6.500934	-1.383643
21	H	-11.884091	8.723474	-1.074771	H	0.37283	0.57555	-1.67172	H	6.104571	-6.925241	1.362441
22	H	-10.502073	7.451833	0.660524	H	1.29429	-1.58455	-2.73315	N	-0.307727	-4.235517	-0.706790
23	H	-12.184460	7.807576	1.135366	H	1.23637	-1.56095	-0.94828	N	1.295544	-3.974298	-2.465683
24	H	-11.406038	5.300797	-0.058273	H	-0.92813	-2.64586	-2.52858	N	2.009454	-4.570949	-0.375999
25	N	-8.838883	2.610847	-0.519836	N	-2.53837	-4.61759	-5.22731	N	0.537889	-4.608339	2.913396
26	N	-10.046325	3.624291	1.276950	N	-0.91384	-4.95843	-3.50114	N	2.603558	-4.680085	1.969278
27	N	-10.337239	4.402064	-0.846446	N	-1.82146	-2.88464	-3.81218	O	4.449235	-6.019080	1.187589
28	N	-8.984216	3.713727	-4.089500	N	-4.02509	-1.30701	-6.17869	O	3.957336	-2.715686	0.358849
29	N	-10.422586	5.178292	-3.142276	N	-2.75907	-0.72615	-4.39339	O	5.675935	-3.474245	-1.084285
30	O	-10.760626	7.108578	-1.847174	O	-0.73128	-0.19225	-3.31236	O	6.718947	-7.141018	0.596409
31	O	-13.675536	5.976276	-3.622224	O	-3.59408	0.67148	-1.37095	O	-1.931959	-4.344018	0.945106
32	O	-13.848982	8.008031	-2.416066	O	-1.54802	0.87720	-0.20787	B	4.567686	-2.447911	-0.984713
33	O	-11.868830	5.810277	0.709969	O	-0.20612	-2.78201	-1.79375	O	3.563850	-2.604337	-2.125053
34	O	-7.624146	1.594175	-2.197643	O	-4.16979	-4.41380	-6.86009	O	5.041644	-1.042358	-1.136894
35	C	-15.188481	10.155959	-4.231985	C	-4.67407	4.59568	0.61821	C	5.121422	0.500317	-3.197819
36	C	-15.127648	8.903235	-5.165841	C	-3.52157	3.53600	0.79272	C	4.303808	-0.330766	-2.133009
37	C	-16.967943	12.355592	-7.480570	C	-6.22580	2.44466	-2.91440	C	8.494417	-2.106056	-2.167105
38	C	-13.651271	8.926294	-5.671888	C	-4.17924	2.37045	1.59734	C	3.634412	-1.445421	-2.980603
39	C	-13.190382	10.392007	-5.560069	C	-5.68217	2.54186	1.33033	C	4.608631	-1.636927	-4.150952
40	C	-16.969703	11.530570	-5.363214	C	-5.59960	4.26526	-1.73712	C	7.520436	-0.135865	-2.644179

41	C	-13.107132	11.128939	-6.903914	C	-6.55365	2.09108	2.49645	C	3.925799	-2.135645	-5.420385
42	C	-18.167234	12.137488	-4.980299	C	-6.06563	5.16911	-2.68794	C	8.682342	0.611592	-2.456553
43	C	-18.879460	12.945105	-5.922813	C	-6.74617	4.68849	-3.84929	C	9.901132	-0.051381	-2.127117
44	C	-17.422232	11.074496	-3.247136	C	-5.19272	6.37857	-1.11637	C	7.110729	2.058016	-2.881407
45	H	-17.284708	10.643440	-2.270848	H	-4.83147	7.19029	-0.50822	H	6.555160	2.970245	-3.064387
46	H	-14.995716	9.834607	-3.203719	H	-4.40322	5.50456	1.16217	H	4.641566	1.470486	-3.351356
47	H	-18.597948	13.594698	-7.887439	H	-7.31023	2.86084	-4.67938	H	10.522892	-1.986578	-1.624963
48	H	-16.728049	12.976929	-9.460304	H	-5.51957	0.67243	-2.43505	H	7.347980	-3.645865	-1.794773
49	H	-15.413687	12.010352	-8.719598	H	-2.72217	4.02151	1.36828	H	3.564566	0.342765	-1.674837
50	H	-15.864426	8.977458	-5.968483	H	-4.01564	2.47336	2.67638	H	2.652915	-1.172713	-3.381581
51	H	-13.564472	8.564489	-6.702614	H	-5.94707	2.12358	0.35907	H	5.483070	-2.208250	-3.837057
52	H	-12.224488	10.413414	-5.046415	H	-7.61682	2.18017	2.22743	H	4.674569	-2.305688	-6.208369
53	H	-13.044923	12.207573	-6.691794	H	-6.32740	1.04815	2.73564	H	3.406802	-3.076375	-5.211881
54	H	-12.192411	10.819897	-7.421030	H	-6.10784	3.81100	3.23672	H	3.428608	-0.291085	-5.635344
55	H	-15.065877	10.914813	-7.187634	N	-6.81234	3.26428	-3.85897	N	9.700038	-1.454111	-1.962463
56	N	-18.149446	12.996424	-7.197535	N	-6.19018	1.11543	-3.07191	N	8.352947	-3.415274	-1.894955
57	N	-16.329729	12.493411	-8.668739	N	-5.67251	2.93500	-1.78402	N	7.403048	-1.463953	-2.615152
58	N	-16.381168	11.581629	-6.576652	N	-5.79363	6.48162	-2.28462	N	8.405823	1.975584	-2.625280
59	N	-18.438218	11.831192	-3.636960	N	-5.02559	5.03004	-0.72334	N	6.508655	0.782083	-2.887870
60	N	-16.489972	10.852700	-4.275146	O	-5.84372	4.03149	1.31234	O	4.998644	-0.233120	-4.474802
61	O	-14.194869	11.105315	-4.716873	O	-6.22372	2.91005	3.66530	O	2.929068	-1.142683	-5.824747
62	O	-14.228063	10.800131	-7.782881	O	-7.25987	5.35261	-4.77311	O	11.042245	0.436960	-1.956523
63	O	-19.936265	13.580308	-5.840087	H	-6.54291	0.61507	-3.90973	H	9.032862	-3.958730	-1.339568
64	O	-5.567502	-3.488600	-11.601571	O	-3.58990	12.67932	-4.45811	C	14.764159	-6.072052	7.714299
65	O	-7.656295	-3.505066	-12.723785	O	-3.06059	11.18376	-6.23280	C	13.890414	-5.848443	6.421254
66	B	-6.920017	-2.801834	-11.645039	B	-2.97206	11.36202	-4.72130	C	14.347660	-10.308175	6.813796
67	C	-5.748730	-1.751245	-9.087397	C	-2.16753	8.49718	-5.08373	C	14.943948	-5.724783	5.284648
68	C	-7.194125	-1.618873	-9.670094	C	-2.61833	9.23532	-3.77458	C	16.145056	-6.522369	5.810464
69	C	-7.050781	0.749232	-5.760075	C	-2.01686	5.02302	-2.49425	C	14.346990	-8.533477	8.190597
70	C	-7.010807	-0.544737	-10.783291	C	-1.36328	10.07618	-3.44330	C	17.487531	-6.011344	5.302296

71	C	-5.800917	0.300256	-10.348615	C	-0.17648	9.25026	-3.94661	C	13.943597	-9.240384	9.330241
72	C	-6.155363	-1.138481	-6.666395	C	-2.58356	6.10716	-4.39935	C	13.760875	-10.657867	9.249956
73	C	-6.191045	1.654945	-9.743013	C	0.47932	8.41161	-2.83629	C	14.021677	-7.161510	9.913703
74	C	-5.910535	-1.742738	-5.437329	C	-3.19719	4.99721	-4.98637	H	13.976027	-6.226318	10.446622
75	C	-6.266922	-1.061936	-4.234849	C	-3.25112	3.76816	-4.25861	H	14.704148	-5.188446	8.356476
76	C	-5.216157	-3.127079	-6.959712	C	-3.43127	6.63710	-6.38148	H	13.757187	-12.089417	7.745379
77	H	-4.811037	-3.972218	-7.487254	H	-3.70678	7.26602	-7.21040	H	14.040431	-11.726641	5.347706
78	H	-5.242653	-2.576180	-9.598107	H	-2.44696	9.10876	-5.94617	H	14.177024	-10.010180	4.863236
79	H	-7.112087	0.765441	-3.663480	H	-2.90899	3.19647	-2.24598	H	13.316053	-4.920201	6.553194
80	H	-7.998041	2.569353	-5.218058	H	-1.55462	4.13809	-0.72407	H	15.257709	-4.695938	5.084539
81	H	-7.630175	2.247214	-6.936018	H	-0.86691	5.80084	-1.02786	H	15.977255	-7.590410	5.677272
82	H	-7.905164	-1.329430	-8.895143	H	-2.91127	8.53091	-2.99776	H	18.298231	-6.652049	5.681597
83	H	-7.901440	0.081827	-10.897769	H	-1.27813	10.28756	-2.37095	H	17.499512	-6.034385	4.208712
84	H	-5.127352	0.432961	-11.201390	H	0.55590	9.91026	-4.42389	H	17.300560	-4.661339	6.665669
85	H	-5.310739	2.045342	-9.207165	H	1.08099	7.62920	-3.32854	N	13.972287	-11.104735	7.884233
86	H	-6.437195	2.346125	-10.557983	H	1.15592	9.05820	-2.26145	N	14.374860	-10.790215	5.545772
87	H	-7.133922	0.827043	-8.193162	H	-1.13260	7.28967	-2.47901	N	14.648252	-9.027985	6.979737
88	N	-6.843640	0.222750	-4.504713	N	-2.67754	3.91207	-2.95265	N	13.749741	-8.355540	10.404410
89	N	-7.570553	1.973183	-5.946584	N	-1.39159	4.96901	-1.29999	N	14.385330	-7.203473	8.551452
90	N	-6.725633	0.059499	-6.867621	N	-1.95497	6.15075	-3.20302	O	16.157967	-6.185114	7.270431
91	N	-5.317962	-2.995193	-5.642708	N	-3.71455	5.35432	-6.24147	O	13.051809	-6.931670	6.021315
92	N	-5.725036	-2.014838	-7.636871	N	-2.73966	7.15898	-5.26484	O	14.346607	-6.343719	4.126445
93	O	-5.078975	-0.475283	-9.296655	O	-0.71500	8.31942	-4.97265	O	17.650180	-4.622639	5.726378
94	O	-7.598487	-2.824848	-10.335695	O	-3.64975	10.21315	-4.01105	O	13.445811	-11.495991	10.108412
95	O	-6.727845	-1.335223	-11.961743	O	-1.55814	11.26956	-4.24226	B	13.159423	-7.191685	4.550153
96	O	-7.367395	1.557937	-8.892982	O	-0.49043	7.86939	-1.89990	O	13.373888	-8.667619	4.239819
97	O	-6.136332	-1.434455	-3.050648	O	-3.70879	2.64936	-4.57955	O	11.905206	-6.859685	3.813682
98	C	-6.144832	-2.776069	-14.539757	C	-4.76305	12.25279	-7.85574	C	10.690728	-8.051377	1.891051
99	C	-6.653798	-3.951028	-13.640657	C	-3.54989	12.36986	-6.84685	C	11.231515	-8.040146	3.372945
100	C	-5.564605	-5.614778	-17.720082	C	-6.14148	8.81568	-5.47831	C	13.837320	-5.076856	1.157468

101	C	-5.391617	-4.291838	-12.785989	C	-4.11561	13.23381	-5.66721	C	12.327619	-9.144443	3.370298
102	C	-4.182558	-3.831832	-13.625748	C	-5.63878	13.07396	-5.78435	C	12.777059	-9.219022	1.906857
103	C	-6.484857	-3.732523	-16.842285	C	-5.70043	9.92167	-7.39322	C	11.774193	-5.947482	0.944536
104	C	-3.409156	-4.980076	-14.288714	C	-6.41421	14.30345	-5.32364	C	13.268527	-10.601088	1.495385
105	C	-7.133543	-3.336330	-18.013959	C	-6.13381	8.89090	-8.23427	C	11.288495	-4.907280	0.152691
106	C	-7.017736	-4.144016	-19.188720	C	-6.71721	7.70729	-7.65457	C	12.158569	-3.854742	-0.243895
107	C	-7.535502	-1.813898	-16.527137	C	-5.39423	10.44837	-9.54298	C	9.591285	-6.213080	0.518628
108	H	-7.881107	-0.945318	-15.994250	H	-5.09394	11.04469	-10.38972	H	8.595915	-6.645770	0.565631
109	H	-6.388372	-1.829267	-14.047477	H	-4.50518	12.75095	-8.79533	H	9.656063	-8.400762	1.865676
110	H	-6.012845	-5.889800	-19.741408	H	-6.88308	6.87481	-5.72152	H	14.078464	-3.224284	0.192325
111	H	-4.607104	-7.390687	-18.267771	H	-5.29601	9.44359	-3.82535	H	14.986221	-5.674383	2.662743
112	H	-4.312449	-6.749405	-16.619483	H	-2.74751	12.90879	-7.37787	H	10.395653	-8.296955	4.039426
113	H	-7.008013	-4.784847	-14.250625	H	-3.87575	14.29844	-5.77812	H	11.957514	-10.129059	3.673942
114	H	-5.324351	-5.362981	-12.563124	H	-5.95048	12.12675	-5.34357	H	13.457084	-8.398112	1.678318
115	H	-3.523092	-3.237599	-12.985961	H	-7.49587	14.09826	-5.34716	H	13.595826	-10.584991	0.445462
116	H	-2.751724	-4.545876	-15.057882	H	-6.11747	14.55561	-4.30121	H	14.111187	-10.895061	2.128370
117	H	-2.788579	-5.471794	-13.531881	H	-5.97520	14.94152	-7.07794	H	11.394216	-11.041370	1.377478
118	H	-5.003367	-5.465616	-15.378967	N	-6.62106	7.74993	-6.21240	N	13.453602	-4.012313	0.354599
119	N	-6.159806	-5.308080	-18.920059	N	-5.99669	8.74804	-4.14294	N	15.029874	-5.080445	1.798839
120	N	-4.743579	-6.683163	-17.560887	N	-5.76525	9.96986	-6.06125	N	13.035634	-6.114656	1.370583
121	N	-5.751992	-4.849881	-16.653871	N	-5.93253	9.24710	-9.58167	N	9.926985	-5.096746	-0.108479
122	N	-7.798312	-2.119380	-17.790658	N	-5.20819	10.92216	-8.22225	N	10.695576	-6.775536	1.190845
123	N	-6.734148	-2.778958	-15.894502	O	-5.85932	13.04622	-7.26717	O	11.490045	-9.054188	1.166135
124	O	-4.708497	-2.959603	-14.716799	O	-6.06467	15.42553	-6.20022	O	12.185472	-11.562301	1.709655
125	O	-4.302888	-5.999581	-14.838134	O	-7.23808	6.73394	-8.21773	O	11.951092	-2.865958	-0.975626
126	O	-7.480945	-4.023294	-20.328874	H	-6.10457	7.88476	-3.59332	H	15.644881	-4.275799	1.759109
127	O	-2.435102	-10.788262	1.733581	O	-7.85696	-3.81033	-11.04797	C	13.990782	7.658769	-3.431448
128	O	-3.565079	-12.441586	0.474644	O	-6.63571	-2.21758	-9.78146	C	12.642433	8.424840	-3.142899
129	B	-3.193963	-10.995084	0.438461	B	-7.21142	-2.46989	-11.12175	C	11.721438	3.946442	-2.976450
130	C	-3.107440	-7.925759	0.371816	C	-7.22167	0.45331	-12.90638	C	12.046873	8.659186	-4.555920

131	C	-3.874471	-8.970102	-0.502981	C	-7.88796	-0.95793	-12.91961	C	12.586658	7.468106	-5.359963
132	C	-4.634946	-4.763701	-2.231454	C	-8.90966	1.14079	-16.81399	C	13.486552	5.298272	-2.634358
133	C	-2.762841	-9.530793	-1.439613	C	-6.74576	-1.89621	-13.40797	C	12.711194	7.755348	-6.851092
134	C	-1.688187	-8.433846	-1.514211	C	-5.73591	-0.99087	-14.13836	C	14.157558	4.415348	-1.784091
135	C	-4.316072	-5.772687	-0.209308	C	-8.63520	1.64920	-14.61646	C	13.595993	3.111111	-1.541324
136	C	-1.748697	-7.630699	-2.822494	C	-5.71509	-1.16797	-15.66146	C	15.364800	6.213521	-1.835093
137	C	-4.920173	-4.754639	0.526081	C	-9.40736	2.81213	-14.64961	H	16.121914	6.967194	-1.688139
138	C	-5.454737	-3.608317	-0.159209	C	-10.03547	3.20847	-15.87310	H	14.831719	8.333222	-3.239338
139	C	-4.246110	-6.229968	1.962433	C	-8.61308	2.65211	-12.64161	H	11.826888	2.071479	-2.046788
140	H	-4.022730	-6.790008	2.854542	H	-8.33301	2.82674	-11.61733	H	9.805529	3.084308	-3.084204
141	H	-2.830271	-8.412020	1.312831	H	-6.89875	0.68390	-11.88783	H	10.084176	4.722017	-3.720988
142	H	-5.571061	-2.896337	-2.142733	H	-10.06333	2.53670	-17.85088	H	12.891938	9.369590	-2.634828
143	H	-4.797005	-4.079948	-4.239830	H	-8.96357	0.49035	-18.79731	H	12.376953	9.585344	-5.038277
144	H	-3.934162	-5.607204	-3.893937	H	-7.93427	-0.42097	-17.64446	H	12.057681	6.550604	-5.097479
145	H	-4.697535	-8.496873	-1.039947	H	-8.75185	-0.95954	-13.59163	H	13.055501	6.854084	-7.379163
146	H	-3.149704	-9.759485	-2.437424	H	-7.12849	-2.67517	-14.07954	H	11.737951	8.055952	-7.250572
147	H	-0.702175	-8.888767	-1.373892	H	-4.74621	-1.17627	-13.71071	H	14.328282	8.646450	-6.323131
148	H	-1.209764	-6.684158	-2.656015	H	-5.19385	-0.30024	-16.09348	N	12.315992	2.977891	-2.192926
149	H	-1.226250	-8.195043	-3.605364	H	-5.14996	-2.07497	-15.90202	N	10.463283	3.808869	-3.426694
150	H	-3.585796	-6.921747	-2.492161	H	-7.61524	-0.58351	-15.77667	N	12.353845	5.088135	-3.306407
151	N	-5.241651	-3.711792	-1.591523	N	-9.68594	2.26450	-16.94644	N	15.336029	5.011842	-1.296580
152	N	-4.446186	-4.781713	-3.560822	N	-8.59410	0.34728	-17.86906	N	14.242271	6.463105	-2.661914
153	N	-4.182436	-5.833475	-1.546871	N	-8.41220	0.78953	-15.63514	O	14.002966	7.389262	-4.885624
154	N	-4.868276	-5.064532	1.897827	N	-9.38520	3.42600	-13.38948	O	11.621799	7.711887	-2.446073
155	N	-3.889502	-6.723631	0.693279	N	-8.13710	1.52834	-13.34698	O	10.614936	8.621993	-4.386154
156	O	-1.935915	-7.498026	-0.382452	O	-6.12288	0.42336	-13.85663	O	13.646456	8.871752	-7.027313
157	O	-4.333081	-10.079350	0.292106	O	-8.22990	-1.39678	-11.59608	O	14.057324	2.174109	-0.870667
158	O	-2.291456	-10.706392	-0.738698	O	-6.17469	-2.40033	-12.19437	B	10.290372	7.845838	-3.107820
159	O	-3.113887	-7.430050	-3.277592	O	-7.05797	-1.33756	-16.21726	O	9.628137	6.527496	-3.428704
160	O	-6.025797	-2.610324	0.302317	O	-10.76633	4.16037	-16.16317	O	9.292011	8.548596	-2.238712

161	C	-1.256059	-13.278436	0.161342	C	-7.18390	-3.36206	-7.51910	C	6.789971	8.158484	-1.894767
162	C	-2.469412	-13.085380	1.129649	C	-6.65477	-3.42456	-9.00059	C	8.275535	7.643261	-1.804103
163	C	-0.014063	-16.783938	2.338513	C	-9.11943	0.33823	-8.89561	C	8.357940	10.816618	-4.974610
164	C	-1.925567	-12.056025	2.185328	C	-7.70422	-4.33119	-9.72296	C	8.324906	6.467525	-2.826168
165	C	-0.384237	-12.140784	2.097563	C	-8.97854	-4.14116	-8.88680	C	7.211534	6.813097	-3.828190
166	C	-0.700052	-15.719828	0.453355	C	-8.02813	-0.94989	-7.41171	C	7.115235	10.344012	-3.163114
167	C	0.283184	-12.802000	3.311835	C	-9.90634	-5.35197	-8.91093	C	6.551358	5.609420	-4.491770
168	C	-0.552033	-16.825745	-0.388658	C	-7.87716	0.06126	-6.46172	C	6.705588	11.663185	-2.927270
169	C	-0.096693	-18.068061	0.153693	C	-8.40000	1.35513	-6.72216	C	7.108081	12.697017	-3.830571
170	C	-1.184222	-15.169490	-1.631460	C	-6.85187	-1.66574	-5.66638	C	5.920366	10.491995	-1.290876
171	H	-1.483013	-14.537226	-2.449330	H	-6.26773	-2.34036	-5.06993	H	5.432458	10.152113	-0.392417
172	H	-1.392032	-12.609659	-0.693200	H	-6.46905	-3.89699	-6.89273	H	6.260744	7.935856	-0.963839
173	H	0.544122	-18.770581	2.012665	H	-9.27132	2.35488	-8.30538	H	8.389734	12.856182	-5.456750
174	H	0.593401	-17.518661	4.197645	H	-9.26990	-0.26320	-10.79754	H	9.825890	9.578670	-5.482238
175	H	0.204473	-15.770104	4.070050	H	-5.65491	-3.86937	-8.94679	H	8.455339	7.297842	-0.776113
176	H	-2.767022	-14.040851	1.570829	H	-7.43580	-5.39372	-9.71217	H	8.131704	5.488764	-2.376058
177	H	-2.260310	-12.302259	3.199686	H	-9.45432	-3.19435	-9.14628	H	7.563100	7.576017	-4.521910
178	H	0.003824	-11.128893	1.946755	H	-10.84784	-5.11749	-8.38874	H	5.839249	5.946560	-5.258059
179	H	1.324582	-13.031728	3.037752	H	-10.12765	-5.61044	-9.95092	H	7.310923	4.974654	-4.953525
180	H	0.287167	-12.093395	4.147421	H	-8.70063	-6.00226	-7.56442	H	5.541612	5.532763	-2.834315
181	H	-0.637404	-14.519272	2.892288	N	-9.00739	1.41314	-8.01973	N	7.987474	12.148573	-4.846545
182	N	0.167089	-17.924380	1.592548	N	-9.59318	0.50012	-10.15202	N	9.310251	10.427442	-5.856880
183	N	0.309816	-16.716045	3.655321	N	-8.71847	-0.88120	-8.55900	N	7.830614	9.879827	-4.199123
184	N	-0.494491	-15.678625	1.787669	N	-7.14527	-0.41384	-5.36508	N	5.949526	11.730961	-1.744514
185	N	-0.870767	-16.456302	-1.705184	N	-7.35328	-2.04652	-6.91499	N	6.628602	9.593167	-2.113855
186	N	-1.100965	-14.667707	-0.322255	O	-8.46057	-4.10006	-7.48584	O	6.133500	7.383223	-2.952718
187	O	-0.042851	-12.976232	0.912851	O	-9.21199	-6.47825	-8.28802	O	5.866857	4.820926	-3.461785
188	O	-0.440003	-13.993658	3.760673	O	-8.37090	2.38627	-6.01746	O	6.855891	13.912642	-3.854658
189	O	0.115964	-19.172443	-0.361366	H	-9.99543	1.36668	-10.48417	H	9.814586	11.120241	-6.399031

S4. G4

S4.1. Anti Conformation

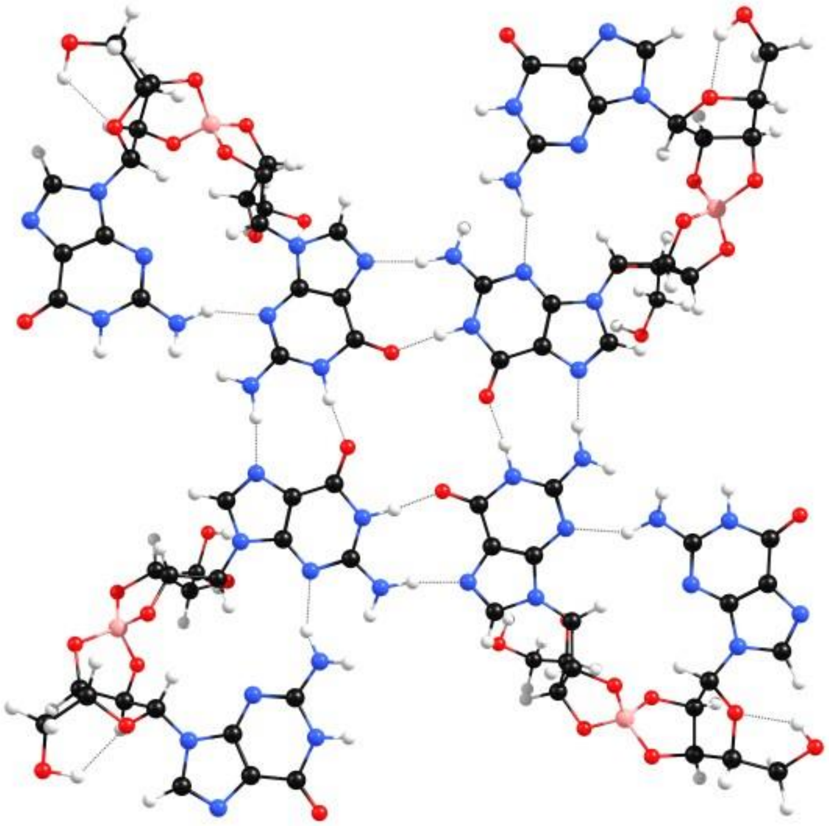

Figure S31. Optimized geometry of **Aβ-G4**, formed by four borate-guanosine diesters with two β-guanosines in anti conformation.

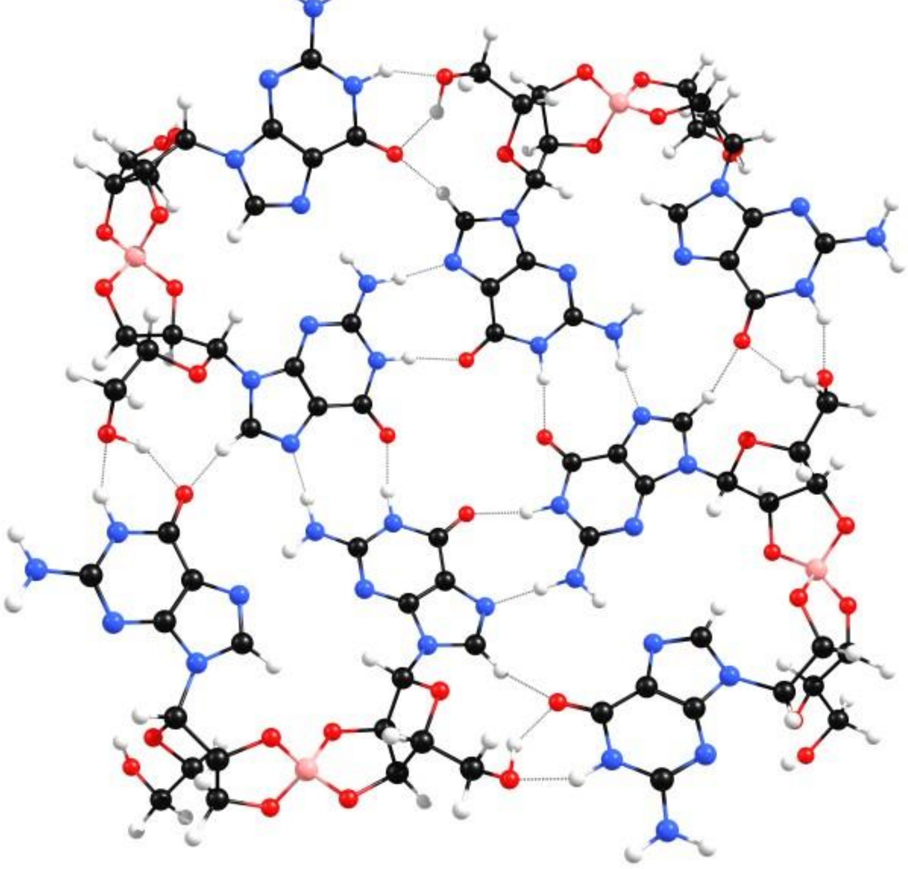

Figure S32. Optimized geometry of **Aγ-G4**, formed by four borate-guanosine diesters with one α-guanosine and one β-guanosine in anti conformation.

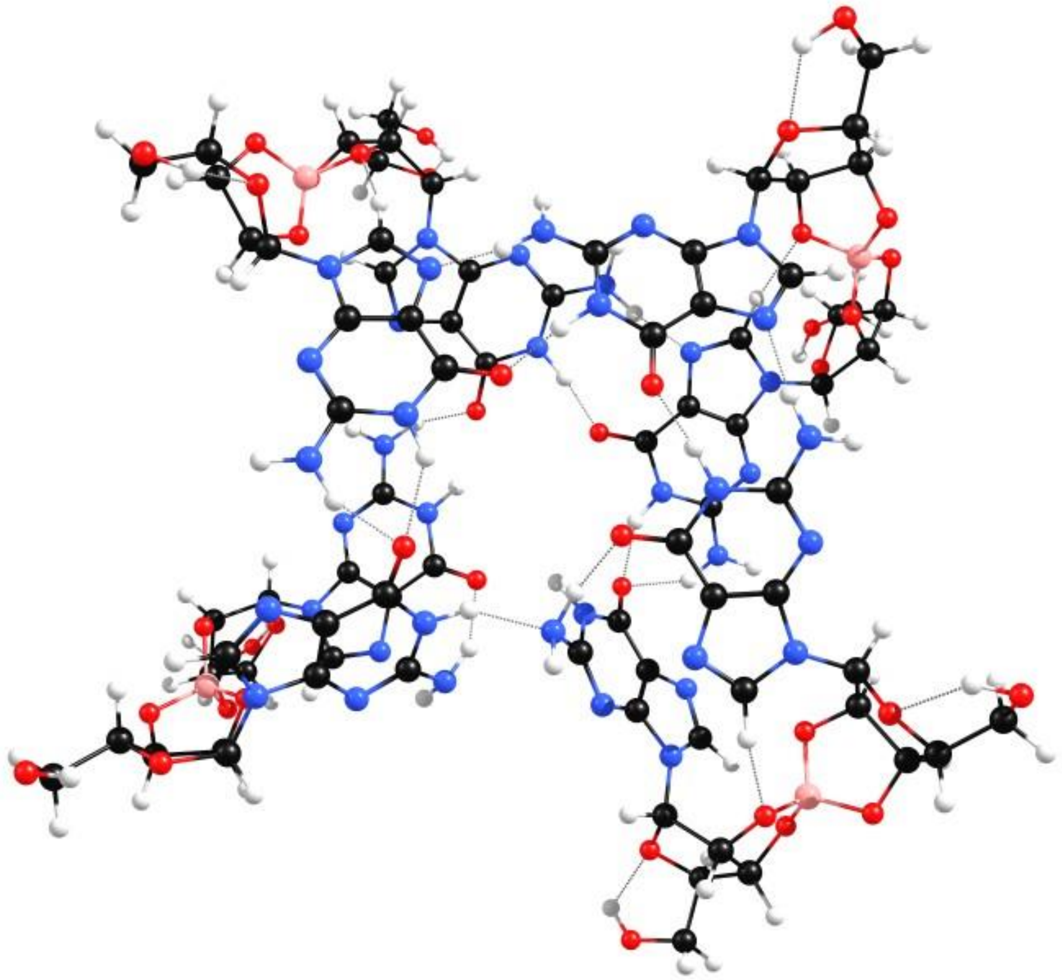

Figure S33. Optimized geometry of **Aα-G4**, formed by four borate-guanosine diesters with two α-guanosines in anti conformation.

Table S15. Optimized xyz cartesian coordinates for G4 structures in anti conformation.

	Aβ-G4				Aγ-G4				Aα-G4			
Number	Atom	x	y	z	Atom	x	y	z	Atom	x	y	z
1	C	-1.067833	4.650235	4.529461	C	2.610790	-10.093296	-2.772989	B	-0.328004	-3.724248	-1.213359
2	C	-2.299786	5.597278	4.649089	C	1.244806	-9.873902	-3.501232	O	0.584536	-3.615318	-2.403070
3	C	1.701079	1.836341	2.563106	C	6.576615	-8.764368	-2.023492	O	0.003668	-5.088685	-0.639736
4	C	-2.830978	5.365430	6.097586	C	0.345206	-11.085569	-3.034779	C	0.650050	-6.759067	-2.468561
5	C	-1.572931	4.897284	6.859356	C	1.063721	-11.612324	-1.782933	C	1.053409	-5.694782	-1.389633
6	C	-0.337725	2.689901	3.070149	C	4.360379	-8.752422	-1.655481	C	-2.102557	-9.968795	-3.295401
7	C	-1.897316	3.918128	7.980063	C	1.001145	-13.115429	-1.546826	C	1.681590	-4.535100	-2.223548
8	C	-1.022214	1.641148	2.459290	C	4.430734	-7.919194	-0.540289	C	2.157697	-5.178787	-3.525093
9	C	-0.300687	0.610772	1.791492	C	5.708081	-7.483972	-0.093538	C	-1.456398	-7.805923	-3.285136
10	C	-2.539658	2.895950	3.363706	C	2.309735	-8.328650	-0.847973	C	3.678572	-5.344714	-3.611856
11	H	-3.470330	3.302263	3.713811	H	1.230359	-8.321729	-0.796540	C	-2.518532	-7.371589	-4.073070
12	H	-0.191088	5.200630	4.183126	H	3.443903	-10.203993	-3.468033	C	-3.493266	-8.315047	-4.487286
13	H	1.681285	0.038457	1.444931	H	7.692359	-7.561983	-0.644646	C	-1.320956	-5.601261	-3.703674
14	H	3.698605	1.125908	2.298991	H	8.619823	-8.866114	-2.433100	H	-0.921338	-4.607200	-3.628387
15	H	3.412793	2.554977	3.305551	H	7.556721	-9.744490	-3.519244	H	0.741278	-7.783574	-2.103782
16	H	-3.038781	5.379731	3.868156	H	1.429574	-9.911788	-4.586116	H	-3.920047	-10.342811	-4.326971
17	H	-3.607735	4.600126	6.177185	H	0.303486	-11.875074	-3.799896	H	-1.271783	-11.460051	-2.237128
18	H	-0.991764	5.766069	7.177439	H	0.745784	-11.042116	-0.904774	H	1.795424	-6.166996	-0.734025
19	H	-0.978889	3.653552	8.525869	H	0.024275	-13.394731	-1.143148	H	2.520274	-4.069418	-1.681694
20	H	-2.605347	4.384642	8.670306	H	1.141924	-13.617151	-2.519741	H	1.749825	-4.658534	-4.394476
21	H	-1.972748	2.575713	6.593283	H	2.788355	-12.916900	-0.820900	H	4.155973	-4.400733	-3.894311
22	N	1.097644	0.789925	1.899301	N	6.758313	-7.943606	-0.942830	H	4.045103	-5.637864	-2.612533
23	N	3.040950	1.853164	2.671445	N	7.690834	-9.133976	-2.725401	H	3.273473	-7.013323	-4.487377
24	N	1.000332	2.854464	3.116729	N	5.391848	-9.211548	-2.415945	N	-3.201856	-9.621825	-4.062210
25	N	-2.398498	1.787717	2.651473	N	3.141538	-7.671762	-0.048774	N	-1.945887	-11.257924	-2.968186
26	N	-1.309459	3.508653	3.630712	N	3.020758	-9.028207	-1.856735	N	-1.197318	-9.060051	-2.869581

27	O	-0.817283	4.084201	5.863461	O	2.505002	-11.376242	-2.070127	N	-2.413641	-5.991446	-4.327731
28	O	-1.935219	6.985211	4.635918	O	0.525017	-8.712229	-3.119408	N	-0.684925	-6.675279	-3.025419
29	O	-3.284632	6.647279	6.570128	O	-0.940533	-10.546625	-2.758471	O	1.676048	-6.593682	-3.512582
30	O	-2.542496	2.741914	7.401481	O	2.022893	-13.516695	-0.585466	O	4.004892	-6.338915	-4.622124
31	O	-0.759459	-0.377957	1.175962	O	6.025464	-6.787365	0.902923	O	-4.533266	-8.090209	-5.173154
32	B	-2.402808	7.639560	5.950401	B	-0.875636	-9.056187	-2.644904	H	-2.559914	-12.050135	-3.277702
33	C	0.257988	8.923129	4.974932	C	-1.543086	-6.121108	-0.906692	C	-3.421509	-3.343445	0.214268
34	C	-0.653930	9.160808	6.212906	C	-2.081726	-7.559513	-1.174685	C	-2.470872	-2.691932	-0.846611
35	C	3.274893	6.084725	3.754269	C	1.227481	-5.407097	2.340475	C	-7.283059	-5.248676	-0.137379
36	C	-1.911764	9.895442	5.589576	C	-2.695520	-7.486757	-2.620840	C	-1.382411	-1.926975	0.001384
37	C	-1.536086	10.147880	4.105438	C	-2.439155	-6.029568	-3.084150	C	-1.830408	-2.061576	1.457176
38	C	2.574278	7.990503	4.719656	C	-0.797606	-5.345079	1.360271	C	-5.038750	-5.228071	-0.055588
39	C	-1.814552	11.572215	3.636081	C	-3.597860	-5.366715	-3.820436	C	-2.645267	-0.873042	1.986033
40	C	3.851124	8.530117	4.903971	C	-1.361941	-4.502979	2.312722	C	-4.904677	-6.604667	-0.180373
41	C	5.000357	7.795261	4.471803	C	-0.551299	-3.982190	3.360111	C	-6.065294	-7.417374	-0.304016
42	C	2.466991	10.023014	5.627634	C	-2.909086	-4.824708	0.812588	C	-2.868638	-5.818435	-0.112080
43	H	2.015068	10.914697	6.025248	H	-3.787346	-4.697030	0.212014	H	-1.795023	-5.703319	-0.163118
44	H	-0.020730	7.964878	4.534715	H	-0.460053	-6.136824	-1.065230	H	-4.379259	-2.815587	0.253721
45	H	5.350321	5.936673	3.616633	H	1.428863	-4.111455	4.018039	H	-8.130807	-7.092977	-0.555903
46	H	3.758352	4.515080	2.502077	H	3.207844	-5.526370	3.051708	H	-9.327075	-5.270143	-0.321060
47	H	2.172113	4.398437	3.219492	H	2.738549	-6.486483	1.606705	H	-8.579033	-3.681399	-0.097590
48	H	-0.137350	9.759157	6.971648	H	-2.821165	-7.826224	-0.412155	H	-3.060793	-2.027978	-1.487871
49	H	-2.123487	10.847238	6.086637	H	-3.773062	-7.684265	-2.623808	H	-1.295315	-0.869434	-0.290310
50	H	-2.005352	9.372088	3.495495	H	-1.538593	-6.039062	-3.711290	H	-0.977382	-2.283135	2.099116
51	H	-1.593600	11.661236	2.561143	H	-3.331368	-4.321856	-4.034046	H	-1.981639	-0.041035	2.245960
52	H	-2.864957	11.822644	3.809307	H	-3.779110	-5.897642	-4.759273	H	-3.335186	-0.525667	1.196432
53	H	-0.138430	11.956129	4.494692	H	-4.651154	-5.040854	-2.119646	H	-3.568954	-2.247651	2.968297
54	N	4.577271	6.542768	3.883061	N	0.770831	-4.512492	3.310960	N	-7.242948	-6.626527	-0.322760
55	N	3.098883	4.858071	3.192114	N	2.473091	-5.874558	2.392049	N	-8.503749	-4.689826	-0.096076
56	N	2.234211	6.796831	4.165697	N	0.442506	-5.832690	1.320631	N	-6.176325	-4.505779	0.008942

57	N	3.763048	9.804740	5.482620	N	-2.680431	-4.201357	1.956530	N	-3.547032	-6.953146	-0.199435
58	N	1.688311	8.943876	5.184349	N	-1.785349	-5.554046	0.402486	N	-3.743524	-4.725487	-0.010851
59	O	-0.052884	10.035386	4.049332	O	-2.242041	-5.241939	-1.860644	O	-2.727941	-3.240810	1.524560
60	O	-1.162271	7.939085	6.764000	O	-1.020420	-8.530013	-1.251692	O	-1.750455	-3.640856	-1.628190
61	O	-3.006485	8.966066	5.710633	O	-1.966996	-8.428864	-3.422996	O	-0.151857	-2.638109	-0.216771
62	O	-0.993654	12.478794	4.436045	O	-4.837477	-5.445875	-3.039527	O	-3.356424	-1.288084	3.185350
63	O	6.208820	8.059183	4.524719	O	-0.880574	-3.138329	4.227448	O	-6.142063	-8.664268	-0.397704
64	C	8.447111	-1.250069	1.464837	C	-9.263107	0.501960	-1.119169	B	-1.765538	-17.390620	-1.568026
65	C	9.309069	0.040890	1.387713	C	-9.277966	2.014363	-0.723672	O	-1.697112	-17.622527	-0.097697
66	C	5.044135	-3.483018	-0.308376	C	-7.553553	-2.716843	-3.290223	O	-0.991625	-16.085364	-1.755649
67	C	9.762651	0.315397	2.861588	C	-10.480136	2.148794	0.291467	C	-1.016190	-14.547061	0.287062
68	C	9.617582	-1.051618	3.572189	C	-10.746217	0.701490	0.734090	C	-0.360024	-15.715552	-0.531562
69	C	6.031026	-1.727659	0.733792	C	-7.638142	-1.309430	-1.542288	C	-3.037619	-10.738027	-0.056552
70	C	8.979057	-0.908906	4.950205	C	-12.194936	0.335751	1.028251	C	-0.512325	-16.961706	0.400963
71	C	4.818474	-1.089731	0.992796	C	-6.623122	-1.944979	-0.828303	C	-0.686998	-16.405586	1.812457
72	C	3.600309	-1.656106	0.522701	C	-6.024281	-3.108852	-1.385298	C	-2.953587	-12.995539	-0.002608
73	C	6.339858	0.106563	1.970250	C	-7.233500	-0.238577	0.387315	C	0.539439	-16.598551	2.709884
74	H	6.855354	0.796530	2.613465	H	-7.296775	0.530448	1.141332	C	-4.331952	-13.154773	-0.032649
75	H	8.735772	-1.951490	0.680997	H	-9.400433	0.341605	-2.188782	C	-5.167208	-12.004440	-0.069885
76	H	2.934789	-3.384297	-0.447796	H	-6.055251	-4.237026	-3.109333	C	-3.516780	-15.172952	0.043742
77	H	4.298711	-5.260354	-1.227939	H	-7.569881	-3.986042	-4.944824	H	-3.371199	-16.236798	0.055183
78	H	6.016052	-5.160049	-0.800851	H	-8.702094	-2.645011	-4.975453	H	-0.520339	-13.587524	0.132472
79	H	8.721544	0.857401	0.951026	H	-9.461135	2.603004	-1.636250	H	-4.996706	-9.934991	-0.181819
80	H	9.159217	1.073991	3.367458	H	-11.375574	2.555776	-0.201533	H	-1.465187	-9.521050	-0.302247
81	H	10.584670	-1.555534	3.572993	H	-10.080158	0.436932	1.561173	H	0.694388	-15.452250	-0.679834
82	H	8.972314	-1.876113	5.474032	H	-12.496042	0.730668	2.002174	H	0.372302	-17.613491	0.333492
83	H	9.534676	-0.180191	5.545569	H	-12.824409	0.794158	0.245912	H	-1.599691	-16.791210	2.272478
84	H	7.246239	-1.019142	4.095064	H	-11.730821	-1.413687	0.329760	H	0.580144	-17.622077	3.095487
85	N	3.811368	-2.890779	-0.133450	N	-6.548297	-3.414751	-2.676276	H	1.439342	-16.418028	2.096330
86	N	5.107986	-4.706465	-0.861432	N	-7.956138	-3.153867	-4.522076	H	0.093159	-14.860414	3.418485

87	N	6.201439	-2.886925	0.065587	N	-8.144983	-1.657580	-2.756658	N	-4.418437	-10.804676	-0.070557
88	N	5.030809	0.053010	1.768443	N	-6.390250	-1.263366	0.374749	N	-2.459261	-9.532428	-0.097674
89	N	7.008286	-0.947266	1.333793	N	-8.041158	-0.229623	-0.778981	N	-2.255476	-11.845416	-0.010853
90	O	8.634330	-1.849211	2.787105	O	-10.434216	-0.105135	-0.475967	N	-4.662832	-14.517788	-0.007720
91	O	10.537079	-0.149861	0.669717	O	-8.141093	2.463730	-0.005124	N	-2.420810	-14.287493	0.040207
92	O	11.143586	0.711534	2.788861	O	-10.024708	2.982878	1.348411	O	-0.770323	-14.919748	1.687495
93	O	7.621879	-0.386327	4.773311	O	-12.349090	-1.115327	1.057857	O	0.466505	-15.689628	3.842626
94	O	2.439574	-1.203265	0.648609	O	-5.142213	-3.872461	-0.919055	O	-6.419014	-11.956417	-0.096827
95	B	11.704577	0.003352	1.627044	B	-8.544005	3.174744	1.279415	H	-2.960245	-8.609109	-0.168156
96	C	12.633806	-1.951922	-0.406712	C	-5.362717	3.146722	2.565121	C	-3.712681	-17.113636	-4.528691
97	C	13.224883	-1.659759	1.018353	C	-6.835348	3.486695	2.950368	C	-3.381880	-17.965770	-3.251929
98	C	9.439949	-4.717824	-1.450524	C	-3.793301	-0.855147	3.134949	C	-6.611887	-14.355683	-6.124353
99	C	13.930108	-0.286387	0.806244	C	-7.123044	4.855781	2.231096	C	-1.990060	-18.638205	-3.562104
100	C	14.409882	-0.314956	-0.633646	C	-5.810718	5.185264	1.474373	C	-1.438249	-17.804431	-4.728828
101	C	11.591771	-4.145623	-1.127806	C	-4.049160	1.314996	3.675124	C	-4.790935	-14.899427	-4.929871
102	C	15.803294	-0.991105	-0.758404	C	-5.370997	6.643278	1.537735	C	-0.593166	-18.550275	-5.753091
103	C	12.121168	-5.326488	-1.661130	C	-3.046344	1.312816	4.638976	C	-4.480211	-13.585534	-4.599715
104	C	11.243720	-6.353578	-2.135138	C	-2.270144	0.138014	4.845912	C	-5.299152	-12.527834	-5.080239
105	C	13.824394	-4.073510	-1.209271	C	-3.767118	3.366218	4.537375	C	-2.982477	-14.812833	-3.596183
106	H	14.800686	-3.616588	-1.215213	H	-3.819625	4.432984	4.626521	H	-2.178750	-15.155718	-2.960961
107	H	11.597017	-1.612191	-0.435001	H	-5.390024	2.409509	1.756742	H	-4.647996	-17.408274	-5.006394
108	H	9.187278	-6.628287	-2.235432	H	-2.152971	-1.810713	4.100363	H	-7.105772	-12.345802	-6.149142
109	H	7.532585	-4.830886	-2.214122	H	-3.631702	-2.825208	2.411162	H	-8.338849	-13.854215	-7.121594
110	H	7.687906	-3.739761	-0.871549	H	-4.924469	-1.761559	1.759728	H	-7.964017	-15.588102	-7.005714
111	H	13.884093	-2.471256	1.340947	H	-6.924879	3.549646	4.039925	H	-4.177702	-18.707408	-3.112229
112	H	14.765650	-0.131968	1.506593	H	-7.346419	5.659947	2.940166	H	-2.097806	-19.689178	-3.858948
113	H	14.374051	0.672457	-1.094774	H	-5.944068	4.866103	0.433082	H	-0.924302	-16.920348	-4.337767
114	H	16.589466	-0.227118	-0.784337	H	-4.399862	6.743494	1.032020	H	0.407303	-18.740797	-5.354590
115	H	15.971300	-1.622118	0.127234	H	-6.112740	7.266966	1.030163	H	-1.085620	-19.516575	-5.957423
116	H	14.893274	-1.839910	-2.240005	H	-4.697433	6.469135	3.443469	H	-1.357458	-17.328248	-7.051831

117	N	9.874506	-5.920376	-1.985696	N	-2.730521	-0.941604	4.037639	N	-6.379564	-13.021529	-5.837738
118	N	8.085599	-4.521416	-1.419923	N	-4.172506	-1.928580	2.444035	N	-7.696681	-14.621095	-6.879543
119	N	10.279356	-3.802257	-0.992288	N	-4.472542	0.298350	2.923024	N	-5.813153	-15.343077	-5.693755
120	N	13.517936	-5.263686	-1.701543	N	-2.895721	2.597725	5.169393	N	-3.348795	-13.553056	-3.777846
121	N	12.680117	-3.347129	-0.827279	N	-4.511694	2.625577	3.610912	N	-3.834219	-15.687520	-4.295210
122	O	13.451419	-1.177072	-1.377181	O	-4.750912	4.421252	2.144659	O	-2.646382	-17.421095	-5.498606
123	O	12.211262	-1.380736	1.989443	O	-7.775208	2.547961	2.394147	O	-3.162641	-17.216815	-2.052140
124	O	12.869625	0.669159	0.997307	O	-8.208579	4.617290	1.322147	O	-1.206534	-18.510691	-2.367837
125	O	15.864159	-1.807767	-1.967406	O	-5.295522	7.114718	2.924887	O	-0.453757	-17.748952	-6.962409
126	O	11.478627	-7.468615	-2.619648	O	-1.276574	0.006429	5.599506	O	-5.173220	-11.290824	-4.910992
127	C	-7.756564	-3.712534	0.019573	C	2.234880	10.409402	6.563942	O	-14.657265	-17.388501	-4.726628
128	C	-8.649717	-4.665824	-0.826687	C	0.676737	10.381906	6.435492	B	-14.210666	-16.100033	-5.307539
129	C	-4.348080	-1.098823	0.968938	H	0.299378	11.108164	5.715357	O	-12.731573	-16.112502	-5.186971
130	C	-9.117468	-5.762581	0.179332	N	0.097013	9.102457	6.022731	O	-14.805418	-14.905847	-4.586030
131	C	-9.065221	-5.032343	1.538295	O	0.147567	10.807730	7.735965	O	-14.667607	-15.902269	-6.737347
132	C	-5.337216	-2.921116	0.052336	C	2.506155	10.749859	8.082571	C	-13.553390	-18.101744	-4.141082
133	C	-8.705286	-5.962662	2.689366	H	2.615142	11.206782	5.906200	C	-12.292020	-17.173539	-4.338471
134	C	-4.124115	-3.510683	-0.296389	O	2.883954	9.168409	6.344151	C	-11.883217	-16.740686	-2.881602
135	C	-2.900110	-2.829594	-0.039185	N	-3.318924	8.556140	4.020052	H	-11.003919	-17.305462	-2.554450
136	C	-5.658891	-4.943323	-0.832005	C	-2.820677	9.828523	4.100581	N	-11.564806	-15.358127	-2.695169
137	H	-6.183616	-5.818896	-1.166138	N	-3.521918	10.817789	3.468094	O	-13.045729	-17.075086	-2.009296
138	H	-8.067605	-2.674663	-0.109976	N	-1.709983	10.143024	4.752212	H	-11.443428	-17.698730	-4.791711
139	H	-2.233294	-1.072667	0.876967	C	1.161175	10.466348	8.769816	N	-8.376881	-13.479173	-1.160740
140	H	-3.602190	0.623621	1.986949	H	2.772063	11.809925	8.211136	C	-8.344414	-14.860312	-1.186456
141	H	-5.339507	0.280159	2.024381	O	3.547006	9.882467	8.511890	N	-7.262702	-15.488845	-0.685423
142	H	-8.100735	-5.064202	-1.688782	C	0.788898	11.381583	9.929213	N	-9.347599	-15.608048	-1.688322
143	H	-8.472988	-6.644874	0.217128	H	1.098428	9.408487	9.042588	C	-13.734923	-18.231974	-2.627487
144	H	-9.989855	-4.470982	1.689540	C	-1.506262	7.739282	5.296172	H	-13.422923	-19.088953	-4.609773
145	H	-8.737098	-5.411832	3.641497	C	-1.091718	9.070100	5.316956	C	-13.098231	-19.488432	-2.013066
146	H	-9.423658	-6.786043	2.723428	H	1.346672	11.103074	10.827151	H	-14.789183	-18.144435	-2.363930

147	H	-6.874217	-5.725870	2.120624	H	1.061948	12.412422	9.644706	C	-10.527430	-13.489767	-2.107141
148	N	-3.111117	-1.598723	0.625000	O	-0.636163	11.267249	10.222875	C	-10.391482	-14.875527	-2.122905
149	N	-4.417321	0.050618	1.664178	C	-2.723501	7.414500	4.635225	H	-13.737442	-20.362181	-2.181934
150	N	-5.505641	-1.717328	0.638516	N	-0.578200	6.942118	5.981158	O	-12.956199	-19.283978	-0.580484
151	N	-4.344784	-4.773827	-0.851178	O	-3.322863	6.315970	4.526758	C	-9.444320	-12.689795	-1.655545
152	N	-6.324428	-3.827734	-0.309499	H	1.279581	7.483651	6.920364	N	-11.778644	-13.110677	-2.616226
153	O	-7.896166	-4.112231	1.426240	C	0.376368	7.763521	6.400194	O	-9.343828	-11.432694	-1.667258
154	O	-9.873261	-4.041174	-1.241293	H	-4.185647	8.298911	3.482398	H	-13.346228	-14.337847	-3.430792
155	O	-10.471255	-6.091879	-0.186448	H	-4.399836	10.636239	3.002703	C	-12.378466	-14.232726	-2.965885
156	O	-7.383227	-6.530607	2.435396	H	-3.157931	11.758236	3.531954	H	-7.575106	-12.938612	-0.750222
157	O	-1.736879	-3.202378	-0.310729	H	-1.035344	11.161771	9.311396	H	-7.277435	-16.498026	-0.781168
158	B	-11.063834	-4.850233	-0.692340	B	3.807326	8.813146	7.498959	H	-12.766276	-18.295259	-0.543216
159	C	-11.732971	-1.824614	-0.809568	O	3.455204	7.431888	7.939033	H	-6.364575	-15.042122	-0.395422
160	C	-12.533761	-3.075083	-0.344966	O	5.247440	8.767933	7.153024	C	-15.446422	-14.055074	-5.523180
161	C	-8.658138	0.744258	0.814657	C	4.549964	6.527316	7.703182	C	-14.593359	-12.899427	-6.156584
162	C	-12.886699	-3.797812	-1.708317	C	4.263686	5.550536	6.522457	H	-14.688935	-11.968676	-5.595544
163	C	-12.418155	-2.818429	-2.817526	H	3.399130	5.933812	5.971070	N	-13.158529	-13.115282	-6.314040
164	C	-10.791766	0.143087	0.437009	N	3.994535	4.169195	6.859332	O	-15.213369	-12.711251	-7.474972
165	C	-13.464089	-2.567603	-3.899124	O	5.492171	5.532472	5.706494	C	-15.766309	-14.962376	-6.739970
166	C	-11.357571	1.320021	0.937744	C	5.739748	7.419457	7.193958	H	-16.345732	-13.590419	-5.101123
167	C	-10.509257	2.356214	1.441743	H	4.800756	5.954363	8.602073	N	-9.993684	-10.929480	-5.211144
168	C	-13.011396	0.119611	0.237546	N	0.699277	1.927496	6.410882	C	-11.176418	-10.760315	-4.503471
169	H	-13.983782	-0.250779	-0.035796	C	0.543955	3.315981	6.381350	N	-11.067252	-10.045800	-3.318595
170	H	-10.681964	-2.106926	-0.875569	N	-0.662671	3.848955	6.201124	N	-12.320039	-11.313326	-4.880454
171	H	-8.469816	2.609731	1.727710	N	1.588786	4.166516	6.530693	C	-15.789325	-14.016694	-7.939110
172	H	-6.684620	1.344042	0.715036	C	6.055118	6.887121	5.772525	H	-16.737376	-15.468652	-6.619989
173	H	-6.915498	-0.381346	0.604499	H	6.630122	7.320972	7.824549	C	-17.206833	-13.629439	-8.372482
174	H	-13.419307	-2.781720	0.229829	C	7.541131	6.740551	5.455560	H	-15.196673	-14.393080	-8.775496
175	H	-13.958753	-3.993367	-1.809850	H	5.570280	7.562270	5.056371	C	-10.998482	-12.623179	-6.480188
176	H	-11.452911	-3.164498	-3.194837	C	2.994940	2.186498	6.859149	C	-12.182146	-12.247673	-5.845172

177	H	-13.039148	-1.924059	-4.685230	C	2.752402	3.551719	6.745837	H	-17.659081	-14.426323	-8.971668
178	H	-13.778955	-3.516079	-4.343391	H	7.654161	6.302729	4.453609	H	-17.814065	-13.488405	-7.461607
179	H	-14.183375	-1.330043	-2.618181	H	8.010402	7.728586	5.476751	O	-17.160731	-12.424392	-9.184437
180	N	-9.126473	1.947191	1.320675	O	8.216049	5.923411	6.469879	C	-9.815229	-11.893693	-6.218072
181	N	-7.309903	0.551801	0.816384	C	1.937724	1.260562	6.636424	N	-11.214359	-13.725444	-7.319994
182	N	-9.476523	-0.191800	0.353810	N	4.361388	1.961771	7.050430	O	-8.690428	-12.046632	-6.785028
183	N	-12.753498	1.283424	0.809925	O	2.018975	0.009631	6.596111	H	-13.027821	-14.850642	-7.591923
184	N	-11.849647	-0.626512	-0.008382	H	5.997568	3.316597	7.028106	C	-12.496828	-14.005951	-7.193736
185	O	-12.274925	-1.495805	-2.147443	C	4.939377	3.151058	7.035850	H	-9.251111	-10.227882	-5.092490
186	O	-11.720762	-4.007695	0.379973	H	-0.099853	1.295268	6.176860	H	-10.345408	-10.482292	-2.664889
187	O	-12.116249	-5.016037	-1.715378	H	-1.513517	3.297062	5.938278	H	-11.982069	-10.033268	-2.857636
188	O	-14.629159	-1.954428	-3.265900	H	-0.681432	4.877473	6.143137	H	-16.456875	-11.883894	-8.716165
189	O	-10.770612	3.464924	1.926721	H	7.727562	5.025300	6.507211	H	-12.125659	-19.676652	-2.501806
190	C	1.787885	-9.504210	-3.422052	C	12.459437	-0.387626	5.376357	B	-11.746878	-1.744785	-6.446105
191	C	2.877333	-9.908173	-4.454609	C	12.718477	-1.519510	4.330310	O	-11.950722	-2.010295	-7.933896
192	C	-0.998215	-6.384008	-1.971512	C	13.213143	-2.745556	5.186964	O	-10.289096	-1.507824	-6.316885
193	C	3.808128	-10.911296	-3.689968	H	13.495540	-1.173546	3.631146	C	-8.948619	-2.721205	-8.102164
194	C	2.943743	-11.424882	-2.513738	O	11.560968	-1.993546	3.656741	C	-9.699808	-1.437828	-7.611597
195	C	1.037961	-7.237518	-2.487382	H	13.122683	0.467302	5.236980	C	-6.498363	-5.729671	-6.300376
196	C	3.729024	-11.467293	-1.206077	N	11.110029	0.175616	5.366645	C	-10.887639	-1.308183	-8.613483
197	C	1.726657	-6.166818	-1.919258	O	12.778417	-0.946520	6.693578	C	-10.418926	-1.991101	-9.892605
198	C	1.006392	-5.067198	-1.372298	C	10.888192	1.519216	5.602556	C	-8.465511	-4.902997	-7.001317
199	C	3.241696	-7.594604	-2.526469	C	9.848114	-0.462739	5.444880	C	-9.958770	-1.003978	-10.972799
200	H	4.158484	-8.146248	-2.628752	C	12.693786	-2.429346	6.598504	C	-9.198199	-6.061631	-6.730368
201	H	0.794836	-9.556868	-3.870202	H	14.309835	-2.822999	5.195635	C	-8.532122	-7.172598	-6.127099
202	H	-0.972869	-4.502301	-1.005807	O	12.604769	-3.898440	4.618056	C	-10.617627	-4.622490	-7.552928
203	H	-2.988629	-5.806483	-1.465375	B	11.403112	-3.494235	3.820477	H	-11.490487	-4.074147	-7.857695
204	H	-2.702043	-7.414938	-2.153670	N	10.007072	3.959263	6.042742	H	-7.872531	-2.671880	-7.928901
205	H	3.400105	-9.014542	-4.816237	C	11.335095	3.711620	5.815102	H	-6.565178	-7.667094	-5.562986
206	H	4.725956	-10.450503	-3.314602	N	11.832058	2.500568	5.603564	H	-4.705208	-4.789361	-6.174694

207	H	2.486897	-12.373434	-2.796829	C	13.568319	-2.897235	7.755205	H	-9.001235	-0.596488	-7.694320
208	H	3.123286	-11.925140	-0.410247	H	11.658346	-2.769915	6.693847	H	-11.142324	-0.251013	-8.793385
209	H	4.646948	-12.045593	-1.338454	H	13.441532	-3.971005	7.915718	H	-11.161738	-2.696379	-10.271230
210	H	3.247503	-9.612644	-0.932924	H	14.619653	-2.699032	7.482415	H	-10.817881	-0.587977	-11.509019
211	N	-0.392087	-5.266998	-1.437899	O	13.195359	-2.203797	8.983242	H	-9.426050	-0.177200	-10.471276
212	N	-2.334131	-6.504751	-1.886867	C	9.519582	1.657792	5.829237	H	-8.596988	-2.315783	-11.338948
213	N	-0.301023	-7.376297	-2.573506	C	9.001303	2.949871	6.109847	N	-7.144968	-6.923432	-5.972542
214	N	3.103079	-6.408876	-1.949414	N	8.890927	0.409070	5.734923	N	-5.185723	-5.671722	-6.061842
215	N	2.006500	-8.138383	-2.903988	H	12.972641	-1.285482	8.653919	N	-7.151569	-4.683657	-6.823787
216	O	1.880923	-10.408365	-2.275787	O	7.832758	3.307718	6.400243	N	-10.546828	-5.859928	-7.105412
217	O	2.350866	-10.673863	-5.549104	H	9.720048	-1.516459	5.246500	N	-9.364355	-3.981099	-7.514927
218	O	4.095166	-11.975511	-4.614950	O	10.089983	-3.732047	4.503650	O	-9.161742	-2.730550	-9.558604
219	O	4.119904	-10.097984	-0.861610	O	11.345083	-4.266083	2.562425	O	-9.111439	-1.691282	-11.934477
220	O	1.464037	-4.023160	-0.854832	C	9.232060	-4.555980	3.690371	O	-9.025994	-8.266343	-5.726520
221	B	2.906612	-12.083626	-5.477235	C	10.075968	-4.923816	2.416140	H	-4.701570	-6.499231	-5.660074
222	C	0.190232	-12.414349	-6.654820	C	8.011839	-3.762910	3.132785	C	-13.932590	-2.988390	-3.754949
223	C	1.114979	-13.493941	-5.987530	H	8.880768	-5.439286	4.234528	C	-13.582053	-2.615924	-5.233943
224	C	-2.686170	-9.856081	-4.622713	H	8.210469	-2.696325	3.277764	C	-11.789350	-6.773851	-4.248226
225	C	2.206074	-13.712759	-7.079677	N	6.713371	-4.046053	3.707772	C	-13.715386	-1.040498	-5.294881
226	C	1.485872	-13.495537	-8.397949	O	7.905198	-4.130462	1.708769	C	-13.700870	-0.606345	-3.823612
227	C	-2.065311	-11.706922	-5.739718	C	5.991198	-3.151414	4.493050	C	-12.273736	-4.836228	-3.230821
228	C	0.797741	-14.793358	-8.897573	C	5.818181	-5.048121	3.309357	C	-14.601925	0.565447	-3.456793
229	C	-3.365902	-12.193914	-5.915423	C	9.275353	-4.343766	1.223260	C	-11.423768	-5.073906	-2.141316
230	C	-4.479985	-11.468108	-5.383998	H	10.180205	-6.007335	2.292146	C	-10.755368	-6.325206	-2.044868
231	C	-2.077660	-13.563915	-6.982630	N	4.183275	-1.681047	5.730473	C	-12.288020	-3.124704	-1.793935
232	H	-1.698316	-14.292404	-7.680544	N	5.922378	-0.170655	6.267087	H	-12.572255	-2.168372	-1.398656
233	H	0.488817	-11.426228	-6.300237	C	5.524495	-1.294965	5.677451	H	-14.796513	-3.658286	-3.715179
234	H	-4.734037	-9.728740	-4.278248	N	6.465735	-2.029703	5.034133	H	-10.674588	-8.128237	-3.151007
235	H	-3.110980	-7.896092	-4.161397	H	5.273026	0.543677	6.674969	H	-10.892593	-8.146686	-5.428879
236	H	-1.518706	-8.400363	-3.687917	H	6.915241	0.065532	6.120471	H	-11.957796	-7.088917	-6.192158

237	H	0.540884	-14.387336	-5.724802	C	9.146730	-5.269382	0.017811	H	-14.311200	-3.099620	-5.897668
238	H	2.655806	-14.716430	-7.027254	H	9.755426	-3.400412	0.934142	H	-14.665468	-0.745071	-5.766321
239	H	2.133918	-13.044121	-9.149715	H	8.493777	-4.794194	-0.727482	H	-12.667239	-0.457709	-3.501783
240	H	1.445074	-15.307909	-9.617816	H	10.136309	-5.438517	-0.416283	H	-14.146917	1.506343	-3.777240
241	H	0.640679	-15.465911	-8.040885	O	8.625451	-6.582917	0.413670	H	-15.560872	0.434701	-3.985722
242	H	-0.619328	-13.518945	-9.237510	C	4.693140	-3.649452	4.543427	H	-14.897299	-0.336099	-1.764978
243	N	-4.004730	-10.264988	-4.743271	C	3.665874	-2.878127	5.156124	N	-11.016850	-7.146776	-3.150210
244	N	-2.477001	-8.668195	-3.972374	N	4.611974	-4.836726	3.808173	N	-11.761035	-7.598232	-5.321101
245	N	-1.677465	-10.565169	-5.102666	H	7.725357	-6.427571	0.874046	N	-12.509293	-5.646632	-4.276085
246	N	-3.352945	-13.356487	-6.692214	H	6.057989	-5.809260	2.594831	N	-11.434463	-3.982830	-1.260119
247	N	-1.238692	-12.587042	-6.412438	H	9.600559	4.919414	6.178400	N	-12.833611	-3.595647	-3.001130
248	O	0.398191	-12.516613	-8.121463	N	12.180129	4.786910	5.809365	O	-14.316681	-1.749006	-3.082964
249	O	1.843386	-12.981600	-4.868086	H	11.857057	5.720220	6.021038	O	-12.238900	-2.878556	-5.623868
250	O	3.160568	-12.667148	-6.814817	H	13.160316	4.606610	5.642962	O	-12.571726	-0.565963	-6.004238
251	O	-0.484056	-14.476955	-9.522951	O	2.439606	-3.137807	5.194613	O	-14.791868	0.627597	-2.010294
252	O	-5.694407	-11.708473	-5.401148	H	3.461090	-1.040935	6.132708	O	-10.010697	-6.718792	-1.097334

S4.2. Syn Conformation

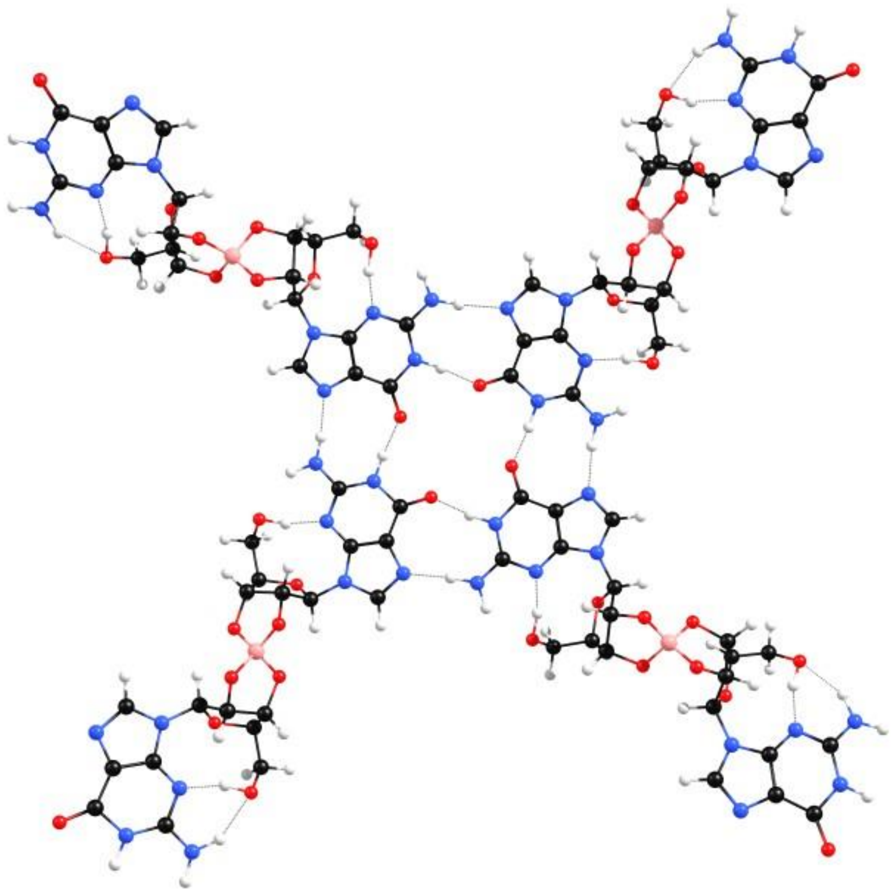

Figure S34. Optimized geometry of **Sβ-G4**, formed by four borate-guanosine diesters with two β-guanosines in syn conformation.

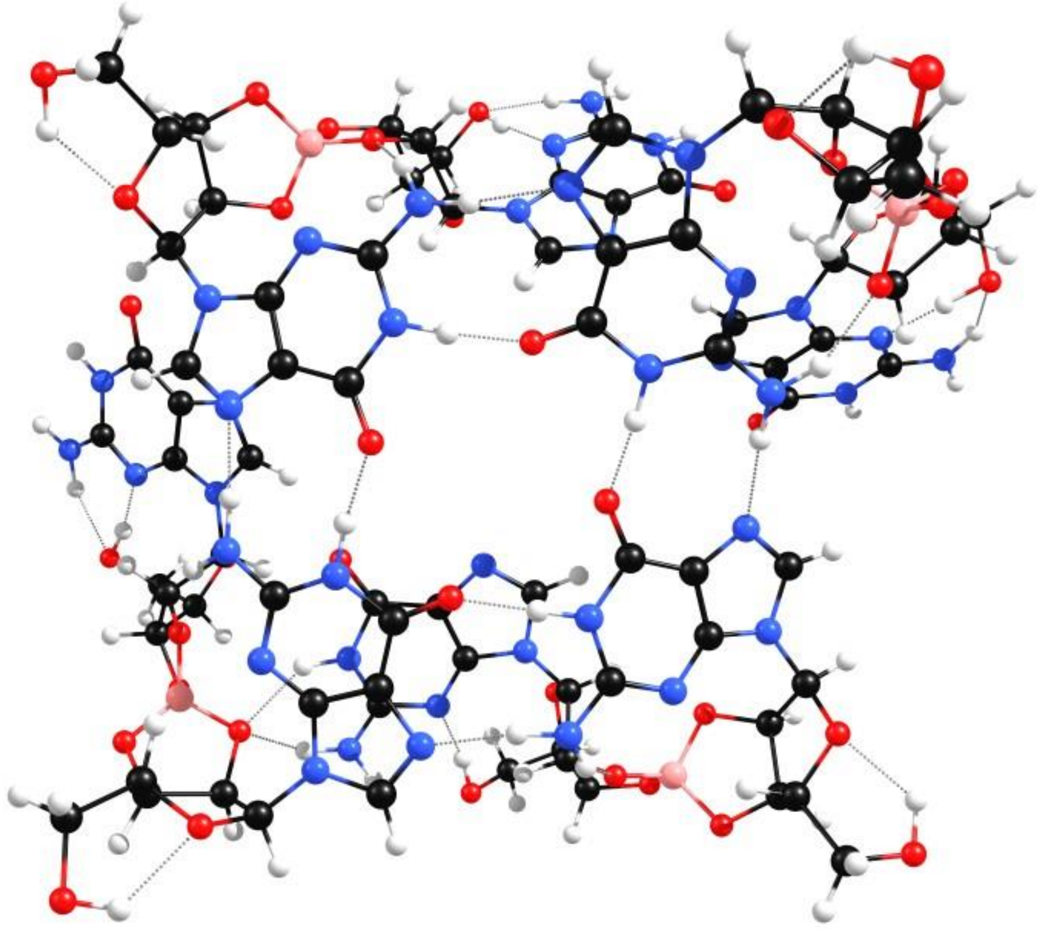

Figure S35. Optimized geometry of **Sγ-G4**, formed by four borate-guanosine diesters with one α-guanosine and one β-guanosine in syn conformation.

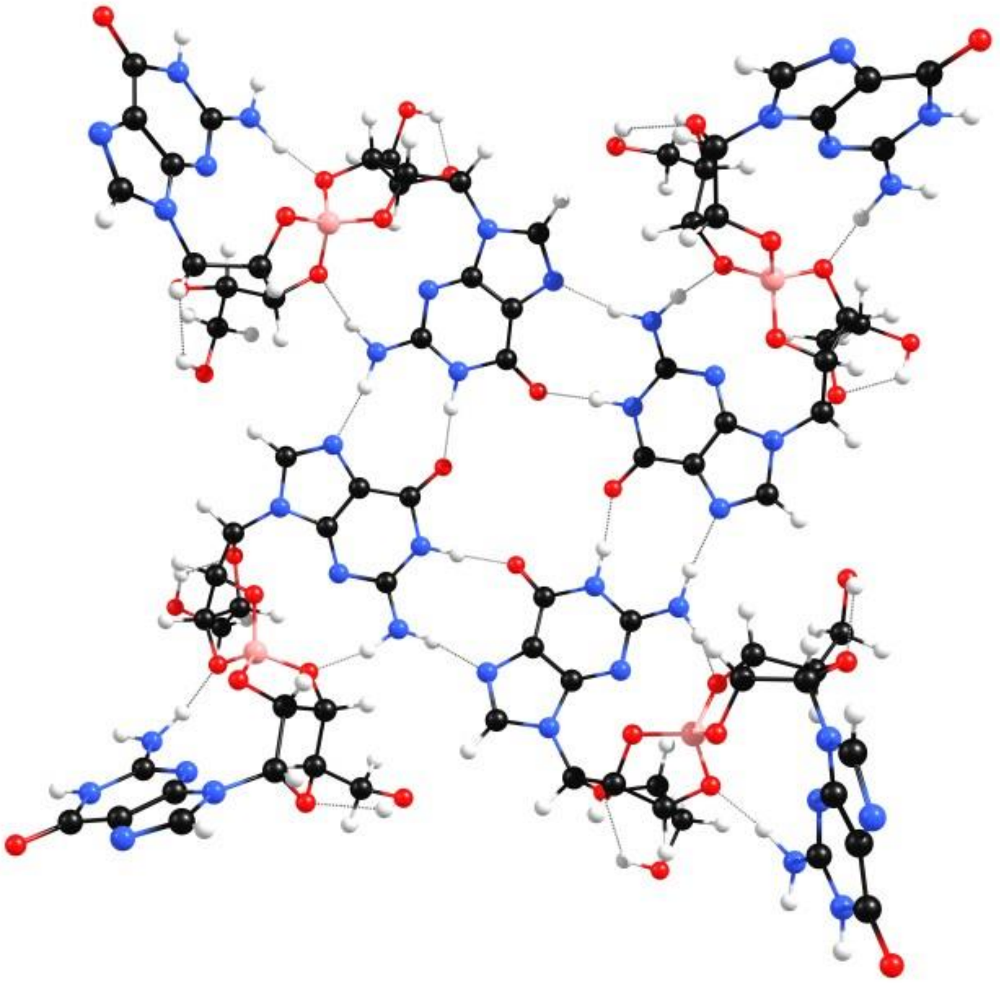

Figure S36. Optimized geometry of **Sα-G4**, formed by four borate-guanosine diesters with two α-guanosines in syn conformation.

Table S16. Optimized xyz cartesian coordinates for G4 structures in syn conformation.

	Sβ-G4				Sγ-G4				Sα-G4			
**Number**	**Atom**	**x**	**y**	**z**	**Atom**	**x**	**y**	**z**	**Atom**	**x**	**y**	**z**
1	O	-12.725251	7.727121	-4.214539	O	-3.792166	1.087607	1.064800	C	4.205039	-4.416893	2.048952
2	O	-15.037114	7.324967	-3.886278	O	-3.245615	2.809310	-0.478398	C	4.968007	-3.318943	1.214382
3	B	-13.709390	7.048913	-3.276603	B	-3.240982	1.286433	-0.300226	C	1.075993	-3.912227	-0.931307
4	C	-11.094634	5.966556	-2.313764	C	-2.867753	-0.142638	-3.427554	C	5.795953	-4.148189	0.187628
5	C	-12.554194	5.542294	-1.947884	C	-3.259407	-0.461811	-1.954350	C	4.992350	-5.447947	0.036886
6	C	-9.559464	2.958119	0.499779	C	-2.807703	-4.423704	-4.112399	C	1.834340	-4.145695	1.170046
7	C	-13.000566	6.685236	-0.992641	C	-1.906140	-0.495120	-1.175854	C	5.826098	-6.675808	-0.312871
8	C	-11.708554	7.210473	-0.346358	C	-0.800570	-0.567861	-2.250265	C	0.587489	-3.980179	1.788655
9	C	-9.759454	3.945266	-1.545489	C	-3.546334	-2.312293	-4.516647	C	-0.580324	-3.810014	0.979538
10	C	-11.471552	6.677718	1.073322	C	-0.015128	-1.889559	-2.223812	C	2.034360	-4.132247	3.386413
11	C	-8.874342	3.067915	-2.164751	C	-4.480710	-2.685339	-5.485274	H	2.532566	-4.172267	4.340834
12	C	-8.264429	2.032241	-1.396852	C	-4.690968	-4.067326	-5.766232	H	4.603120	-4.450088	3.067097
13	C	-9.538099	4.454721	-3.697629	C	-4.584695	-0.520778	-5.333622	H	-0.984069	-3.537555	-1.038237
14	H	-9.704593	4.980970	-4.620797	H	-4.817699	0.528838	-5.426714	H	0.628766	-3.345434	-2.860732
15	H	-11.125784	6.546921	-3.241203	H	-3.099050	0.899206	-3.656361	H	2.348464	-3.348954	-2.346726
16	H	-8.289412	1.270995	0.555980	H	-3.795718	-5.879624	-5.240174	H	5.615101	-2.756489	1.902056
17	H	-9.511924	2.268910	2.510659	H	-1.950310	-6.253278	-3.574459	H	6.815230	-4.367171	0.520949
18	H	-10.554775	3.660459	2.073038	H	-1.260109	-4.767714	-2.857381	H	4.143399	-5.278472	-0.624934
19	H	-12.575914	4.555207	-1.484599	H	-3.786186	-1.420082	-1.906587	H	5.167143	-7.535210	-0.503723
20	H	-13.707639	6.335076	-0.233215	H	-1.846290	-1.368263	-0.517684	H	6.424442	-6.472350	-1.204120
21	H	-11.718019	8.305192	-0.359383	H	-0.134518	0.290895	-2.114873	H	6.194400	-6.682353	1.592292
22	H	-10.419312	6.882757	1.330021	H	0.537447	-1.968507	-3.172753	N	-0.206761	-3.761845	-0.421658
23	H	-12.111585	7.236326	1.767824	H	0.703475	-1.860601	-1.396213	N	1.349975	-3.699239	-2.241617
24	H	-11.229095	4.774993	0.513628	H	-1.689610	-2.893880	-2.654099	N	2.095837	-4.221397	-0.143285
25	N	-8.673894	2.052826	-0.037063	N	-3.721113	-4.888171	-5.025371	N	0.738306	-3.980515	3.186226
26	N	-9.888458	2.931890	1.798128	N	-1.923644	-5.248693	-3.485388	N	2.763528	-4.229276	2.184138

27	N	-10.134687	3.918127	-0.257793	N	-2.739048	-3.137049	-3.806460	O	4.508684	-5.714316	1.431158
28	N	-8.750000	3.401101	-3.520570	N	-5.115260	-1.542747	-5.988926	O	4.168869	-2.445038	0.414483
29	N	-10.185876	4.826703	-2.517787	N	-3.617415	-0.953470	-4.398000	O	5.794099	-3.380347	-1.028461
30	O	-10.575128	6.742337	-1.198183	O	-1.455725	-0.487018	-3.580512	O	6.739143	-6.961794	0.797505
31	O	-13.413910	5.616733	-3.097033	O	-4.038187	0.601077	-1.374992	O	-1.778566	-3.681064	1.281475
32	O	-13.585466	7.655034	-1.898071	O	-1.867433	0.753841	-0.453892	B	4.751325	-2.291443	-0.959961
33	O	-11.821272	5.275567	1.203793	O	-0.894146	-3.031594	-1.995563	O	3.697471	-2.457321	-2.056082
34	O	-7.451144	1.161527	-1.781008	O	-5.519167	-4.663705	-6.470972	O	5.292132	-0.923001	-1.211817
35	C	-14.954995	9.778170	-3.569805	C	-4.886558	4.516432	0.610632	C	5.359575	0.494149	-3.375455
36	C	-14.897271	8.573416	-4.566656	C	-3.725519	3.461704	0.719275	C	4.556037	-0.229975	-2.218921
37	C	-16.739131	12.155417	-6.682978	C	-6.748342	2.498888	-2.877566	C	8.626470	-2.300605	-2.402997
38	C	-13.424119	8.631460	-5.088649	C	-4.334486	2.304081	1.569838	C	3.773794	-1.345216	-2.968108
39	C	-12.964133	10.090843	-4.888880	C	-5.846161	2.447897	1.334829	C	4.650861	-1.638438	-4.191695
40	C	-16.743455	11.206294	-4.619441	C	-5.952499	4.255404	-1.700295	C	7.747599	-0.310545	-2.983618
41	C	-12.863111	10.906292	-6.184277	C	-6.682995	1.970940	2.515651	C	3.863700	-2.166853	-5.386073
42	C	-17.946830	11.783310	-4.205432	C	-6.419495	5.199487	-2.611199	C	8.975211	0.350832	-2.988360
43	C	-18.661168	12.639256	-5.101343	C	-7.185000	4.775149	-3.741932	C	10.167028	-0.388383	-2.727730
44	C	-17.196819	10.626339	-2.535451	C	-5.403129	6.337521	-1.072780	C	7.480385	1.888333	-3.363442
45	H	-17.057533	10.140360	-1.585593	H	-4.967515	7.119972	-0.474977	H	6.972983	2.831624	-3.523978
46	H	-14.746381	9.405669	-2.561926	H	-4.595413	5.413786	1.164209	H	4.948845	1.495258	-3.529475
47	H	-18.374161	13.409168	-7.021126	H	-7.838201	3.001464	-4.634337	H	10.704979	-2.283482	-2.011258
48	H	-16.497804	12.891861	-8.623118	H	-6.179182	0.665885	-2.442149	H	7.416821	-3.709479	-1.791141
49	H	-15.169775	11.905585	-7.927553	H	-2.886324	3.946882	1.235573	H	3.879357	0.515851	-1.775214
50	H	-15.644170	8.688161	-5.356168	H	-4.148237	2.431433	2.642573	H	2.783635	-1.033475	-3.317518
51	H	-13.352699	8.344202	-6.144303	H	-6.129173	2.040146	0.363484	H	5.526252	-2.225090	-3.907238
52	H	-12.003313	10.076143	-4.365652	H	-7.753318	2.034178	2.269202	H	4.550921	-2.399790	-6.213281
53	H	-12.780911	11.969045	-5.907615	H	-6.424655	0.932958	2.744216	H	3.326040	-3.076505	-5.100727
54	H	-11.952845	10.610598	-6.716803	H	-6.256109	3.696820	3.249415	H	3.420813	-0.314212	-5.632726
55	H	-14.822849	10.711571	-6.479991	N	-7.319676	3.364166	-3.788170	N	9.888196	-1.734343	-2.380400
56	N	-17.926924	12.771004	-6.367461	N	-6.823297	1.175223	-3.050155	N	8.422383	-3.557667	-1.982446

57	N	-16.093411	12.374901	-7.856213	N	-6.105552	2.933358	-1.765022	N	7.538814	-1.615950	-2.819118
58	N	-16.153028	11.330365	-5.827209	N	-6.057859	6.489050	-2.205912	N	8.786189	1.717230	-3.242443
59	N	-18.218390	11.398018	-2.882617	N	-5.286944	4.977672	-0.706494	N	6.785204	0.670102	-3.200882
60	N	-16.261681	10.471177	-3.571624	O	-6.029241	3.935075	1.335863	O	5.062278	-0.265584	-4.610900
61	O	-13.975138	10.759896	-4.019235	O	-6.350569	2.794755	3.681911	O	2.880448	-1.151284	-5.771914
62	O	-13.988711	10.659176	-7.087013	O	-7.715786	5.494543	-4.615148	O	11.353570	0.017220	-2.753578
63	O	-19.723303	13.262917	-4.987443	H	-7.292411	0.703285	-3.858649	H	9.117154	-4.101098	-1.432127
64	O	-5.361081	-3.572157	-11.314134	O	-3.609769	12.555005	-4.699706	C	15.488895	-5.725918	7.296520
65	O	-7.482239	-3.571045	-12.375209	O	-3.539396	11.053745	-6.545172	C	14.509832	-5.597153	6.067628
66	B	-6.712531	-2.881037	-11.305766	B	-3.154378	11.196967	-5.080473	C	14.653595	-9.985726	6.993828
67	C	-5.520972	-1.809863	-8.752031	C	-2.541982	8.264716	-5.436025	C	15.452816	-5.708836	4.832381
68	C	-6.976002	-1.710867	-9.316510	C	-2.768549	9.073876	-4.111213	C	16.647189	-6.518904	5.357091
69	C	-6.866196	0.597534	-5.344056	C	-2.376275	4.856431	-2.734899	C	14.939147	-8.066804	8.125726
70	C	-6.830108	-0.631643	-10.427793	C	-1.445381	9.856833	-4.004973	C	17.975433	-6.212247	4.671121
71	C	-5.644483	0.246897	-9.998383	C	-0.383084	8.925116	-4.586871	C	14.608139	-8.593616	9.381536
72	C	-5.910894	-1.232708	-6.312986	C	-3.033650	5.924353	-4.620235	C	14.309993	-9.987471	9.501878
73	C	-6.075255	1.576841	-9.367166	C	0.318031	8.084276	-3.506847	C	14.911156	-6.475216	9.680742
74	C	-5.619163	-1.847136	-5.099384	C	-3.756077	4.837874	-5.120633	H	14.995883	-5.482249	10.090244
75	C	-5.982716	-1.196413	-3.882919	C	-3.801726	3.623009	-4.365735	H	15.552064	-4.767724	7.819973
76	C	-4.903837	-3.183670	-6.654653	C	-4.024736	6.451988	-6.537145	H	14.027491	-11.580875	8.200134
77	H	-4.479848	-4.005884	-7.203346	H	-4.323924	7.069619	-7.366266	H	14.070111	-11.530531	5.764206
78	H	-5.003100	-2.621162	-9.273431	H	-2.894567	8.865283	-6.279435	H	14.276283	-9.897385	5.048352
79	H	-6.932852	0.552522	-3.230488	H	-3.337385	3.083088	-2.369245	H	14.024803	-4.612653	6.122654
80	H	-7.855597	2.369913	-4.713552	H	-1.842358	3.988093	-0.974479	H	15.789659	-4.740383	4.449166
81	H	-7.540043	2.098622	-6.460269	H	-1.077209	5.592828	-1.371776	H	16.389392	-7.577040	5.384083
82	H	-7.684882	-1.438617	-8.533876	H	-2.975860	8.419036	-3.267990	H	18.747637	-6.916064	5.014220
83	H	-7.740025	-0.033067	-10.538582	H	-1.213352	10.140353	-2.971101	H	17.859124	-6.301630	3.588316
84	H	-4.984403	0.413013	-10.855840	H	0.345520	9.500257	-5.168841	H	17.999293	-4.720863	5.910481
85	H	-5.200061	1.991062	-8.839570	H	0.821390	7.246530	-4.018961	N	14.336171	-10.611736	8.191889
86	H	-6.355822	2.270890	-10.168893	H	1.087549	8.706594	-3.028716	N	14.500974	-10.613160	5.802034

87	H	-6.961770	0.721654	-7.793011	H	-1.303633	7.057536	-2.926233	N	15.071159	-8.727898	6.966309
88	N	-6.620695	0.052932	-4.103986	N	-3.130587	3.774515	-3.106260	N	14.602100	-7.571953	10.347100
89	N	-7.441722	1.797417	-5.484588	N	-1.671887	4.793912	-1.584632	N	15.120254	-6.712796	8.307324
90	N	-6.525117	-0.052870	-6.478886	N	-2.298086	5.957812	-3.483301	O	16.825239	-6.004229	6.754579
91	N	-4.983519	-3.074692	-5.333605	N	-4.362021	5.192367	-6.334678	O	13.550452	-6.641871	5.890315
92	N	-5.466741	-2.082804	-7.305200	N	-3.209675	6.963374	-5.499692	O	14.716404	-6.442792	3.839113
93	O	-4.884889	-0.518710	-8.965555	O	-1.098195	8.004038	-5.508528	O	18.359294	-4.832285	4.980351
94	O	-7.358794	-2.925158	-9.982162	O	-3.774150	10.097348	-4.245103	O	14.026830	-10.687055	10.488511
95	O	-6.524471	-1.413969	-11.608261	O	-1.687322	10.997143	-4.868149	B	13.501392	-7.089402	4.459851
96	O	-7.234271	1.426642	-8.505797	O	-0.583113	7.645178	-2.457460	O	13.552843	-8.613418	4.326206
97	O	-5.802045	-1.593343	-2.709736	O	-4.315767	2.516438	-4.630966	O	12.219145	-6.734511	3.782638
98	C	-6.007983	-2.846098	-14.223743	C	-5.424977	12.305927	-7.823025	C	10.888266	-7.962174	1.938818
99	C	-6.509982	-4.021066	-13.319396	C	-4.036128	12.285908	-7.056239	C	11.467994	-7.889743	3.408173
100	C	-5.531005	-5.664125	-17.439306	C	-6.528815	8.881047	-5.283777	C	14.024265	-5.065576	0.876523
101	C	-5.230619	-4.380383	-12.499076	C	-4.297858	13.172735	-5.788477	C	12.500003	-9.049954	3.444502
102	C	-4.037490	-3.935447	-13.368968	C	-5.822686	13.140032	-5.616485	C	12.955917	-9.163847	1.985226
103	C	-6.411648	-3.779375	-16.526186	C	-6.416111	10.032146	-7.220369	C	11.952123	-5.943231	0.809941
104	C	-3.295223	-5.088703	-14.057321	C	-6.390123	14.417000	-5.005063	C	13.440305	-10.559582	1.612279
105	C	-7.077615	-3.364362	-17.682021	C	-7.105032	9.070750	-7.962485	C	11.440983	-4.949076	-0.023515
106	C	-6.992490	-4.161754	-18.866495	C	-7.568748	7.891376	-7.305159	C	12.315751	-3.935809	-0.516898
107	C	-7.430819	-1.851135	-16.173366	C	-6.594779	10.643527	-9.363244	C	9.742449	-6.206977	0.499662
108	H	-7.755529	-0.983692	-15.625679	H	-6.478090	11.256873	-10.240366	H	8.746305	-6.613517	0.622983
109	H	-6.227695	-1.899465	-13.719713	H	-5.299763	12.807784	-8.786875	H	9.854450	-8.315742	1.973747
110	H	-6.017606	-5.911798	-19.455546	H	-7.412652	6.971462	-5.428635	H	14.263579	-3.239299	-0.158088
111	H	-4.615426	-7.452738	-18.016499	H	-5.364589	9.411765	-3.802089	H	15.257518	-5.715492	2.246044
112	H	-4.270069	-6.820586	-16.372939	H	-3.304979	12.763191	-7.729100	H	10.632398	-8.062043	4.103140
113	H	-6.888306	-4.846333	-13.927124	H	-3.993067	14.215660	-5.939977	H	12.079643	-10.012241	3.754934
114	H	-5.172683	-5.453026	-12.278425	H	-6.123611	12.212151	-5.129798	H	13.629277	-8.345163	1.725901
115	H	-3.352510	-3.354669	-12.743631	H	-7.471299	14.303919	-4.830186	H	13.795485	-10.567852	0.571143
116	H	-2.649844	-4.657715	-14.838544	H	-5.888540	14.612658	-4.052682	H	14.262072	-10.851156	2.273856

117	H	-2.664111	-5.593666	-13.318107	H	-6.236638	15.073203	-6.802057	H	11.560727	-10.941681	1.487757
118	H	-4.923559	-5.545713	-15.108028	N	-7.203753	7.863156	-5.935759	N	13.615716	-4.042508	0.040577
119	N	-6.144174	-5.338143	-18.625268	N	-6.153130	8.765021	-4.004527	N	15.236808	-5.056607	1.449030
120	N	-4.718245	-6.742861	-17.306272	N	-6.189545	10.027090	-5.910298	N	13.220123	-6.106584	1.182891
121	N	-5.690042	-4.908099	-16.362890	N	-7.221992	9.482532	-9.300432	N	10.064417	-5.140453	-0.213922
122	N	-7.722296	-2.141225	-17.434536	N	-6.056127	11.029403	-8.118868	N	10.868761	-6.739995	1.163478
123	N	-6.629582	-2.831925	-15.565334	O	-6.320816	13.166286	-7.029493	O	11.663838	-9.018279	1.248909
124	O	-4.578872	-3.047547	-14.439813	O	-6.108212	15.530120	-5.915361	O	12.332865	-11.499458	1.811063
125	O	-4.213536	-6.093687	-14.594602	O	-8.209144	6.932843	-7.795576	O	12.055082	-3.013418	-1.325771
126	O	-7.473926	-4.024155	-19.997602	H	-6.271361	7.913782	-3.431703	H	15.860593	-4.225140	1.477528
127	O	-2.345548	-10.723208	2.200979	O	-9.410615	-3.631267	-10.660234	C	14.628778	7.140462	-2.624445
128	O	-3.468671	-12.448816	1.032218	O	-7.709310	-2.409198	-9.542484	C	13.272615	7.858971	-2.242807
129	B	-3.087423	-11.014255	0.912412	B	-8.367782	-2.595871	-10.877082	C	12.190221	3.537177	-3.283916
130	C	-2.927753	-7.977865	0.756380	C	-7.141437	0.319481	-12.062611	C	12.791025	8.456961	-3.592851
131	C	-3.706332	-9.019351	-0.112804	C	-8.109816	-0.820939	-12.518589	C	13.389206	7.507190	-4.636173
132	C	-4.316993	-4.838465	-1.971246	C	-6.613578	1.700168	-16.136792	C	14.000061	4.673855	-2.575210
133	C	-2.586032	-9.631243	-1.008440	C	-7.178767	-2.020529	-12.915080	C	13.627813	8.169654	-5.987774
134	C	-1.478217	-8.567565	-1.077840	C	-5.731525	-1.465458	-12.868871	C	14.639915	3.546547	-2.061818
135	C	-4.065899	-5.814208	0.075984	C	-7.255978	1.965765	-13.969929	C	14.036435	2.264972	-2.231930
136	C	-1.461345	-7.801569	-2.408549	C	-4.992801	-1.569482	-14.212447	C	15.904197	5.235840	-1.523165
137	C	-4.680259	-4.769506	0.759434	C	-7.534326	3.324353	-14.097245	H	16.668109	5.866718	-1.085685
138	C	-5.167990	-3.647552	0.025116	C	-7.398236	3.969134	-15.360670	H	15.466682	7.689103	-2.186887
139	C	-4.085448	-6.220952	2.260920	C	-7.821760	2.837721	-12.003436	H	12.191039	1.470140	-2.839397
140	H	-3.910841	-6.753251	3.179134	H	-8.055497	2.893429	-10.951972	H	10.237991	2.814653	-3.590426
141	H	-2.684285	-8.445634	1.715647	H	-7.192390	0.411684	-10.975253	H	10.590341	4.553953	-3.765319
142	H	-5.254367	-2.943882	-1.946142	H	-6.714147	3.459385	-17.260810	H	13.508440	8.644200	-1.508595
143	H	-4.440158	-4.173198	-3.986682	H	-5.815252	1.270771	-18.013758	H	13.158715	9.469893	-3.788412
144	H	-3.593757	-5.711213	-3.605743	H	-5.765554	-0.023052	-16.772977	H	12.837918	6.565727	-4.660497
145	H	-4.502769	-8.536421	-0.680691	H	-8.722285	-0.468502	-13.354977	H	14.002614	7.428161	-6.709280
146	H	-2.953394	-9.871545	-2.010901	H	-7.394946	-2.372293	-13.929939	H	12.687863	8.587357	-6.362525

147	H	-0.512692	-9.045882	-0.885142	H	-5.194678	-2.001084	-12.079823	H	15.202885	8.856459	-5.127776
148	H	-0.892567	-6.871658	-2.248411	H	-4.107070	-0.917965	-14.161456	N	12.750309	2.360221	-2.822539
149	H	-0.933714	-8.407951	-3.154802	H	-4.663579	-2.605044	-14.356134	N	10.929621	3.576984	-3.739352
150	H	-3.284018	-7.033878	-2.171022	H	-6.380051	-0.376919	-15.017960	N	12.866568	4.706276	-3.273627
151	N	-4.930150	-3.764329	-1.369165	N	-6.891403	3.025217	-16.358248	N	15.830782	3.918759	-1.420406
152	N	-4.102165	-4.876519	-3.291223	N	-6.062863	0.913765	-17.102899	N	14.793591	5.759575	-2.220475
153	N	-3.892112	-5.901625	-1.250599	N	-6.849455	1.132381	-14.961221	O	14.762658	7.281215	-4.092446
154	N	-4.685657	-5.042958	2.134815	N	-7.885437	3.855253	-12.852678	O	12.180981	7.051898	-1.801119
155	N	-3.691900	-6.744214	1.025193	N	-7.449465	1.640585	-12.650136	O	11.351209	8.405911	-3.555826
156	O	-1.738930	-7.587362	0.016434	O	-5.806879	-0.022884	-12.539230	O	14.578297	9.269156	-5.799541
157	O	-4.214352	-10.094072	0.695148	O	-8.927026	-1.301285	-11.440898	O	14.495239	1.141937	-1.914004
158	O	-2.168581	-10.803421	-0.270431	O	-7.398662	-3.032860	-11.933595	B	10.904233	7.403893	-2.491953
159	O	-2.799481	-7.562783	-2.924187	O	-5.866327	-1.228053	-15.334238	O	10.199879	6.221362	-3.110861
160	O	-5.742038	-2.625772	0.464222	O	-7.609988	5.133857	-15.718766	O	9.881805	7.979658	-1.556428
161	C	-1.158781	-13.321345	0.855418	C	-8.452674	-3.262981	-7.202085	C	7.339673	7.690943	-1.465151
162	C	-2.402039	-13.054197	1.767010	C	-8.011253	-3.519402	-8.698830	C	8.789782	7.076213	-1.377761
163	C	-0.082246	-16.704477	3.294452	C	-9.737470	0.676167	-8.844402	C	9.247594	10.778851	-3.880157
164	C	-1.880778	-11.962643	2.767543	C	-9.274531	-4.189208	-9.351212	C	8.858740	6.116846	-2.603801
165	C	-0.339683	-12.079617	2.750966	C	-10.428175	-3.789097	-8.422181	C	7.836226	6.706437	-3.586895
166	C	-0.667956	-15.750023	1.318576	C	-8.929733	-0.725955	-7.278574	C	7.852966	10.051308	-2.275931
167	C	0.262603	-12.676044	4.030696	C	-11.554620	-4.817582	-8.357542	C	7.181189	5.685626	-4.511238
168	C	-0.525171	-16.912770	0.556639	C	-8.653658	0.290194	-6.360771	C	7.474805	11.325526	-1.830960
169	C	-0.128240	-18.128417	1.196855	C	-8.942819	1.641912	-6.701591	C	7.983931	12.483030	-2.500074
170	C	-1.068506	-15.325629	-0.812583	C	-7.954100	-1.539441	-5.444471	C	6.522917	9.918185	-0.496720
171	H	-1.325012	-14.741895	-1.679418	H	-7.505329	-2.268755	-4.795952	H	5.956237	9.446841	0.289129
172	H	-1.252178	-12.706867	-0.044687	H	-7.780635	-3.825198	-6.552719	H	6.732773	7.331709	-0.629311
173	H	0.421953	-18.724120	3.120598	H	-9.434977	2.706334	-8.443475	H	9.394968	12.867375	-3.973423
174	H	0.418202	-17.330674	5.223634	H	-9.982370	0.052145	-10.710855	H	10.695695	9.575324	-4.503316
175	H	0.093133	-15.583868	4.962620	H	-7.161091	-4.212477	-8.636534	H	8.869271	6.530297	-0.427308
176	H	-2.723523	-13.975418	2.260298	H	-9.203626	-5.284552	-9.360530	H	8.597470	5.081294	-2.363936

177	H	-2.264327	-12.125172	3.781585	H	-10.742709	-2.768269	-8.648719	H	8.274011	7.562066	-4.100351
178	H	0.075486	-11.086219	2.555649	H	-12.387159	-4.427167	-7.750165	H	6.560394	6.196542	-5.261151
179	H	1.310786	-12.938347	3.817771	H	-11.913803	-5.022638	-9.370765	H	7.953705	5.101143	-5.016547
180	H	0.243506	-11.917319	4.820389	H	-10.345774	-5.673816	-7.140672	H	6.039861	5.369356	-2.972560
181	H	-0.676127	-14.396726	3.674980	N	-9.463931	1.753701	-8.013874	N	8.919428	12.082771	-3.534114
182	N	0.089656	-17.896514	2.631575	N	-10.147491	0.877586	-10.108424	N	10.247448	10.506373	-4.753931
183	N	0.194427	-16.557281	4.614898	N	-9.559829	-0.597088	-8.449127	N	8.627601	9.745517	-3.327752
184	N	-0.506757	-15.625486	2.652991	N	-8.051774	-0.244443	-5.211093	N	6.631929	11.215965	-0.711490
185	N	-0.790031	-16.622519	-0.791480	N	-8.458003	-1.899007	-6.698372	N	7.259490	9.148582	-1.419739
186	N	-1.012852	-14.740762	0.463147	O	-9.796217	-3.865954	-7.068504	O	6.725737	7.166596	-2.690743
187	O	0.031547	-12.989993	1.630129	O	-11.014224	-6.054287	-7.790652	O	6.369592	4.769047	-3.705408
188	O	-0.502424	-13.824873	4.518080	O	-8.756434	2.670063	-6.003700	O	7.777516	13.694814	-2.321222
189	O	0.066541	-19.271400	0.765481	H	-10.502620	1.769368	-10.485476	H	10.826769	11.258638	-5.110693
190	B	-10.092027	-0.719585	9.778086	B	-11.469525	8.593298	-14.515791	C	19.957834	-1.117564	3.201523
191	O	-9.990623	0.788931	9.902286	O	-12.748242	9.103628	-13.966292	C	19.967618	0.312319	3.879469
192	C	-10.637651	1.220521	11.116028	C	-13.691044	8.038960	-13.834373	H	20.136511	-1.880985	3.963283
193	O	-11.139035	-1.095761	10.766261	O	-11.618894	7.087731	-14.537672	N	18.752666	-1.519107	2.507507
194	C	-11.031951	-0.123221	11.809734	C	-12.985939	6.741108	-14.375708	O	21.133181	-1.148036	2.302050
195	O	-10.372699	-1.152715	8.398298	O	-10.252478	9.032669	-13.737699	C	21.279253	0.952209	3.349226
196	O	-8.760568	-1.347021	10.121994	O	-11.216152	9.130245	-15.893397	H	19.993983	0.161662	4.969344
197	C	-9.094348	-1.395229	7.787517	C	-9.193462	9.287031	-14.677503	O	18.947603	1.238716	3.508086
198	C	-8.417991	-0.060494	7.333907	C	-8.404970	7.996936	-15.074586	N	16.483627	0.049047	-0.403133
199	H	-8.912570	0.768300	7.850331	H	-8.969898	7.099779	-14.819218	C	17.577454	0.780505	0.022596
200	N	-8.504908	0.182466	5.880815	N	-7.077448	7.908158	-14.439545	N	17.764469	2.047133	-0.377413
201	O	-7.004890	-0.149073	7.664089	O	-8.148768	8.098170	-16.515325	N	18.491672	0.274512	0.878213
202	C	-8.151879	-1.889734	8.926987	C	-9.905341	9.705823	-15.998575	C	21.484514	0.269829	1.991132
203	H	-9.173509	-2.097697	6.956539	H	-8.506740	10.047852	-14.298105	H	22.155723	0.757135	3.975991
204	N	-6.813860	-1.652243	2.731568	N	-3.741740	9.946418	-15.236198	O	21.010663	2.362820	3.227745
205	C	-6.614193	-2.220179	3.967946	C	-4.899429	10.390110	-15.819413	C	22.948479	0.208145	1.569540
206	N	-5.860373	-3.315698	4.124323	N	-4.905430	11.410263	-16.725559	H	20.796301	0.673669	1.246206

207	N	-7.161603	-1.687384	5.083961	N	-6.062722	9.814463	-15.556424	C	17.053726	-1.688049	1.083249
208	C	-6.781435	-1.254842	8.641613	C	-9.102382	9.045669	-17.135653	C	18.128496	-0.875737	1.444205
209	H	-8.076636	-2.981590	8.947856	H	-9.924073	10.796250	-16.120965	H	23.035842	-0.246041	0.571360
210	C	-5.774283	-2.236944	8.028192	C	-8.286113	10.034341	-17.979758	H	23.361804	1.221282	1.538877
211	H	-6.390063	-0.819046	9.566359	H	-9.808499	8.495236	-17.767245	O	23.684837	-0.558311	2.577911
212	C	-8.126935	0.066919	3.692398	C	-4.892434	8.189332	-14.122872	C	16.172385	-1.259250	0.046783
213	C	-7.870858	-0.562785	4.906637	C	-6.018264	8.713784	-14.761229	N	17.028194	-2.838649	1.884648
214	H	-4.966627	-1.642530	7.571481	H	-7.554880	9.451236	-18.562238	O	15.201973	-1.878041	-0.451290
215	H	-5.344297	-2.844857	8.833778	H	-8.960505	10.548642	-18.675734	C	18.042246	-2.719684	2.725886
216	O	-6.397045	-3.155802	7.089463	O	-7.646170	11.061776	-17.160560	H	18.294147	-3.411503	3.520194
217	C	-7.576793	-0.479199	2.494654	C	-3.615077	8.797510	-14.320528	H	15.766961	0.492422	-1.031158
218	N	-8.919501	1.204018	3.906353	N	-5.256667	7.047016	-13.395686	H	17.032218	2.620148	-0.841881
219	O	-7.685341	-0.045145	1.325999	O	-2.482159	8.529233	-13.906261	H	18.428767	2.524503	0.256075
220	H	-9.692518	1.987248	5.751673	H	-7.174006	6.095846	-13.220066	H	23.013703	-1.277450	2.786812
221	C	-9.120858	1.248507	5.218306	C	-6.555437	6.892193	-13.606615	B	19.497961	2.590973	3.196588
222	H	-6.369990	-2.076388	1.874641	H	-2.846420	10.377859	-15.451498	O	19.053441	3.132588	1.860012
223	H	-5.443633	-3.885786	3.355572	H	-4.117461	12.027260	-16.855589	O	19.056980	3.642127	4.171902
224	H	-5.749959	-3.608732	5.101829	H	-5.827204	11.575408	-17.169584	C	18.443054	4.424119	2.022357
225	H	-6.840657	-2.544733	6.374676	H	-7.197504	10.524908	-16.381923	C	18.173449	4.578034	3.549455
226	C	-9.795063	-0.402622	12.726765	C	-13.366671	5.627043	-13.320797	C	18.448908	6.106834	3.823032
227	H	-9.096461	-1.044375	12.181038	H	-13.909649	4.815316	-13.812813	H	17.510817	6.614466	4.064283
228	N	-10.144364	-1.050124	14.009944	N	-12.288759	4.993278	-12.578884	N	19.361520	6.403773	4.924512
229	O	-9.212935	0.891282	13.070470	O	-14.331762	6.256379	-12.400883	O	18.937350	6.685539	2.565895
230	H	-11.949625	-0.059358	12.399651	H	-13.441221	6.426464	-15.330456	H	17.124463	4.370257	3.802049
231	N	-12.042971	0.429796	17.185976	N	-9.384384	6.237020	-10.092706	N	23.084896	5.303010	5.794865
232	C	-11.976455	1.221473	16.064557	C	-9.959201	7.182305	-10.927900	C	22.525697	4.804851	4.625822
233	N	-12.452901	2.492209	16.034323	N	-9.459116	8.422000	-10.996016	N	23.127612	3.821656	3.912597
234	N	-11.410145	0.776793	14.952363	N	-11.013343	6.890930	-11.717682	N	21.351965	5.237795	4.188270
235	C	-9.652305	1.914499	12.077458	C	-14.012056	7.722219	-12.369180	C	19.435727	5.555625	1.716343
236	H	-11.510269	1.856872	10.925016	H	-14.635735	8.233354	-14.359044	H	17.545360	4.524481	1.403860

237	C	-10.250772	3.091005	12.860626	C	-15.303564	8.359968	-11.867540	C	19.379748	6.095413	0.291866
238	H	-8.781216	2.240155	11.500726	H	-13.129872	7.913012	-11.757569	H	20.433232	5.261429	2.041643
239	C	-10.862061	-1.326923	16.101265	C	-10.714523	4.561292	-11.066975	C	21.130062	6.534540	6.270208
240	C	-10.842619	-0.445424	15.017695	C	-11.311848	5.595378	-11.788354	C	20.670507	5.995000	5.060900
241	H	-9.554068	3.340657	13.676018	H	-15.430470	8.154626	-10.793491	H	20.203622	6.804741	0.126844
242	H	-10.332495	3.954667	12.191554	H	-15.260383	9.441516	-12.025082	H	19.468307	5.267584	-0.416189
243	O	-11.597296	2.803011	13.355391	O	-16.419530	7.822227	-12.650881	O	18.085961	6.748450	0.076122
244	C	-11.504322	-0.932194	17.316617	C	-9.688431	4.867665	-10.133757	C	22.457060	6.235150	6.713070
245	N	-10.167875	-2.499425	15.760804	N	-11.292933	3.330370	-11.397400	N	20.114754	7.294918	6.876045
246	O	-11.657511	-1.508541	18.400455	O	-9.061489	4.052344	-9.400123	O	23.096356	6.608614	7.710552
247	H	-9.179802	-2.986353	13.917223	H	-12.890565	2.912009	-12.760955	H	18.116402	7.649922	6.191249
248	C	-9.749637	-2.299193	14.518047	C	-12.229958	3.615152	-12.282403	C	19.085498	7.199388	6.054893
249	H	-12.467789	0.771381	18.044650	H	-8.757360	6.539671	-9.302031	H	23.991254	4.971183	6.116456
250	H	-12.999027	2.898087	16.779742	H	-9.782434	8.928586	-11.836199	H	22.389735	3.232321	3.424014
251	H	-12.266606	2.991013	15.142714	H	-16.116283	6.870168	-12.763492	H	17.886115	7.125451	0.984024
252	H	-11.517185	1.867577	13.789086	H	-8.634985	8.729772	-10.452201	H	23.966647	3.378184	4.269744

S5. Sβ-G4 with K^+

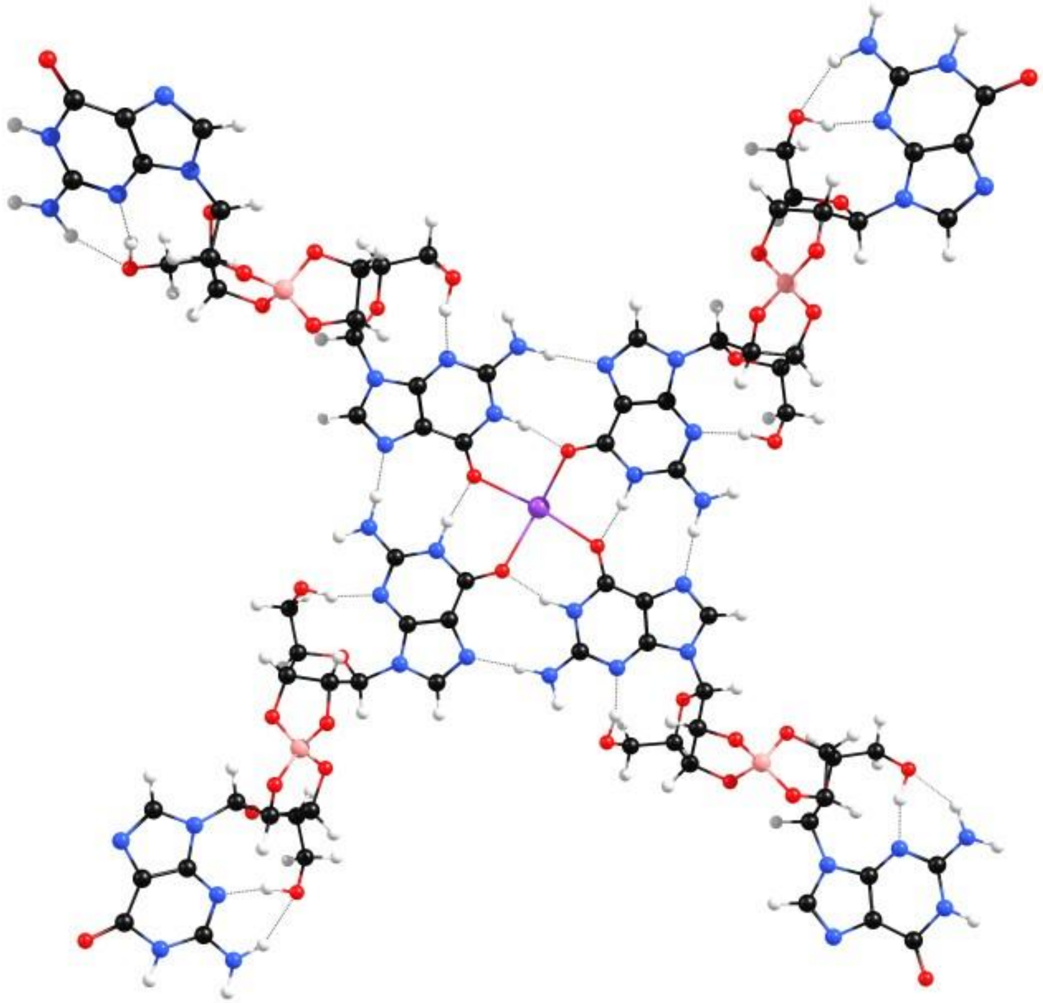

Figure S37. Optimized geometry of **Sβ-G4**, formed by four borate-guanosine diesters with two β-guanosines in syn conformation coordinated to K^+.

Table S17. Optimized xyz cartesian coordinates for **Sβ-G4** quartet with K^+.

Number	Atom	x	y	z
1	O	-12.764393	7.636379	-3.964731
2	O	-15.070349	7.189838	-3.663388
3	B	-13.747702	6.916740	-3.060953
4	C	-11.077065	5.888321	-2.221511
5	C	-12.509482	5.413367	-1.812295
6	C	-9.337308	3.030837	0.584199
7	C	-12.954279	6.524654	-0.811068
8	C	-11.653550	7.111997	-0.231599
9	C	-9.641190	3.937442	-1.481695
10	C	-11.339921	6.642339	1.194297
11	C	-8.711523	3.097697	-2.102476
12	C	-8.015745	2.153210	-1.313376
13	C	-9.496271	4.412739	-3.642531
14	H	-9.714631	4.908262	-4.571954
15	H	-11.153346	6.475988	-3.141676
16	H	-7.965931	1.430993	0.653566
17	H	-9.211116	2.368413	2.591064
18	H	-10.316602	3.730733	2.165049
19	H	-12.484227	4.414522	-1.372469
20	H	-13.589861	6.125763	-0.013469
21	H	-11.705704	8.203724	-0.280148
22	H	-10.289507	6.895899	1.408243
23	H	-11.979011	7.192158	1.894070
24	H	-11.107666	4.749326	0.635450
25	N	-8.398398	2.167277	0.044768
26	N	-9.622289	3.023398	1.887938
27	N	-9.985632	3.919733	-0.191869

28	N	-8.640423	3.410894	-3.469471
29	N	-10.135700	4.770613	-2.456438
30	O	-10.535506	6.653988	-1.112237
31	O	-13.410280	5.483796	-2.924718
32	O	-13.645424	7.471419	-1.655876
33	O	-11.622360	5.225547	1.386281
34	O	-7.105674	1.345724	-1.690695
35	C	-15.042974	9.629719	-3.240664
36	C	-14.948654	8.472581	-4.289176
37	C	-16.818404	12.137048	-6.259673
38	C	-13.472200	8.580977	-4.793781
39	C	-13.036619	10.033889	-4.512145
40	C	-16.834564	11.084865	-4.246708
41	C	-12.933416	10.912345	-5.765691
42	C	-18.047695	11.627735	-3.818136
43	C	-18.762427	12.520623	-4.677784
44	C	-17.305836	10.397971	-2.197614
45	H	-17.172153	9.868244	-1.270603
46	H	-14.845950	9.216518	-2.246902
47	H	-18.461640	13.389154	-6.553872
48	H	-16.560265	12.981469	-8.153110
49	H	-15.236683	11.964650	-7.501597
50	H	-15.690080	8.603925	-5.080323
51	H	-13.385936	8.350920	-5.861227
52	H	-12.082010	10.008703	-3.977679
53	H	-12.876835	11.961290	-5.436102
54	H	-12.009755	10.660497	-6.297743
55	H	-14.887587	10.711523	-6.098873
56	N	-18.014684	12.724021	-5.927246
57	N	-16.164575	12.418904	-7.414226

58	N	-16.231231	11.275222	-5.439564
59	N	-18.330113	11.175074	-2.519457
60	N	-16.357226	10.302347	-3.230333
61	O	-14.068252	10.643203	-3.625816
62	O	-14.040731	10.683817	-6.693265
63	O	-19.832174	13.124501	-4.543631
64	O	-5.371153	-3.483418	-11.248895
65	O	-7.477792	-3.672791	-12.320629
66	B	-6.772885	-2.903197	-11.273063
67	C	-5.595123	-1.744715	-8.789887
68	C	-7.072029	-1.724547	-9.301931
69	C	-6.845151	0.677115	-5.386541
70	C	-7.008825	-0.648894	-10.428206
71	C	-5.852155	0.290913	-10.043891
72	C	-5.902613	-1.161475	-6.348951
73	C	-6.312565	1.624193	-9.444027
74	C	-5.529193	-1.738171	-5.132166
75	C	-5.825452	-1.046903	-3.937172
76	C	-4.857422	-3.085299	-6.697105
77	H	-4.439423	-3.907618	-7.250385
78	H	-5.046937	-2.520833	-9.333690
79	H	-6.783378	0.698789	-3.275626
80	H	-7.824944	2.440061	-4.738315
81	H	-7.605633	2.129515	-6.504839
82	H	-7.768197	-1.476430	-8.498995
83	H	-7.949425	-0.095364	-10.519539
84	H	-5.214767	0.453507	-10.918122
85	H	-5.435262	2.108219	-8.985444
86	H	-6.678274	2.265846	-10.253421
87	H	-7.092706	0.740303	-7.839455

88	N	-6.508390	0.172124	-4.139295
89	N	-7.447263	1.858611	-5.524124
90	N	-6.554626	-0.008429	-6.508583
91	N	-4.870798	-2.955440	-5.374292
92	N	-5.480198	-2.015131	-7.339506
93	O	-5.047688	-0.414514	-8.998124
94	O	-7.414021	-2.960043	-9.940959
95	O	-6.699186	-1.428601	-11.605117
96	O	-7.413651	1.453301	-8.506430
97	O	-5.525144	-1.392836	-2.749348
98	C	-6.032321	-2.899487	-14.174589
99	C	-6.464160	-4.081842	-13.244215
100	C	-5.408130	-5.773350	-17.315943
101	C	-5.174122	-4.329216	-12.403247
102	C	-4.004820	-3.844743	-13.281362
103	C	-6.378868	-3.910294	-16.451912
104	C	-3.209400	-4.981162	-13.939792
105	C	-7.067334	-3.560928	-17.614770
106	C	-6.946774	-4.384947	-18.777778
107	C	-7.499799	-2.028961	-16.146575
108	H	-7.869441	-1.165427	-15.621749
109	H	-6.310830	-1.956623	-13.693740
110	H	-5.891694	-6.105279	-19.319735
111	H	-4.400735	-7.524501	-17.853134
112	H	-4.096681	-6.847732	-16.223681
113	H	-6.778603	-4.947676	-13.829741
114	H	-5.052482	-5.385394	-12.137040
115	H	-3.349485	-3.211204	-12.675783
116	H	-2.588618	-4.541044	-14.735667
117	H	-2.551592	-5.434033	-13.190171

118	H	-4.802384	-5.547170	-14.994082
119	N	-6.040607	-5.512369	-18.506637
120	N	-4.548295	-6.810333	-17.155186
121	N	-5.597637	-4.993795	-16.260112
122	N	-7.775241	-2.367677	-17.398073
123	N	-6.647240	-2.950656	-15.514532
124	O	-4.591775	-3.017315	-14.375870
125	O	-4.081732	-6.041573	-14.440008
126	O	-7.438249	-4.302471	-19.908475
127	O	-2.404722	-10.663945	1.950944
128	O	-3.464936	-12.487852	0.873738
129	B	-3.123036	-11.062337	0.675822
130	C	-2.955324	-8.082008	0.518396
131	C	-3.739326	-9.095925	-0.379124
132	C	-4.195892	-4.857174	-2.143373
133	C	-2.604002	-9.706609	-1.260125
134	C	-1.491387	-8.645497	-1.309332
135	C	-4.037343	-5.885858	-0.114788
136	C	-1.428349	-7.874320	-2.633795
137	C	-4.612077	-4.822706	0.586542
138	C	-4.986386	-3.665213	-0.127630
139	C	-4.106225	-6.336942	2.055626
140	H	-3.970721	-6.900357	2.961930
141	H	-2.706975	-8.572576	1.464611
142	H	-5.040341	-2.923775	-2.089511
143	H	-4.277994	-4.130725	-4.130399
144	H	-3.478777	-5.715849	-3.784145
145	H	-4.518891	-8.592605	-0.953842
146	H	-2.954359	-9.951198	-2.268224
147	H	-0.533833	-9.128156	-1.092748

148	H	-0.826992	-6.967246	-2.464125
149	H	-0.919513	-8.495399	-3.378933
150	H	-3.244341	-7.106405	-2.396941
151	N	-4.758830	-3.755474	-1.517323
152	N	-3.958703	-4.865362	-3.455609
153	N	-3.846748	-5.948865	-1.434967
154	N	-4.651846	-5.128917	1.956800
155	N	-3.715915	-6.845655	0.814729
156	O	-1.776005	-7.662126	-0.220014
157	O	-4.272144	-10.173523	0.401532
158	O	-2.200332	-10.873658	-0.510050
159	O	-2.752557	-7.575328	-3.167821
160	O	-5.451854	-2.580280	0.348218
161	C	-1.111139	-13.256495	0.901875
162	C	-2.426052	-13.010595	1.712867
163	C	-0.204322	-16.569802	3.500825
164	C	-2.017760	-11.850511	2.677307
165	C	-0.488097	-11.954260	2.833408
166	C	-0.596399	-15.658720	1.456881
167	C	-0.035250	-12.544789	4.176578
168	C	-0.328471	-16.822314	0.733778
169	C	0.038054	-18.015909	1.432396
170	C	-0.776683	-15.274539	-0.712858
171	H	-0.957678	-14.711183	-1.611641
172	H	-1.158520	-12.668897	-0.019448
173	H	0.384051	-18.571324	3.415962
174	H	0.092911	-17.153652	5.485126
175	H	-0.278239	-15.430132	5.162625
176	H	-2.745377	-13.918098	2.228947
177	H	-2.515445	-11.930589	3.649767

178	H	-0.051964	-10.961664	2.685012
179	H	1.024566	-12.826458	4.078375
180	H	-0.124583	-11.775044	4.950840
181	H	-0.908664	-14.285010	3.759955
182	N	0.083814	-17.760472	2.880099
183	N	-0.095411	-16.398774	4.842103
184	N	-0.586530	-15.511719	2.798280
185	N	-0.453867	-16.557921	-0.639689
186	N	-0.885438	-14.672978	0.553228
187	O	0.004282	-12.859542	1.755846
188	O	-0.871124	-13.669780	4.592205
189	O	0.319516	-19.153608	1.040600
190	B	-10.186577	-0.802666	9.549827
191	O	-9.902073	0.687021	9.604038
192	C	-10.510676	1.253929	10.782872
193	O	-11.288450	-1.001831	10.516403
194	C	-11.110176	0.009451	11.515554
195	O	-10.475539	-1.244073	8.168542
196	O	-8.947065	-1.573395	9.949277
197	C	-9.197917	-1.576428	7.608244
198	C	-8.391620	-0.287954	7.238196
199	H	-8.813386	0.557827	7.789852
200	N	-8.410304	0.036064	5.794179
201	O	-6.998024	-0.531324	7.577872
202	C	-8.348496	-2.168867	8.775326
203	H	-9.293097	-2.246348	6.751772
204	N	-6.601638	-1.699681	2.667953
205	C	-6.559659	-2.375516	3.877082
206	N	-5.899988	-3.528869	4.011064
207	N	-7.167096	-1.874496	4.969407

208	C	-6.913348	-1.665886	8.546418
209	H	-8.380972	-3.263397	8.792218
210	C	-5.974252	-2.721542	7.948930
211	H	-6.514883	-1.283168	9.490666
212	C	-7.874942	0.039923	3.634338
213	C	-7.770998	-0.692992	4.820196
214	H	-5.082121	-2.199675	7.568089
215	H	-5.662311	-3.407035	8.744604
216	O	-6.622672	-3.533150	6.924552
217	C	-7.233287	-0.450817	2.475918
218	N	-8.594818	1.222037	3.875558
219	O	-7.142155	0.121705	1.341525
220	H	-9.445403	1.932299	5.715000
221	C	-8.890727	1.188624	5.170538
222	H	-6.150374	-2.132707	1.826272
223	H	-5.443746	-4.065647	3.239619
224	H	-5.898594	-3.893120	4.974407
225	H	-7.006791	-2.853821	6.256574
226	C	-9.978588	-0.376168	12.523677
227	H	-9.350684	-1.147291	12.067322
228	N	-10.488166	-0.876966	13.815893
229	O	-9.234402	0.841709	12.823931
230	H	-12.042999	0.222293	12.042025
231	N	-12.299586	1.073809	16.782338
232	C	-12.051044	1.774946	15.627733
233	N	-12.322582	3.098058	15.503943
234	N	-11.502457	1.180991	14.576139
235	C	-9.458976	1.843299	11.743561
236	H	-11.279489	1.996406	10.542161
237	C	-9.890982	3.152464	12.417436

238	H	-8.525203	1.983621	11.190344
239	C	-11.351092	-0.906146	15.869009
240	C	-11.137654	-0.107996	14.743382
241	H	-9.195596	3.349525	13.248285
242	H	-9.808376	3.968440	11.691086
243	O	-11.283157	3.109736	12.861678
244	C	-11.989227	-0.343668	17.019479
245	N	-10.830437	-2.189595	15.638125
246	O	-12.289638	-0.821344	18.118830
247	H	-9.832663	-2.940575	13.892145
248	C	-10.320035	-2.136938	14.416092
249	H	-12.713264	1.529586	17.592294
250	H	-12.825488	3.629119	16.199593
251	H	-12.014871	3.513697	14.605408
252	H	-11.370388	2.207265	13.362067
253	K	-5.327597	-0.176810	-0.512841
